# Expression and regulatory network of E3 ubiquitin ligase NEDD4 family in cancers

**DOI:** 10.1186/s12885-023-11007-w

**Published:** 2023-06-08

**Authors:** Liangzi Cao, Hao Li, Xiaofang Liu, Yubang Wang, Bowen Zheng, Chengzhong Xing, Naijin Zhang, Jingwei Liu

**Affiliations:** 1 Department of Anus and Intestine Surgery, the First Affiliated Hospital of China Medical University, 155# North Nanjing Street, Heping District, Shenyang City, 110001 Liaoning Province China; 2 Department of Clinical Laboratory, The First Hospital of China Medical University, Shenyang, China; 3 Department of Cardiology, The First Hospital of China Medical University, Shenyang, Liaoning China

**Keywords:** NEDD4, E3 ligase, Cancer, Ubiquitination

## Abstract

NEDD4 family represent an important group of E3 ligases, which regulate various cellular pathways of cell proliferation, cell junction and inflammation. Emerging evidence suggested that NEDD4 family members participate in the initiation and development of tumor. In this study, we systematically investigated the molecular alterations as well as the clinical relevance regarding NEDD4 family genes in 33 cancer types. Finally, we found that NEDD4 members showed increased expression in pancreas cancer and decreased expression in thyroid cancer. NEDD4 E3 ligase family genes had an average mutation frequency in the range of 0-32.1%, of which HECW1 and HECW2 demonstrated relatively high mutation rate. Breast cancer harbors large amount of NEDD4 copy number amplification. NEDD4 family members interacted proteins were enriched in various pathways including p53, Akt, apoptosis and autophagy, which were confirmed by further western blot and flow cytometric analysis in A549 and H1299 lung cancer cells. In addition, expression of NEDD4 family genes were associated with survival of cancer patients. Our findings provide novel insight into the effect of NEDD4 E3 ligase genes on cancer progression and treatment in the future.

**Supplementary Information:**

The online version contains supplementary material available at 10.1186/s12885-023-11007-w.

## Introduction

Ubiquitination is a key post-translational modification of proteins, which controls various aspects of protein functions including stability, location and signaling transduction [1, 2]. Ubiquitination is complex processes with multiple steps involving E1-ligases, E2-ligases and E3-ligases [3, 4]. E3 ligase is a distinct group of ligases that specifically recognize the substrates and link ubiquitin molecules to the substrates [5–7]. Subsequently, the ubiquitinated proteins are subjected to ubiquitin-proteasome system degradation or signaling transduction for functional pathways [8, 9].

NEDD4 family (neuronally expressed developmentally downregulated 4) represent an important group of E3 ligases, including 9 factors of WWP1, WWP2, HECW1, HECW2, ITCH, RPF1, NEDD4L, SMURF1 and SMURF2 [10, 11]. NEDD4 E3 ligases recognize and modify proteins by mono-ubiquitination or poly-ubiquitination, thus participating in various cellular pathways of cell proliferation, cell junction, ion channel modulation and inflammation [12, 13]. Through ubiquitinating diverse substrates, NEDD4 family members regulate different biological processes, such as cardiovascular diseases, inflammatory disorders, nervous system disorders, immunity and cancer [14, 15].

An increasing number of studies have reported the implication of NEDD4 family members in the initiation, progression and survival of cancer. For instance, NEDD4 ubiquitinated VDAC2 and VDAC3 to inhibit ferroptosis of melanoma [16]. NEDD4 maintained the characteristics of stem cell in breast cancer and promoted the growth and migration of breast cancer cells [17]. SMURF1 has been found to induce invasion of ovarian cancer cells by ubiquitinating ARHGAP26 [18]. In addition, SMURF2 was found to suppress colorectal cancer via degradation of YY1 protein as well as modulating c-myc signaling [19].

Although emerging evidence suggest that NEDD4 E3 ligase family members play critical roles in different aspects of tumor, no comprehensive analysis has been conducted to demonstrate the entire landscape of NEDD4 family members in all types of cancers. In this study, we analyzed the data of different cancers at various levels of DNA, RNA and proteins to generate the expression, copy number variation, protein interactions in relation to different NEDD4 members of tumor in detail. Furthermore, subsequent molecular experiments were performed to confirm our findings that NEDD4 were closely implicated in many biological processes of different cancers.

## Materials and methods

### NEDD4 E3 ligase family genes collection

Nine NEDD4 E3 ligase family genes were collected from review papers that have been published recently. We converted related gene symbols into Ensemble gene IDs and HGNC symbols based on GeneCards (https://www.genecards.org/).

### Genome-wide omics data over 33 cancer types from next-generation sequence data

We followed the methods of our previous study [20]. The analysis results considered the omics datasets from the TCGA Research Network (http://cancergenome.nih.gov/). 33 TCGA projects in total were analyzed, with each one representing a certain cancer type. These TCGA data that included the expression, the copy number alteration, the mutation, methylation as well as the clinical information (survival status and time, stage and grade) regarding Transcripts Per Kilobase Million (TPM) could be available on UCSC XENA(https://xenabrowser.net/).

### Differentially expressed genes (DEGs) identification

The Deseq2 package in R assisted in identifying DEGs for identifying the gene expression change in various cancer types. DEGs refer to those of which the adjusted P-values are less than 0.05 and the expression undergoes at least two-fold changes.

**Proteomics data regarding pan cancer from protein expression data.**

We obtained the protein expression data regarding NEDD4 E3 ligase family genes from the protein atlas datasets (https://www.proteinatlas.org/). The protein expression regarding NEDD4 E3 ligase family genes from 20 cancer types were analyzed, including common cancers of breast cancer, lung cancer and colon cancer.

### Enrichment analysis of NEDD4-related proteins

The NEDD4-related proteins were achieved from BioGRID (Biological General Repository for Interaction Datasets), a public database that archives and disseminates genetic and protein interaction data from model organisms and humans. Furthermore, enrichment analysis including BP (biological process), MF (molecular function), CC (Cellular Component) and KEGG (Kyoto Encyclopedia of Genes and Genomes) were analyzed by ClusterProfiler package of R language. Cytoscape was used to visualize the interaction network of NEDD4 members with interacted proteins. In addition, the Pearson Correlation Coefficient (PCC) between NEDD4 members and the pathway was calculated, aiming at identifying those that could affect specific pathway.

### TIMER and CMAP analysis

TIMER (http://timer.cistrome.org) is a database used to analyze the relationship between gene expression and immune cells in tumor tissues. We used the TIMER database to analyze the relationship between NEDD4 family members and immune cells. Furthermore, we used the CMAP (https://clue.io/about) database to investigate which small molecule chemicals are associated with NEDD4 family members.

### Clinical significance exhibited by NEDD4 E3 ligase family genes

For confirming the effect of NEDD4 E3 ligase family genes’ expression on patients’ survival, the median expression level regarding each hypoxia related gene was taken into account for dividing patients into 2 groups. The log-rank test assisted in examining their difference in the survival rate. The P-values < 0.05 reported statistical significance. Kaplan Meier method served for the prognosis analysis, which adopted the HR value. HR > 1 and HR < 1 indicate that high gene expression leads to poor and good prognosis, respectively.

### Cell culture

H1299 and A549 cells were purchased from Cell Bank in Chinese Academy of Sciences Shanghai. H1299 cells were cultured in RPMI 1640 medium; A549 cells were cultured in high-glucose Dulbecco’s modified Eagle’s medium (DMEM), supplemented with10% fetal bovine serum (FBS)(CLARK, Australia), penicillin (100U),and streptomycin (100 g/ml).

### Antibodies and reagents

Antibodies used in this study include β-actin (AC004, ABclonal), Flag (SG4110-16, Shanghai Genomics Technology), Myc (SG4110-18, Shanghai Genomics Technology), Smurf1(ab300408, Abcam), Smurf2(#12,024, Cell Signaling Technology (CST)), p62(#23,214, CST), p53(sc-126, Santa Cruz), Akt(#4691, CST), p-Akt(#4060, CST).

### Western blot

In order to confirm the enrichment analysis results of NEDD4-interacted proteins, we performed transfection of Smurf1 and Smurf2 or siRNAs for Smurf1 and Smuf2 in A549 or H1299 cells, followed by western blot of p53, Akt, p-Akt (S473), p62 and LC3 proteins. Cells were lysed on ice with IP lysis buffer supplemented with protease inhibitor cocktails, then the lysed protein was harvested by centrifugation. Protein samples were separated on 10% SDS PAGE and transferred to PVDF membrane (Millipore, IPVH00010) for two hours at 80 V. After block in milk for one hour at room temperature, the membranes were probed with specific primary antibodies at 4 °C overnight. The membranes were then washed with TBST three times followed by incubation with HRP-conjugated secondary antibody at room temperature for two hours. After three washes, bands were detected by enhanced chemi-luminescence detection kit (Thermo Fisher Scientific, 32,106) and visualized via the DNR western blot detection system.

### Flow cytometric analysis

To investigate cell apoptosis, H1299 or A549 cells were subjected to incubation with PI and FITC-Annexin V (BD Phamingen, 556,547). The percentage of apoptotic cells was then measured according to the manufacturer’s protocol.

## Results

### Expression profile regarding NEDD4 E3 ligase family genes of various cancer types

**Fig. 1:**
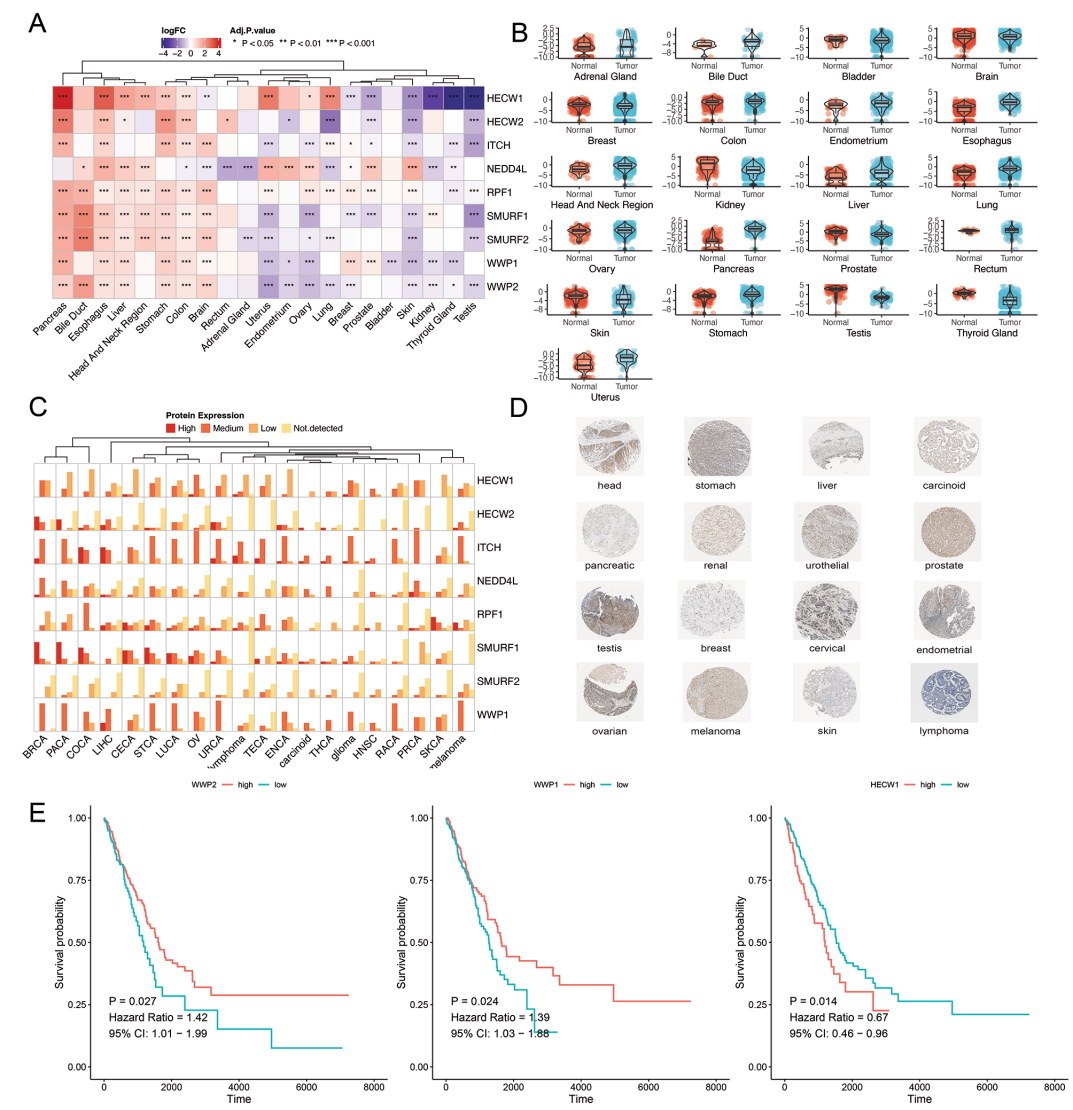
Expression profile of NEDD4 family members across different cancer types. **A**, Expression of NEDD4 family members in different cancer and normal samples. **B**, HECW1 expression in different cancers between cancer and normal tissues. **C**, NEDD4 family members protein expression across various cancer types. Each gene expression in one cancer were divided into four groups of high expression, medium expression, low expression and not detected. **D**, NEDD4 E3 ligase family protein expression in 16 cancer types based on immunohistochemistry staining. **E**, Association of NEDD4 E3 ligase family genes methylation with lung cancer prognosis

The study identified 9 NEDD4 E3 ligase family genes after searching the published review papers. The count data of TCGA were employed for confirming their differential expressions for various cancer types. NEDD4 E3 ligase family genes were distributed heterogeneously in various cancer types (Fig. 1A): NEDD4 E3 ligase family genes presented high expression in some tumors including pancreas cancer, esophagus cancer, gastric cancer and colon cancer; while NEDD4 E3 ligase family genes showed low expression in certain tumors of kidney, thyroid, and testis cancer. Table S1 gives the detailed LogFC and P value changes. The differential expression regarding HECW1 in each cancer was visualized (Fig. 1B). The immunohistochemistry results from the Protein Atlas database gave the protein expression regarding NEDD4 E3 ligase family genes of the 33 cancer types (Fig. 1C). Figure 1D illustrates the immunohistochemistry results regarding HECW1 that represents the protein expression. Finally, we analyzed the relationship between methylation of NEDD4 family members and tumor prognosis. We summarized the analyzing results in Table S2, and representative relationship between methylation of NEDD4 family genes and lung cancer prognosis was shown in Fig. 1E.

### Pan-cancer genetic variation regarding NEDD4 E3 ligase family genes

**Fig. 2:**
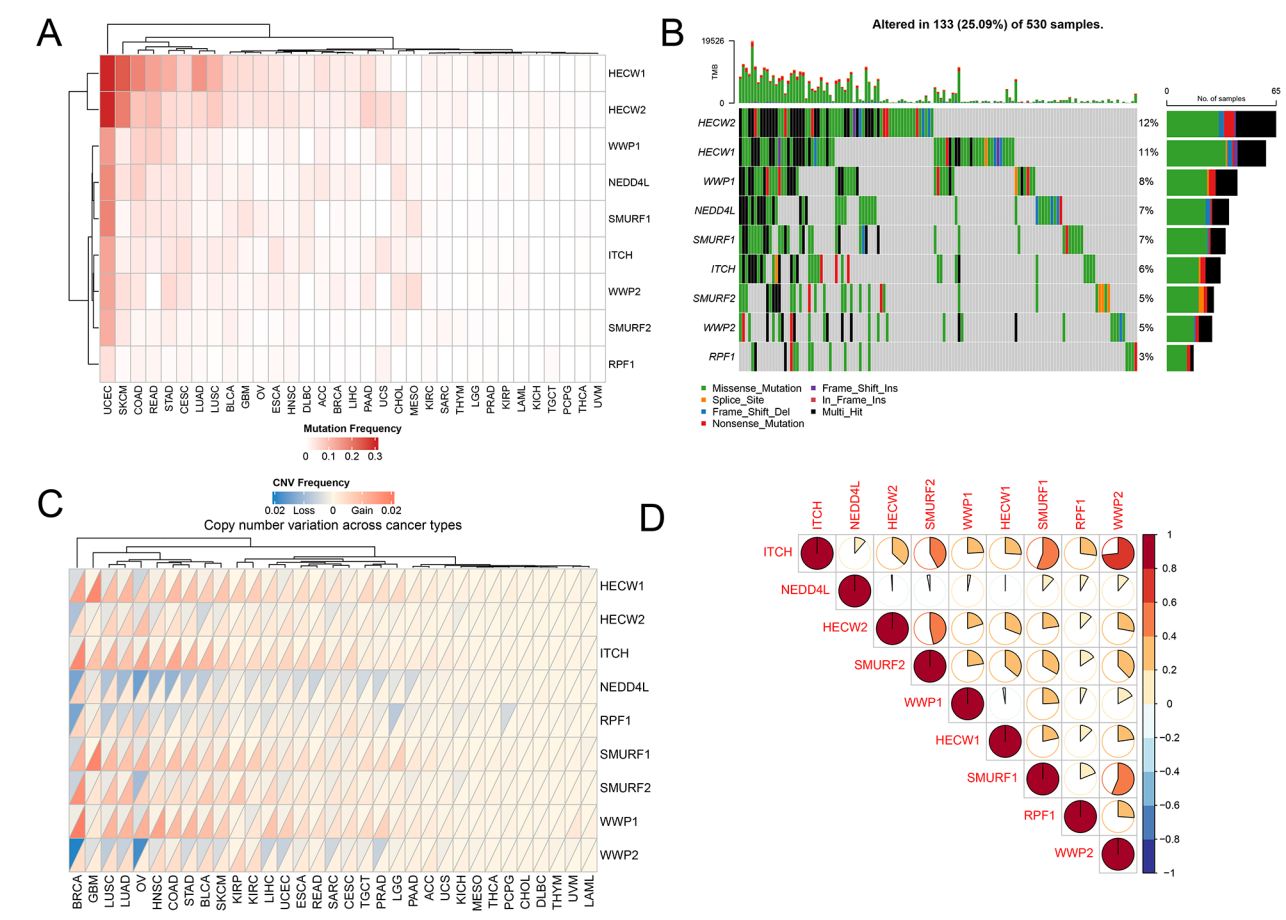
Pan-cancer genetic alternations of NEDD4 E3 ligase family genes. **A**, Pan-cancer mutation frequency of NEDD4 E3 ligase family genes. Red color represent high mutation frequency. **B**, Oncoplot for NEDD4 E3 ligase family genes in UCEC. NEDD4 E3 ligase family genes showed the most frequent mutation in UCEC. **C**, The copy number variations frequency of NEDD4 E3 ligase family genes in different cancers. Red color represents increased CNVs while blue color represents decreased CNVs. **D**, Correlation between NEDD4 E3 ligase family genes. Red color represent high correlation coefficient

Analysis results of the mutation frequency regarding NEDD4 E3 ligase family genes found their frequent mutation in UCEC (Fig. 2A). NEDD4 E3 ligase family genes had an average mutation frequency in the range of 0-32.1%, and that of the HECW1 and HECW2 were remarkably high (Table S3). For many cancers like TGCT, PCPG, THCA and UVM, NEDD4 E3 ligase family genes seldom reported mutation. For obtaining more data of NEDD4 E3 ligase family gene mutations, oncoplot was used for visualizing details of the mutation in UCEC (Fig. 2B). We also examined the copy number change regarding NEDD4 E3 ligase family genes (Fig. 2C) (Table S4). Specific to each cancer type, BRCA exhibited large copy number amplification while LAML nearly saw no CNV. Furthermore, we performed correlation analysis among the NEDD4 E3 ligase family genes, which was summarized in Fig. 2D and Table S5.

### Association between NEDD4 E3 ligase family genes and cancer-related pathways

**Fig. 3:**
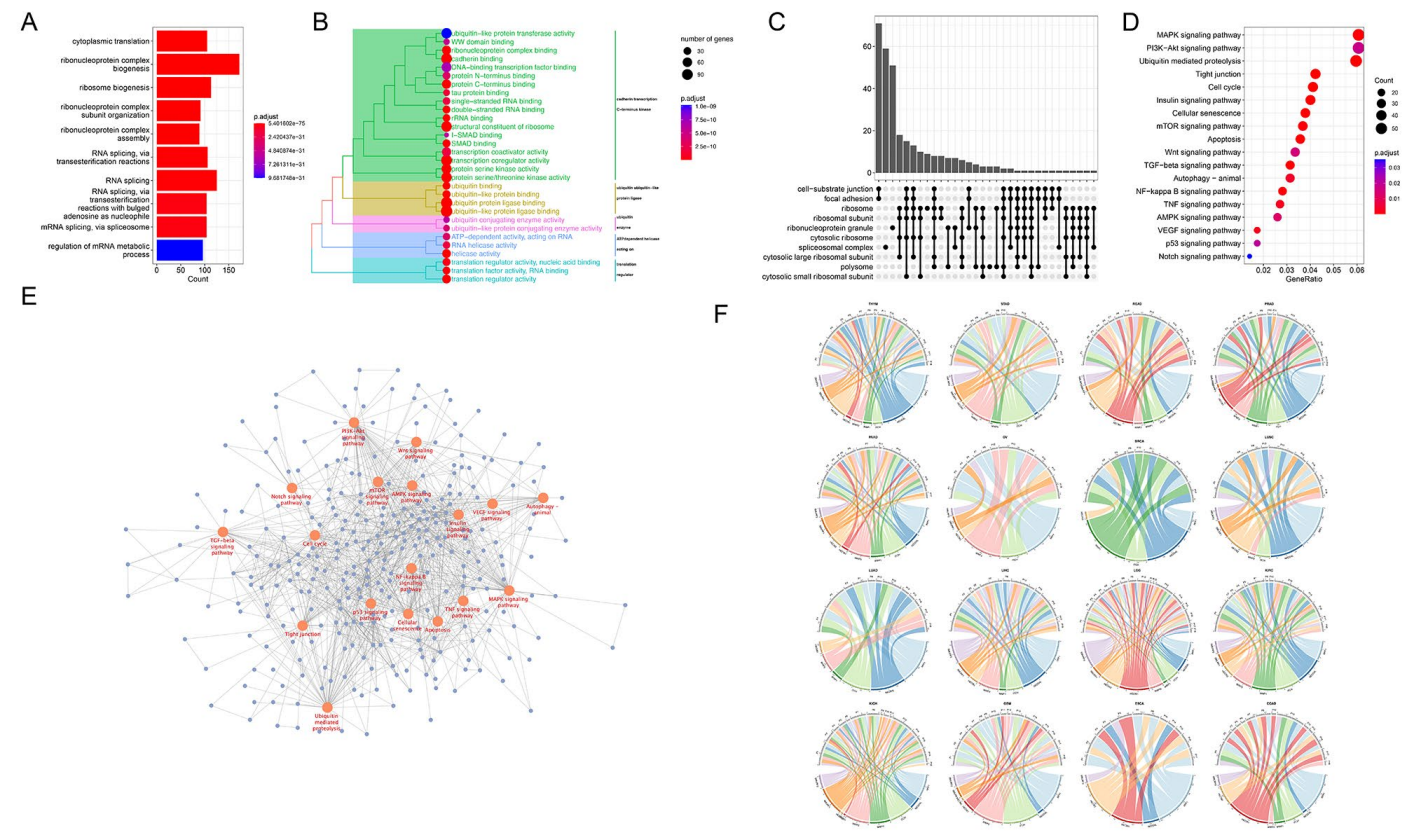
Enrichment analysis of NEDD4 E3 ligase family genes in cancer. **A**, BP (biological process) Enrichment analysis of NEDD4 E3 ligase family genes in cancer. **B**, MF (molecular function) Enrichment analysis of NEDD4 E3 ligase family genes in cancer. **C**, CC (Cellular Component) Enrichment analysis of NEDD4 E3 ligase family genes in cancer. **D**, KEGG (Kyoto Encyclopedia of Genes and Genomes) Enrichment analysis of NEDD4 E3 ligase family genes in cancer. **E**, Visualization of NEDD4 E3 ligase family members related pathways. **F**, The correlations between NEDD4 members and different pathways in various types of cancers

For elucidating the molecular significance exhibited by NEDD4 E3 ligase family genes in cancer, we analyzed the relation between NEDD4 E3 ligase family genes and pathways related to cancer. NEDD4 E3 ligase family genes mainly affected biological processes (BP) including cytoplasmic translation, ribonucleoprotein complex and ribosome biogenesis (Fig. 3A). NEDD4 E3 ligase family genes enriched in molecular functions (MF) of ubiquitin-like protein transferase activity, WW domain binding, ribonucleoprotein complex binding (Fig. 3B). For Cellular Component (CC), NEDD4 E3 ligase family genes demonstrated enrichment in cell − substrate junction, focal adhesion and ribosome (Fig. 3C). KEGG pathway analysis showed that NEDD4 members participated in the regulation of multiple pathways including Akt, p53, autophagy and apoptosis (Fig. 3D) (Table S6), which were further visualized in Fig. 3E. In addition, the correlations between NEDD4 members and different pathways in various types of cancers were summarized in Fig. 3F.

### Prognostic significance exhibited by NEDD4 E3 ligase family genes

**Fig. 4:**
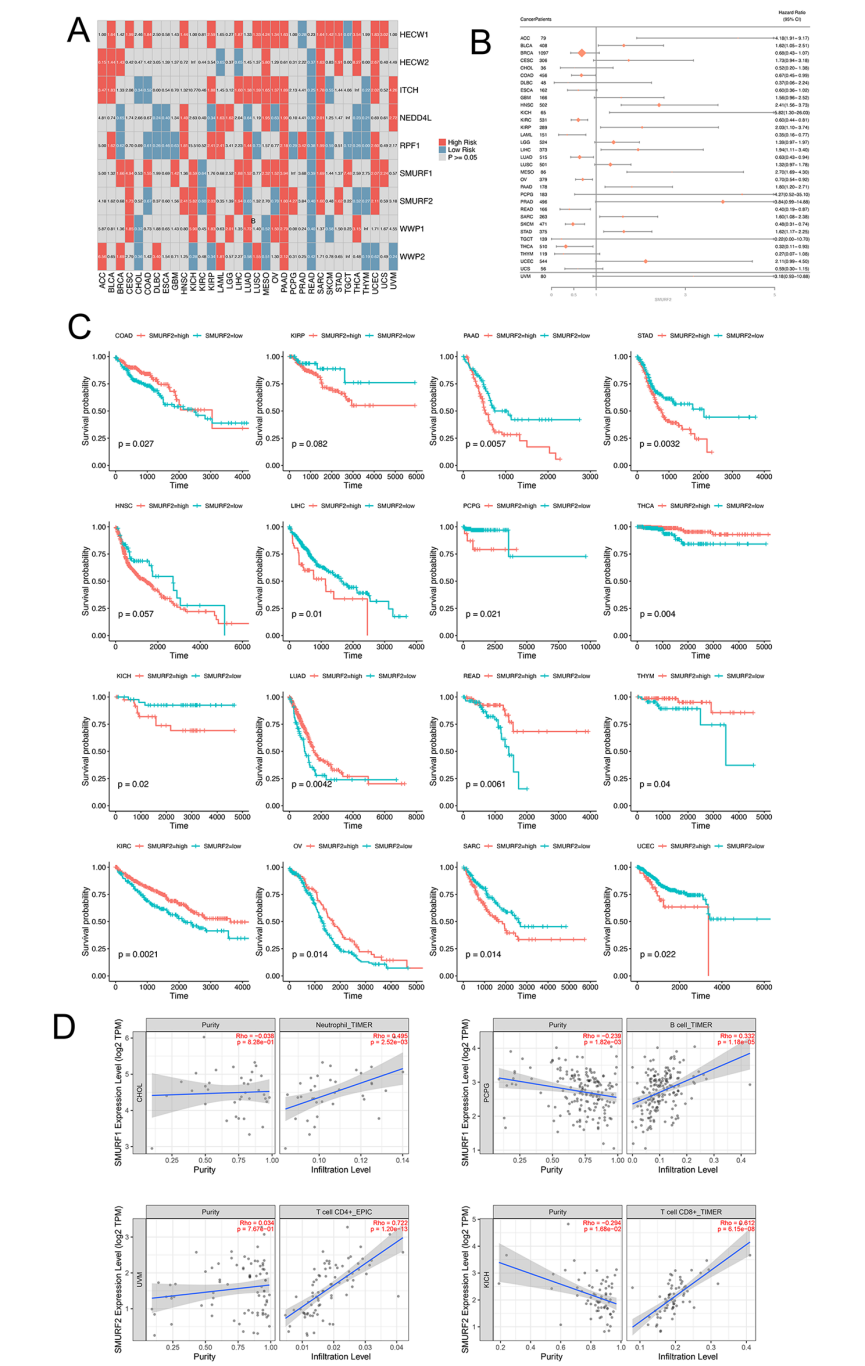
Prognostic significance of NEDD4 E3 ligase family genes. **A**, Summary of the correlation between expression of NEDD4 E3 ligase family genes and survival of different cancers. Unfavorable survival is presented in red color, while favorable survival is presented in blue color. **B**, Forest plot for the prognostic analysis of Smurf2 across various cancer types. The HR and 95CI for each cancer were listed in the figure. **C**, The survival plot of relationship between Smurf2 and cancer prognosis in different types of cancers including COAD, KIRP, PAAD, STAD, HNSC, LIHC, PCPG, THCA. **D**, Correlation analysis of immune cells with NEDD4 members Smurf1 and Smurf2

The cox regression results showed the relation between NEDD4 E3 ligase family genes and the cancer prognosis (Fig. 4A) (Table S7). HECW1 expression correlated with worse survival in most of cancers. Besides, some NEDD4 E3 ligase family genes might affect the prognosis regarding different cancer types to different extents such as WWP2 and Smurf2. To be specific, Smurf2 affected the cancer prognosis in different cancer types, and forest plot was adopted for illustrating how they helped to predict cancer prognosis (Fig. 4B). In addition, the survival plot of relationship between Smurf2 and cancer prognosis in different types of cancers was shown (Fig. 4C).

### TIMER and CMAP analysis

Finally, we analyzed the relationship of Smurf1 and Smurf 2 with immune cells in tumor tissues. The analysis revealed that Smurf1 is mainly associated with B cells and neutrophils. Smurf2, on the other hand, was mainly related to CD4^+^ and CD8^+^ T cells (Fig. 4D). In addition, to predict the potential relationship between NEDD4 gene families and small molecule chemicals, we used the CMAP database to analyze which small molecule substances might exert effect on NEDD4s. After analysis, we found that withaferin A, ellipticine, flupentixol and perphenazine were the most important small molecule chemical affecting NEDD4s (Table S8).

### Association of NEDD4 family members with enrichment pathways of akt, p53 and autophagy in cancer

**Fig. 5:**
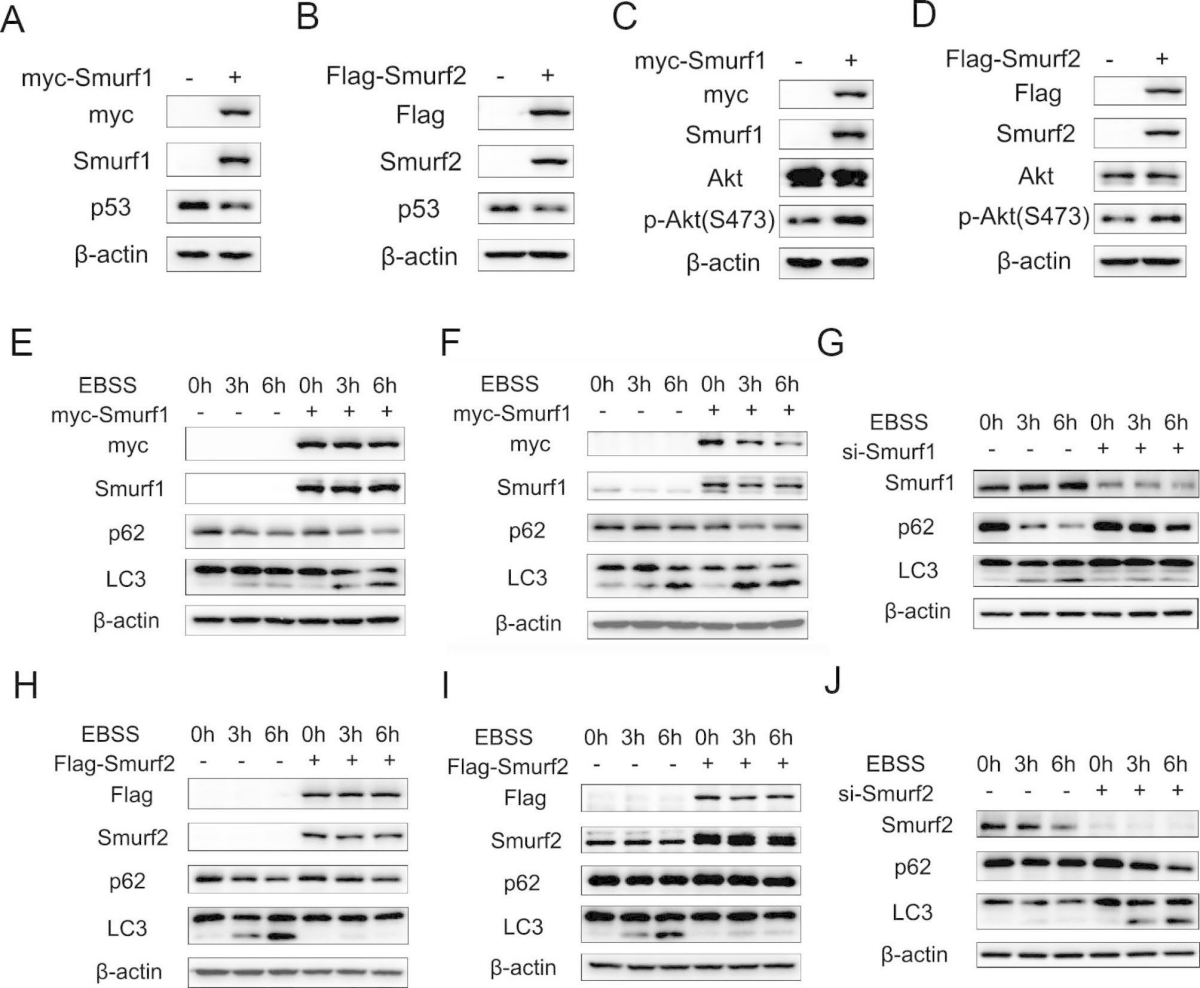
Smurf1 and Smurf2 regulate p53, Akt and autophagy signaling pathway. **A**, Western blot analysis demonstrating p53 protein levels with myc-Smurf1 plasmids transfected in A549 cells. **B**, Western blot analysis demonstrating p53 protein levels with Flag-Smurf2 plasmids transfected in A549 cells. **C**, Western blot analysis demonstrating Akt, p-Akt (S473) protein levels with myc-Smurf1 plasmids transfected in A549 cells. **D**, Western blot analysis demonstrating Akt, p-Akt (S473) protein levels with Flag-Smurf2 plasmids transfected in A549 cells. **E**, Western blot analysis demonstrating p62 and LC3 protein levels with myc-Smurf1 plasmids transfected in A549 cells after treatment of EBSS for indicated time. **F**, Western blot analysis demonstrating p62 and LC3 protein levels with myc-Smurf1 plasmids transfected in H1299 cells after treatment of EBSS for indicated time. **G**, Western blot analysis demonstrating p62 and LC3 protein levels with si-Smurf1 transfected in H1299 cells after treatment of EBSS for indicated time. **H**, Western blot analysis demonstrating p62 and LC3 protein levels with Flag-Smurf2 plasmids transfected in A549 cells after treatment of EBSS for indicated time. **I**, Western blot analysis demonstrating p62 and LC3 protein levels with Flag-Smurf2 plasmids transfected in H1299 cells after treatment of EBSS for indicated time. **J**, Western blot analysis demonstrating p62 and LC3 protein levels with si-Smurf2 transfected in H1299 cells after treatment of EBSS for indicated time

In order to confirm the results of enrichment analysis of NEDD4 family members-interacted proteins, we transfected plasmids of Smurf1, Smurf2 in A549 and H1299 lung cancer cells. The results suggested that NEDD4 members Smurf1 and Smurf2 suppresses p53 pathway (Fig. 5A, B) as p53 protein levels decreased. In addition, NEDD4 members Smurf1 and Smurf2 promote Akt pathway as overexpression of Smurf1 and Smurf2 increase p-Akt (S473) levels (Fig. 5C, D). Under the treatment of (Earle’s Balanced Salt Solution) EBSS (a stimulation of starvation for autophagy), autophagy was facilitated after transfection of NEDD4 members Smurf1 (Fig. 5E, F); autophagy was inhibited after transfection of NEDD4 members Smurf2 (Fig. 5H, I). Furthermore, under the treatment of EBSS (a stimulation of starvation for autophagy), autophagy was inhibited after transfection of Smurf1 siRNAs (Fig. 5G); autophagy was promoted after transfection of Smurf2 siRNAs (Fig. 5J).

### Effect of NEDD4 family members on apoptosis in cancer

**Fig. 6:**
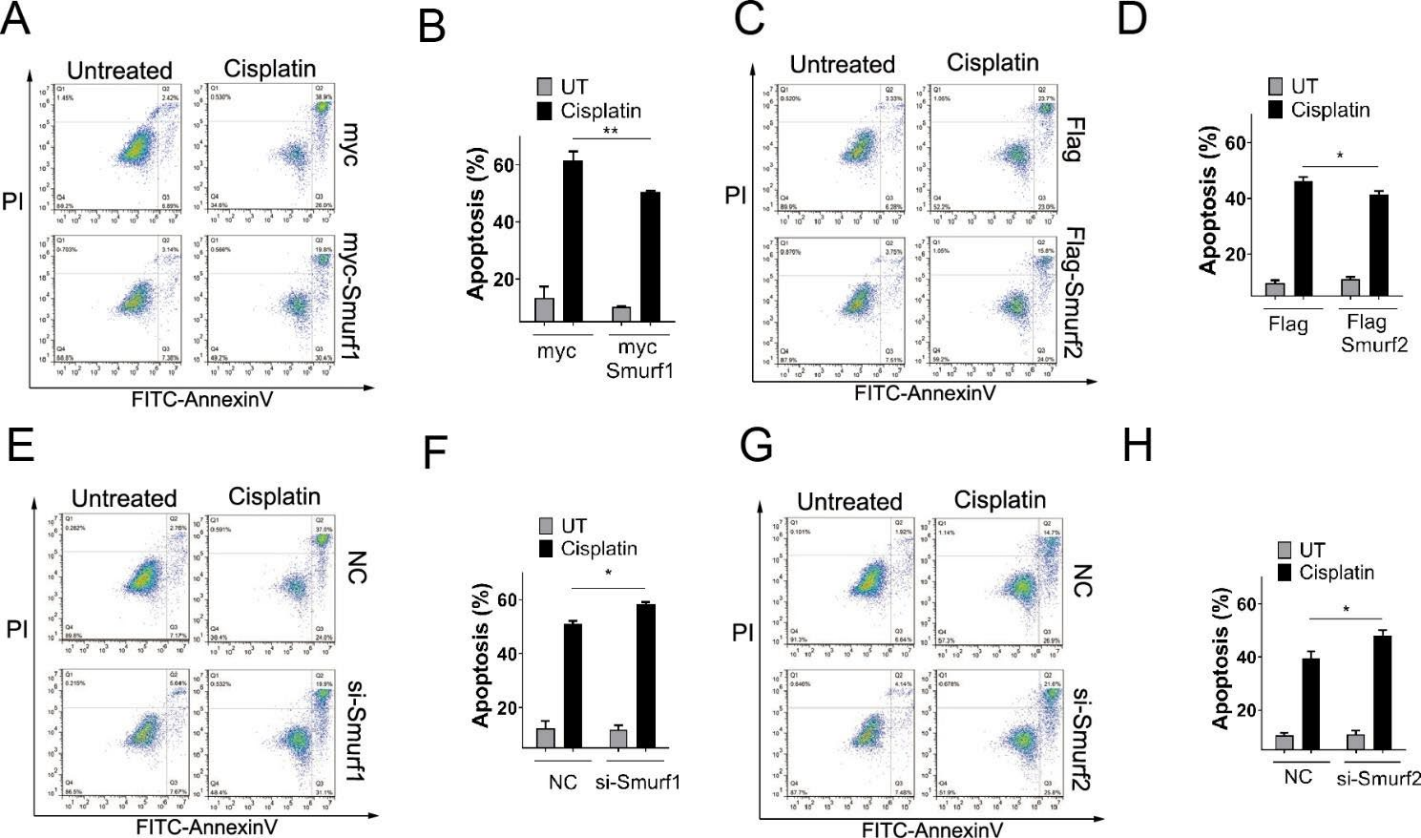
Smurf1 and Smurf2 regulate apoptosis. **A-B**, H1299 cells transfected with myc or myc-Smurf1 were treated with cisplatin (20µM) for 24 h followed by staining with PI and FITC-Annexin V, and analyzed by fluorescence-activated cell sorting (FACS). Scatter graph represents percentage of apoptotic cells from three independent experiments. **C-D**, H1299 cells transfected with Flag or Flag-Smurf2 were treated with cisplatin (20µM) for 24 h followed by staining with PI and FITC-Annexin V, and analyzed by fluorescence-activated cell sorting (FACS). Scatter graph represents percentage of apoptotic cells from three independent experiments. **E-F**, H1299 cells transfected with NC or si-Smurf1 were treated with cisplatin (20µM) for 24 h followed by staining with PI and FITC-Annexin V, and analyzed by fluorescence-activated cell sorting (FACS). Scatter graph represents percentage of apoptotic cells from three independent experiments. **G-H**, H1299 cells transfected with NC or si-Smurf2 were treated with cisplatin (20µM) for 24 h followed by staining with PI and FITC-Annexin V, and analyzed by fluorescence-activated cell sorting (FACS). Scatter graph represents percentage of apoptotic cells from three independent experiments

In order to confirm the effect of NEDD4 family members on apoptosis of cancer, we performed flow cytometric analysis. After transfection of NEDD4 family members Smurf1 and Smurf2, the lung cancer cells H1299 were subjected to cisplatin (a commonly used chemotherapy drug) treatment to detect the difference of apoptosis in relation to Smurf1 and Smurf2. Finally, we found that both Smurf1 and Smurf2 suppressed apoptosis in H1299 lung cancer cells (Fig. 6A-D). On contrast, transient silence of Smurf1 and Smurf2 by si-RNAs promoted apoptosis in H1299 lung cancer cells (Fig. 6E-H).

## Discussion

E3 ligases is a distinct group of ligases that specifically recognize the substrates and link ubiquitin molecules to the substrates, of which NEDD4 E3 ligase family participates in various cellular pathways of cell proliferation, cell junction and inflammation. Although increasing evidence has reported the close implication of NEDD4 family members in multiple aspects of cancer. In this study, we systematically investigated the molecular alterations as well as the clinical relevance regarding NEDD4 family genes in 33 cancer types. The results described the landscape of NEDD4 expression, mutation and copy number variation as well as suggested the biological processes and pathways in relation to NEDD4-interacted proteins.

Analysis of the nine NEDD4 E3 ligase family genes of TCGA data suggested that NEDD4 E3 ligase family genes were distributed heterogeneously in various cancer types. NEDD4 E3 ligase family genes presented high expression in some tumors including pancreas cancer, esophagus cancer, gastric cancer and colon cancer; while NEDD4 E3 ligase family genes showed low expression in certain tumors of kidney, thyroid, and testis cancer. In addition, NEDD4 E3 ligase family genes had an average mutation frequency in the range of 0-32.1%, and that of the HECW1 and HECW2 were remarkably high. Furthermore, BRCA exhibited large copy number amplification while LAML nearly saw no CNV. We also analyzed the relationship between methylation of NEDD4 family members and tumor prognosis, which shows involvement of NEDD4s methylation in relation to tumor prognosis. The analysis of Smurfs with immune cells revealed that Smurf1 is mainly associated with B cells and neutrophils. Smurf2 was mainly related to CD4^+^ and CD8^+^ T cells. In addition, we found that withaferin A, ellipticine, flupentixol and perphenazine were the most important small molecule chemical affecting NEDD4s.

Next, we analyzed the relation between NEDD4 E3 ligase family genes and pathways related to cancer in order to elucidate the molecular significance. The enrichment analysis indicated biological processes including cytoplasmic translation, ribonucleoprotein complex and ribosome biogenesis as well as KEGG pathways including Akt, p53, autophagy and apoptosis. Our results in A549 lung cancer cells suggested that NEDD4 members Smurf1 and Smurf2 suppresses p53 pathway as p53 protein levels decreased. Smurf1 was found to promote p53 degradation via stabilizing its E3 ligase MDM2 [21]. In addition, NEDD4 members Smurf1 and Smurf2 promote Akt pathway as overexpression of Smurf1 and Smurf2 increase p-Akt levels. Previous study reported that Smurf1 overexpression activates PI3K/Akt signaling pathway [22]. Under the treatment of starvation, autophagy was facilitated after transfection of NEDD4 members Smurf1; autophagy was inhibited after transfection of NEDD4 members Smurf2. The analysis of si-Smurf1 and si-Smurf2 confirmed the effect of Smurf1 and Smurf2 in autophagy. The relationship between NEDD4s and autophagy still require further mechanism studies to clarify, as relevant reports were limited [23–26]. Furthermore, our results of flow cytometric analysis suggested that both Smurf1 and Smurf2 suppressed apoptosis in H1299 lung cancer cells, which were in consistence with previous reports of the involvement of Smurf1 and Smurf2 in apoptosis [27–31].

Finally, we explored the prognostic significance of NEDD4 E3 ligase family genes. HECW1 expression correlated with worse survival in most of cancers. Besides, some NEDD4 E3 ligase family genes might affect the prognosis regarding different cancer types to different extents such as WWP2 and Smurf2. NEDD4 expression has been reported to correlated with breast cancer development and worse survival [32]. In another study of primary breast cancer, NEDD4-1 expression were not significantly related with clinical outcomes of HER2-amplified breast cancer [33]. Low expression of NEDD4L was found to be associated with poor survival of gastric cancer patients [34]. In addition, overexpression of Nedd4-1 was found to associated with an extremely low survival rate of gastric cardia adenocarcinoma [35]. Further larger-scale studies are required to elucidate the exact correlation between NEDD4 family members and different cancers.

## Conclusion

In summary, our study systematically demonstrated the expression, mutation, copy number variation, functional pathways and prognostic value of NEDD4 E3 ligase family genes across multiple cancers. The expression of NEDD4 E3 ligase family genes show significant association with pathways including MAPK, wnt, Akt, p53, autophagy and apoptosis as well as the potential to predict prognosis of cancer patients. These findings provide novel evidence for the investigation of NEDD4 E3 ligase family genes in the development and therapy of cancer.

## Electronic supplementary material

Below is the link to the electronic supplementary material.

Supplementary Material 1

Supplementary Material 2

## Data Availability

Not Applicable.

## Data Availability

The datasets generated and/or analysed during the current study are available in the TCGA Research Network repository, (http://cancergenome.nih.gov/).
