# Supplementary material for: Expression and regulatory network of E3 ubiquitin ligase NEDD4 family in cancers
Source: BMC Cancer. 2023 Jun 8;23:526. doi: 10.1186/s12885-023-11007-w (PMC10251597; doi:10.1186/s12885-023-11007-w)
Supplement: Supplementary file 1 — Supplementary Material 1 [file 12885_2023_11007_MOESM1_ESM.docx]

Supplementary Table 1. Differential expression analysis of NEDD4 family genes.

| Cancer | EnsID | Gene | logFC | AveExpr | t | P.Value | adj.P.Val | B |
| --- | --- | --- | --- | --- | --- | --- | --- | --- |
| Adrenal Gland | ENSG00000049759.16 | NEDD4L | -1.3071546 | 3.23234341 | -6.8817521 | 7.1692E-11 | 7.1916E-10 | 13.9250515 |
| Adrenal Gland | ENSG00000108854.15 | SMURF2 | -0.6203039 | 2.31031951 | -3.8397724 | 0.00016444 | 0.00068845 | -0.252121 |
| Adrenal Gland | ENSG00000138411.10 | HECW2 | -0.3629141 | -0.2767883 | -2.2488095 | 0.02559665 | 0.06703662 | -4.8754659 |
| Adrenal Gland | ENSG00000198373.12 | WWP2 | -0.2847574 | 4.57302829 | -1.6821448 | 0.09407527 | 0.20430648 | -5.9691297 |
| Adrenal Gland | ENSG00000123124.13 | WWP1 | 0.25036828 | 3.49245171 | 1.46276296 | 0.14507569 | 0.29429598 | -6.3102775 |
| Adrenal Gland | ENSG00000198742.9 | SMURF1 | -0.2055908 | 3.09973902 | -1.1939071 | 0.23390484 | 0.42505032 | -6.6646667 |
| Adrenal Gland | ENSG00000002746.14 | HECW1 | 0.4667275 | -5.6696785 | 1.09356058 | 0.27544113 | 0.47809828 | -6.7788188 |
| Adrenal Gland | ENSG00000117133.10 | RPF1 | -0.1221367 | 3.97516098 | -0.7305396 | 0.46590102 | 0.70901242 | -7.1088523 |
| Adrenal Gland | ENSG00000078747.12 | ITCH | 0.01668872 | 3.0943122 | 0.10396377 | 0.91730054 | 1 | -7.3702163 |
| Bile Duct | ENSG00000198373.12 | WWP2 | 1.97765833 | 4.10422667 | 13.8089918 | 1.7798E-17 | 3.2142E-15 | 29.6655435 |
| Bile Duct | ENSG00000198742.9 | SMURF1 | 2.37845833 | 3.53225556 | 9.81474319 | 1.3907E-12 | 3.6619E-11 | 18.4651906 |
| Bile Duct | ENSG00000117133.10 | RPF1 | 1.50518611 | 4.47767111 | 8.19221146 | 2.3883E-10 | 3.5147E-09 | 13.3376788 |
| Bile Duct | ENSG00000108854.15 | SMURF2 | 2.52224722 | 2.27273111 | 7.00843979 | 1.1994E-08 | 1.2165E-07 | 9.44191962 |
| Bile Duct | ENSG00000049759.16 | NEDD4L | 0.7591333 | 4.29254 | 2.943403 | 0.00519818 | 0.01843696 | -3.21025 |
| Bile Duct | ENSG00000138411.10 | HECW2 | 0.6420833 | 0.06682222 | 1.515808 | 0.1368263 | 0.3307514 | -6.088451 |
| Bile Duct | ENSG00000002746.14 | HECW1 | 1.225514 | -3.853411 | 1.240955 | 0.2212958 | 0.4913731 | -6.457619 |
| Bile Duct | ENSG00000123124.13 | WWP1 | 0.3209167 | 4.862867 | 1.019955 | 0.3134081 | 0.6347907 | -6.704508 |
| Bile Duct | ENSG00000078747.12 | ITCH | 0.05978611 | 3.286984 | 0.2642391 | 0.7928479 | 1 | -7.192122 |
| Bladder | ENSG00000123124.13 | WWP1 | -0.7146607 | 3.84475473 | -3.9820164 | 8.0122E-05 | 0.00056481 | 0.7005705 |
| Bladder | ENSG00000049759.16 | NEDD4L | 0.28230892 | 4.78258374 | 1.39597984 | 0.16343512 | 0.38716807 | -6.0776914 |
| Bladder | ENSG00000117133.10 | RPF1 | 0.1159719 | 4.86797847 | 1.03550566 | 0.30101016 | 0.60321683 | -6.5121484 |
| Bladder | ENSG00000198742.9 | SMURF1 | -0.1310477 | 3.46565188 | -0.94353 | 0.34593545 | 0.65990313 | -6.6024842 |
| Bladder | ENSG00000198373.12 | WWP2 | -0.1030792 | 4.18425578 | -0.8520409 | 0.3946619 | 0.71992328 | -6.6840448 |
| Bladder | ENSG00000002746.14 | HECW1 | -0.3095948 | -1.4113633 | -0.6662957 | 0.50557658 | 0.831367 | -6.8241385 |
| Bladder | ENSG00000138411.10 | HECW2 | 0.10662185 | -0.6788769 | 0.47731035 | 0.63338172 | 0.93591732 | -6.9315593 |
| Bladder | ENSG00000108854.15 | SMURF2 | 0.05140202 | 3.02360053 | 0.31654887 | 0.75173833 | 1 | -6.9950168 |
| Bladder | ENSG00000078747.12 | ITCH | -0.0403004 | 3.62982583 | -0.2609971 | 0.79421872 | 1 | -7.0109728 |
| Brain | ENSG00000117133.10 | RPF1 | 1.15652411 | 3.89988486 | 23.0877742 | 1.234E-103 | 1.285E-102 | 225.308955 |
| Brain | ENSG00000108854.15 | SMURF2 | 1.02362945 | 2.72134768 | 21.4461131 | 3.862E-91 | 3.3866E-90 | 196.59711 |
| Brain | ENSG00000078747.12 | ITCH | 1.07269168 | 3.17840148 | 21.0768197 | 2.1064E-88 | 1.7843E-87 | 190.309757 |
| Brain | ENSG00000198373.12 | WWP2 | 0.86470936 | 4.06962845 | 16.8499517 | 2.6239E-59 | 1.4796E-58 | 123.503415 |
| Brain | ENSG00000198742.9 | SMURF1 | 0.57442604 | 2.69492904 | 11.4779153 | 1.7208E-29 | 6.2753E-29 | 55.1897596 |
| Brain | ENSG00000049759.16 | NEDD4L | -0.3302601 | 3.63328684 | -5.5827561 | 2.7245E-08 | 6.1638E-08 | 7.04602565 |
| Brain | ENSG00000123124.13 | WWP1 | 0.18072965 | 3.32881097 | 3.55200739 | 0.00039205 | 0.00073543 | -2.1193662 |
| Brain | ENSG00000002746.14 | HECW1 | -0.3236274 | 0.74064266 | -3.4072365 | 0.00067055 | 0.00123815 | -2.6198079 |
| Brain | ENSG00000138411.10 | HECW2 | -0.0127364 | 1.02505483 | -0.2082341 | 0.83506955 | 0.99222322 | -8.3846378 |
| Breast | ENSG00000117133.10 | RPF1 | 0.38584683 | 4.99303299 | 11.7352269 | 2.2016E-30 | 1.2352E-29 | 57.4230313 |
| Breast | ENSG00000123124.13 | WWP1 | 0.74304356 | 5.57670542 | 9.33932328 | 3.7433E-20 | 1.595E-19 | 34.0750755 |
| Breast | ENSG00000198742.9 | SMURF1 | -0.3731428 | 3.37810713 | -9.1077825 | 2.8642E-19 | 1.1887E-18 | 32.0633422 |
| Breast | ENSG00000198373.12 | WWP2 | -0.3136369 | 4.37724738 | -7.1223369 | 1.7014E-12 | 5.6339E-12 | 16.6941911 |
| Breast | ENSG00000002746.14 | HECW1 | -0.5192409 | -2.9119435 | -4.0069003 | 6.4799E-05 | 0.00014943 | -0.2338267 |
| Breast | ENSG00000078747.12 | ITCH | 0.13047682 | 3.88775943 | 2.5969793 | 0.00950456 | 0.01815218 | -4.8512427 |
| Breast | ENSG00000049759.16 | NEDD4L | 0.1542436 | 4.79114505 | 2.54927254 | 0.01090201 | 0.02068982 | -4.9734134 |
| Breast | ENSG00000108854.15 | SMURF2 | -0.0742208 | 3.41399189 | -1.3547027 | 0.17573363 | 0.27110884 | -7.298202 |
| Breast | ENSG00000138411.10 | HECW2 | -0.0811108 | 0.94313235 | -1.1663267 | 0.24368352 | 0.36206016 | -7.5353797 |
| Colon | ENSG00000138411.10 | HECW2 | 1.08925539 | -1.1208905 | 10.8015079 | 4.3783E-25 | 2.7638E-24 | 45.5728081 |
| Colon | ENSG00000078747.12 | ITCH | 0.59108875 | 3.61370969 | 6.27472218 | 6.4816E-10 | 2.1983E-09 | 11.1031254 |
| Colon | ENSG00000123124.13 | WWP1 | 0.57988681 | 4.22203218 | 5.94926955 | 4.4497E-09 | 1.4464E-08 | 9.22368036 |
| Colon | ENSG00000117133.10 | RPF1 | 0.5117316 | 4.64760987 | 5.26995711 | 1.8716E-07 | 5.5459E-07 | 5.59182431 |
| Colon | ENSG00000198373.12 | WWP2 | 0.49922738 | 4.38944014 | 5.047515 | 5.852E-07 | 1.6843E-06 | 4.48996584 |
| Colon | ENSG00000108854.15 | SMURF2 | 0.4324045 | 2.82127579 | 4.85219577 | 1.5379E-06 | 4.3177E-06 | 3.55865751 |
| Colon | ENSG00000002746.14 | HECW1 | 0.5117966 | -2.2227252 | 3.80705613 | 0.00015425 | 0.00037454 | -0.8369626 |
| Colon | ENSG00000198742.9 | SMURF1 | 0.3279801 | 3.38592539 | 3.59172822 | 0.00035387 | 0.00083168 | -1.617096 |
| Colon | ENSG00000049759.16 | NEDD4L | -0.2602456 | 4.43029983 | -2.3659528 | 0.01828283 | 0.03479536 | -5.2178202 |
| Endometrium | ENSG00000049759.16 | NEDD4L | 1.39029603 | 4.23954002 | 6.96801654 | 4.4758E-11 | 1.3157E-09 | 14.6269394 |
| Endometrium | ENSG00000198373.12 | WWP2 | -0.6847005 | 3.87469383 | -4.3030966 | 2.6262E-05 | 0.00024644 | 1.78103296 |
| Endometrium | ENSG00000138411.10 | HECW2 | -0.8810445 | -1.4027739 | -3.0695637 | 0.00243903 | 0.0133354 | -2.4594947 |
| Endometrium | ENSG00000123124.13 | WWP1 | -0.6422536 | 3.32955314 | -2.5731486 | 0.01079649 | 0.04727947 | -3.8027939 |
| Endometrium | ENSG00000002746.14 | HECW1 | 1.19239845 | -2.1976096 | 2.13066596 | 0.03432915 | 0.12413156 | -4.8127744 |
| Endometrium | ENSG00000108854.15 | SMURF2 | -0.240704 | 2.76869835 | -1.100668 | 0.2723546 | 0.59457666 | -6.4493829 |
| Endometrium | ENSG00000198742.9 | SMURF1 | -0.1624913 | 2.68814083 | -0.8590734 | 0.39132109 | 0.73462866 | -6.6843961 |
| Endometrium | ENSG00000078747.12 | ITCH | -0.1040099 | 3.09310059 | -0.5154576 | 0.60679849 | 0.9045328 | -6.9193903 |
| Endometrium | ENSG00000117133.10 | RPF1 | -0.0668377 | 4.77793073 | -0.4706563 | 0.63839541 | 0.92206574 | -6.9413996 |
| Esophagus | ENSG00000002746.14 | HECW1 | 3.13730068 | -2.8715524 | 16.8945238 | 2.1568E-55 | 3.026E-54 | 114.919302 |
| Esophagus | ENSG00000049759.16 | NEDD4L | 1.55334289 | 3.78328442 | 14.2836194 | 1.2733E-41 | 1.1323E-40 | 83.3430652 |
| Esophagus | ENSG00000138411.10 | HECW2 | 1.29914848 | -0.0042176 | 11.9366544 | 1.8304E-30 | 1.0914E-29 | 57.7997877 |
| Esophagus | ENSG00000198742.9 | SMURF1 | 1.0243556 | 3.82371464 | 11.149581 | 4.9637E-27 | 2.6047E-26 | 49.952257 |
| Esophagus | ENSG00000108854.15 | SMURF2 | 0.90588696 | 3.01878973 | 9.57061824 | 1.1324E-20 | 4.6202E-20 | 35.4441337 |
| Esophagus | ENSG00000078747.12 | ITCH | 0.83518726 | 3.90605195 | 8.75630514 | 1.0857E-17 | 3.8951E-17 | 28.6568949 |
| Esophagus | ENSG00000123124.13 | WWP1 | 0.67924013 | 4.13875124 | 7.39748159 | 3.3464E-13 | 9.7685E-13 | 18.4716742 |
| Esophagus | ENSG00000198373.12 | WWP2 | 0.57888023 | 4.33588182 | 6.47730642 | 1.5829E-10 | 4.0486E-10 | 12.4324869 |
| Esophagus | ENSG00000117133.10 | RPF1 | 0.39477741 | 4.49346482 | 4.36916289 | 1.4022E-05 | 2.6575E-05 | 1.39574908 |
| Head And Neck Region | ENSG00000108854.15 | SMURF2 | 1.03055739 | 2.79439591 | 7.71908982 | 5.4096E-14 | 1.0145E-12 | 21.0173392 |
| Head And Neck Region | ENSG00000117133.10 | RPF1 | 0.44882964 | 4.84673541 | 6.75056569 | 3.6869E-11 | 4.8489E-10 | 14.6398611 |
| Head And Neck Region | ENSG00000198742.9 | SMURF1 | 0.78629275 | 3.77508701 | 5.97311277 | 4.1378E-09 | 4.1625E-08 | 10.0482741 |
| Head And Neck Region | ENSG00000002746.14 | HECW1 | 1.50709389 | -0.7994856 | 4.72604955 | 2.8987E-06 | 1.9144E-05 | 3.73214902 |
| Head And Neck Region | ENSG00000049759.16 | NEDD4L | 0.71460489 | 4.18440036 | 4.62064623 | 4.751E-06 | 3.0454E-05 | 3.26003313 |
| Head And Neck Region | ENSG00000138411.10 | HECW2 | -0.3837159 | -0.0630263 | -2.0035266 | 0.04560168 | 0.12607671 | -5.1820968 |
| Head And Neck Region | ENSG00000123124.13 | WWP1 | 0.13138325 | 4.19114929 | 1.03056958 | 0.30318723 | 0.562852 | -6.6455816 |
| Head And Neck Region | ENSG00000198373.12 | WWP2 | 0.08147747 | 4.07707117 | 0.91827399 | 0.35887044 | 0.62792556 | -6.7543832 |
| Head And Neck Region | ENSG00000078747.12 | ITCH | 0.12082533 | 3.88171797 | 0.88498368 | 0.37654494 | 0.65139596 | -6.7842357 |
| Kidney | ENSG00000002746.14 | HECW1 | -3.085131 | -2.204381 | -11.525492 | 3.326E-29 | 7.8112E-28 | 55.1215857 |
| Kidney | ENSG00000049759.16 | NEDD4L | -0.745291 | 4.98519018 | -8.4528152 | 8.2569E-17 | 9.3405E-16 | 26.8919808 |
| Kidney | ENSG00000123124.13 | WWP1 | -0.512939 | 4.23567609 | -5.870448 | 5.6383E-09 | 3.3907E-08 | 9.20666642 |
| Kidney | ENSG00000198373.12 | WWP2 | -0.2993826 | 4.55741929 | -4.1256639 | 3.9553E-05 | 0.00014998 | 0.6799071 |
| Kidney | ENSG00000198742.9 | SMURF1 | 0.28156668 | 3.10256444 | 3.85080755 | 0.00012403 | 0.00043512 | -0.4006971 |
| Kidney | ENSG00000117133.10 | RPF1 | -0.1188187 | 4.31217943 | -1.8094959 | 0.07062761 | 0.13519471 | -6.128695 |
| Kidney | ENSG00000138411.10 | HECW2 | 0.34633142 | 0.54579079 | 1.71599305 | 0.08642502 | 0.16027652 | -6.2929308 |
| Kidney | ENSG00000078747.12 | ITCH | -0.1076479 | 3.49017009 | -1.4047587 | 0.16035543 | 0.26936552 | -6.7771131 |
| Kidney | ENSG00000108854.15 | SMURF2 | 0.0870009 | 2.95671481 | 1.0081134 | 0.31360608 | 0.46893011 | -7.2545903 |
| Liver | ENSG00000049759.16 | NEDD4L | 1.00672329 | 4.36103856 | 11.8710617 | 5.6898E-29 | 9.2112E-28 | 54.7261737 |
| Liver | ENSG00000117133.10 | RPF1 | 0.55962511 | 3.78091607 | 9.98134198 | 1.3175E-21 | 1.4776E-20 | 37.917078 |
| Liver | ENSG00000198373.12 | WWP2 | 0.54634194 | 3.19249924 | 8.95757969 | 5.7036E-18 | 5.2013E-17 | 29.638082 |
| Liver | ENSG00000123124.13 | WWP1 | 0.68252825 | 4.58385936 | 8.25809256 | 1.1985E-15 | 9.5518E-15 | 24.3617691 |
| Liver | ENSG00000198742.9 | SMURF1 | 0.55205717 | 2.32266711 | 6.8363679 | 2.2526E-11 | 1.3472E-10 | 14.6882289 |
| Liver | ENSG00000002746.14 | HECW1 | 1.84105685 | -4.9472433 | 6.62285788 | 8.6993E-11 | 4.98E-10 | 13.3654339 |
| Liver | ENSG00000108854.15 | SMURF2 | 0.51693353 | 0.84966125 | 5.3443395 | 1.3528E-07 | 5.943E-07 | 6.20827865 |
| Liver | ENSG00000138411.10 | HECW2 | 0.33534228 | -0.807807 | 2.84386693 | 0.00462993 | 0.01183211 | -3.6843572 |
| Liver | ENSG00000078747.12 | ITCH | -0.1014022 | 3.06275577 | -1.4218718 | 0.15565452 | 0.27739675 | -6.6880797 |
| Lung | ENSG00000138411.10 | HECW2 | -2.1467112 | 1.42618426 | -31.654981 | 1.693E-166 | 8.986E-165 | 369.949409 |
| Lung | ENSG00000002746.14 | HECW1 | 2.33337832 | -1.5584594 | 19.5001609 | 3.7752E-75 | 4.2405E-74 | 159.979557 |
| Lung | ENSG00000049759.16 | NEDD4L | -0.8168495 | 5.35968918 | -14.286615 | 2.4435E-43 | 1.5091E-42 | 86.9887776 |
| Lung | ENSG00000198373.12 | WWP2 | -0.6058772 | 4.45859964 | -14.24445 | 4.1432E-43 | 2.5481E-42 | 86.4632846 |
| Lung | ENSG00000117133.10 | RPF1 | 0.24067326 | 4.64929987 | 6.4625203 | 1.416E-10 | 3.8023E-10 | 12.2661555 |
| Lung | ENSG00000078747.12 | ITCH | 0.23682067 | 3.89440132 | 5.72510239 | 1.2618E-08 | 3.1391E-08 | 7.88799128 |
| Lung | ENSG00000108854.15 | SMURF2 | -0.2727636 | 3.81215927 | -5.4377892 | 6.353E-08 | 1.5318E-07 | 6.31896763 |
| Lung | ENSG00000198742.9 | SMURF1 | -0.0834726 | 4.049952 | -1.7679292 | 0.07728951 | 0.12001759 | -6.7529953 |
| Lung | ENSG00000123124.13 | WWP1 | 0.01486153 | 4.25019208 | 0.3363188 | 0.73668064 | 0.87636274 | -8.2576782 |
| Ovary | ENSG00000198742.9 | SMURF1 | -1.235905 | 3.27217357 | -11.313401 | 1.3159E-26 | 8.8281E-26 | 48.9852716 |
| Ovary | ENSG00000198373.12 | WWP2 | -1.0121868 | 3.91957692 | -8.9924959 | 4.8528E-18 | 2.315E-17 | 29.4281469 |
| Ovary | ENSG00000123124.13 | WWP1 | -0.8911042 | 3.23988067 | -8.0069524 | 8.1425E-15 | 3.3756E-14 | 22.0942389 |
| Ovary | ENSG00000049759.16 | NEDD4L | 0.89813195 | 3.39838067 | 7.48931596 | 3.1087E-13 | 1.1982E-12 | 18.5060734 |
| Ovary | ENSG00000117133.10 | RPF1 | 0.4411211 | 4.69511479 | 4.49136918 | 8.7765E-06 | 2.232E-05 | 1.7836281 |
| Ovary | ENSG00000078747.12 | ITCH | -0.3932079 | 3.31460828 | -3.8004035 | 0.00016207 | 0.00037278 | -0.9892002 |
| Ovary | ENSG00000108854.15 | SMURF2 | -0.2968522 | 2.95430316 | -2.7473086 | 0.0062232 | 0.01222612 | -4.3671939 |
| Ovary | ENSG00000002746.14 | HECW1 | 0.63823936 | -1.2727308 | 2.7451208 | 0.00626422 | 0.0123031 | -4.3731201 |
| Ovary | ENSG00000138411.10 | HECW2 | 0.20104929 | -1.134573 | 1.42034919 | 0.15612255 | 0.24363164 | -7.1094618 |
| Pancreas | ENSG00000002746.14 | HECW1 | 5.89261983 | -4.2006564 | 24.4328184 | 1.9419E-77 | 1.2646E-75 | 165.896709 |
| Pancreas | ENSG00000138411.10 | HECW2 | 2.59152092 | -0.6111963 | 20.3622947 | 3.1992E-61 | 8.7064E-60 | 128.634294 |
| Pancreas | ENSG00000117133.10 | RPF1 | 1.70377828 | 3.8489851 | 14.3223082 | 6.8995E-37 | 5.9434E-36 | 72.7595412 |
| Pancreas | ENSG00000108854.15 | SMURF2 | 1.65071181 | 2.3363957 | 14.0155912 | 1.0986E-35 | 9.0192E-35 | 70.002673 |
| Pancreas | ENSG00000198742.9 | SMURF1 | 1.76073601 | 3.26053868 | 13.4288701 | 2.0897E-33 | 1.5636E-32 | 64.7766295 |
| Pancreas | ENSG00000078747.12 | ITCH | 1.51732834 | 2.80370315 | 12.9282871 | 1.7415E-31 | 1.2092E-30 | 60.3740001 |
| Pancreas | ENSG00000123124.13 | WWP1 | 1.61675556 | 3.5555788 | 12.8748964 | 2.7822E-31 | 1.9148E-30 | 59.9077936 |
| Pancreas | ENSG00000198373.12 | WWP2 | 0.92276467 | 3.96769198 | 7.63434834 | 2.215E-13 | 7.9485E-13 | 19.0319956 |
| Pancreas | ENSG00000049759.16 | NEDD4L | -0.2733078 | 4.31148825 | -1.9840568 | 0.04803606 | 0.08839805 | -5.989869 |
| Prostate | ENSG00000049759.16 | NEDD4L | 1.08999943 | 6.90462998 | 12.9340916 | 3.4369E-34 | 4.5104E-33 | 66.6610345 |
| Prostate | ENSG00000198742.9 | SMURF1 | -0.661528 | 2.99418311 | -10.749434 | 6.614E-25 | 5.5947E-24 | 45.4583044 |
| Prostate | ENSG00000002746.14 | HECW1 | -1.3639903 | -0.9660889 | -8.3444773 | 4.3437E-16 | 2.3585E-15 | 25.3930367 |
| Prostate | ENSG00000117133.10 | RPF1 | 0.23881105 | 4.70764851 | 6.42983373 | 2.4859E-10 | 9.8397E-10 | 12.3789304 |
| Prostate | ENSG00000123124.13 | WWP1 | 0.4004066 | 4.70073495 | 5.6708567 | 2.1447E-08 | 7.5374E-08 | 8.03782385 |
| Prostate | ENSG00000138411.10 | HECW2 | -0.4775407 | -1.4027433 | -4.1764068 | 3.3687E-05 | 9.4145E-05 | 0.95734433 |
| Prostate | ENSG00000078747.12 | ITCH | -0.156053 | 3.43930924 | -2.3091751 | 0.02124874 | 0.04351601 | -4.9884162 |
| Prostate | ENSG00000198373.12 | WWP2 | 0.04953865 | 4.59652288 | 0.94721141 | 0.34388554 | 0.51832151 | -7.1937188 |
| Prostate | ENSG00000108854.15 | SMURF2 | -0.0353226 | 3.30351851 | -0.5498225 | 0.58263132 | 0.77208573 | -7.4906826 |
| Rectum | ENSG00000049759.16 | NEDD4L | -1.3301243 | 4.3155 | -6.3139154 | 7.4739E-09 | 3.8979E-07 | 9.88407197 |
| Rectum | ENSG00000138411.10 | HECW2 | 1.21151652 | -0.4961696 | 3.17467372 | 0.00199222 | 0.01441583 | -2.0512568 |
| Rectum | ENSG00000198742.9 | SMURF1 | 0.51582087 | 3.5718402 | 2.29997594 | 0.02351992 | 0.10888493 | -4.2709671 |
| Rectum | ENSG00000198373.12 | WWP2 | -0.2468585 | 4.65218333 | -1.4783874 | 0.14243563 | 0.43117691 | -5.7649636 |
| Rectum | ENSG00000108854.15 | SMURF2 | 0.20630587 | 3.0280098 | 0.92298698 | 0.35822755 | 0.80463543 | -6.4209506 |
| Rectum | ENSG00000123124.13 | WWP1 | 0.16169022 | 4.56448824 | 0.65745026 | 0.51239639 | 0.98064554 | -6.6291697 |
| Rectum | ENSG00000078747.12 | ITCH | 0.1058813 | 4.13171078 | 0.50008309 | 0.61811127 | 1 | -6.7198215 |
| Rectum | ENSG00000117133.10 | RPF1 | 0.01156435 | 4.96127059 | 0.07605904 | 0.93952354 | 1 | -6.8416585 |
| Rectum | ENSG00000002746.14 | HECW1 | 0.02035717 | -1.9461186 | 0.03349406 | 0.97334712 | 1 | -6.8439871 |
| Skin | ENSG00000049759.16 | NEDD4L | 1.58132723 | 4.13172564 | 17.5331163 | 9.4162E-57 | 7.6826E-56 | 117.849042 |
| Skin | ENSG00000123124.13 | WWP1 | -0.9898689 | 4.27445508 | -15.529234 | 1.4672E-46 | 9.995E-46 | 94.4466691 |
| Skin | ENSG00000198742.9 | SMURF1 | -0.6525232 | 4.0254953 | -13.255907 | 1.0289E-35 | 5.7054E-35 | 69.5717544 |
| Skin | ENSG00000138411.10 | HECW2 | -1.3555809 | -0.2517672 | -12.892286 | 4.5749E-34 | 2.4504E-33 | 65.7956235 |
| Skin | ENSG00000108854.15 | SMURF2 | -0.8977012 | 2.9637041 | -12.337847 | 1.3211E-31 | 6.7329E-31 | 60.1599866 |
| Skin | ENSG00000078747.12 | ITCH | -0.5320758 | 3.41020744 | -8.3405305 | 4.3412E-16 | 1.5243E-15 | 24.7300445 |
| Skin | ENSG00000198373.12 | WWP2 | -0.4398626 | 4.75274052 | -8.1281112 | 2.168E-15 | 7.4651E-15 | 23.1429874 |
| Skin | ENSG00000002746.14 | HECW1 | -1.7094649 | -2.4862505 | -7.9658506 | 7.2444E-15 | 2.4546E-14 | 21.9532514 |
| Skin | ENSG00000117133.10 | RPF1 | 0.20609136 | 4.48266039 | 4.19960158 | 3.0426E-05 | 7.0012E-05 | 0.34660504 |
| Stomach | ENSG00000138411.10 | HECW2 | 2.12868214 | 0.0920154 | 21.4326496 | 9.5327E-77 | 5.4561E-75 | 164.090596 |
| Stomach | ENSG00000078747.12 | ITCH | 1.31955905 | 3.70670239 | 14.7331176 | 2.2918E-42 | 3.0247E-41 | 85.2049008 |
| Stomach | ENSG00000117133.10 | RPF1 | 0.88958264 | 4.4735072 | 11.2576017 | 6.8921E-27 | 4.802E-26 | 49.7770117 |
| Stomach | ENSG00000123124.13 | WWP1 | 0.92310251 | 4.11611444 | 10.5897976 | 3.2994E-24 | 2.0414E-23 | 43.6560567 |
| Stomach | ENSG00000108854.15 | SMURF2 | 0.82034 | 2.97990737 | 10.0828764 | 3.0085E-22 | 1.7036E-21 | 39.1839512 |
| Stomach | ENSG00000198742.9 | SMURF1 | 0.77893883 | 3.66621691 | 8.49922595 | 1.4115E-16 | 6.0934E-16 | 26.2703351 |
| Stomach | ENSG00000198373.12 | WWP2 | 0.65275681 | 4.46477047 | 7.87792335 | 1.4892E-14 | 5.8009E-14 | 21.6773786 |
| Stomach | ENSG00000002746.14 | HECW1 | 1.0887995 | -1.7459953 | 7.17563442 | 2.0542E-12 | 7.1464E-12 | 16.8323294 |
| Stomach | ENSG00000049759.16 | NEDD4L | 0.07926295 | 4.72493268 | 0.77008038 | 0.44154433 | 0.54456504 | -7.6122895 |
| Testis | ENSG00000198742.9 | SMURF1 | -1.706273 | 3.91064665 | -24.59279 | 5.7968E-75 | 4.2904E-74 | 160.024356 |
| Testis | ENSG00000002746.14 | HECW1 | -4.1645553 | 0.54460096 | -22.607955 | 1.1793E-67 | 7.3824E-67 | 143.194688 |
| Testis | ENSG00000078747.12 | ITCH | -1.4257789 | 4.30348435 | -20.701774 | 1.7389E-60 | 9.3347E-60 | 126.69009 |
| Testis | ENSG00000108854.15 | SMURF2 | -0.7783654 | 3.66454473 | -14.175039 | 1.5362E-35 | 5.0865E-35 | 69.3129572 |
| Testis | ENSG00000198373.12 | WWP2 | -0.6910069 | 4.41883099 | -12.90524 | 8.1315E-31 | 2.4749E-30 | 58.4678622 |
| Testis | ENSG00000138411.10 | HECW2 | -1.1143679 | 0.7649754 | -11.310263 | 4.4343E-25 | 1.2136E-24 | 45.3139792 |
| Testis | ENSG00000117133.10 | RPF1 | -0.1513615 | 4.94417636 | -3.6534832 | 0.00030345 | 0.00048611 | -1.8618326 |
| Testis | ENSG00000123124.13 | WWP1 | 0.01725161 | 4.16831246 | 0.26728076 | 0.78942988 | 0.93339318 | -8.3787098 |
| Testis | ENSG00000049759.16 | NEDD4L | 0.00925567 | 3.52335527 | 0.08356664 | 0.93345467 | 1 | -8.4110214 |
| Thyroid Gland | ENSG00000002746.14 | HECW1 | -3.9993895 | -3.4389332 | -10.166241 | 2.1245E-22 | 1.0458E-20 | 40.0277211 |
| Thyroid Gland | ENSG00000123124.13 | WWP1 | -0.7637216 | 3.66299112 | -7.8397566 | 2.2815E-14 | 3.9686E-13 | 21.8351995 |
| Thyroid Gland | ENSG00000117133.10 | RPF1 | -0.284385 | 4.82235293 | -5.3999806 | 9.8535E-08 | 6.9284E-07 | 6.94574244 |
| Thyroid Gland | ENSG00000078747.12 | ITCH | -0.3920396 | 3.45430551 | -4.0970818 | 4.8004E-05 | 0.00021644 | 1.02405956 |
| Thyroid Gland | ENSG00000049759.16 | NEDD4L | -0.2277629 | 4.67551243 | -3.2953341 | 0.00104506 | 0.00369386 | -1.8576363 |
| Thyroid Gland | ENSG00000198373.12 | WWP2 | -0.1624996 | 4.46031954 | -2.5036174 | 0.01257582 | 0.03481397 | -4.1105886 |
| Thyroid Gland | ENSG00000198742.9 | SMURF1 | -0.0452282 | 2.29652682 | -0.5247505 | 0.59996381 | 0.89096254 | -7.0790565 |
| Thyroid Gland | ENSG00000108854.15 | SMURF2 | 0.02267342 | 3.42311599 | 0.22138192 | 0.82487557 | 1 | -7.1918023 |
| Thyroid Gland | ENSG00000138411.10 | HECW2 | 0.03351719 | 0.79009964 | 0.21455849 | 0.83018947 | 1 | -7.1932843 |
| Uterus | ENSG00000049759.16 | NEDD4L | 1.52797699 | 3.55005481 | 15.2271612 | 5.7172E-31 | 1.2964E-29 | 59.6926945 |
| Uterus | ENSG00000198373.12 | WWP2 | -1.4216113 | 4.47951926 | -12.936391 | 2.5992E-25 | 3.6843E-24 | 46.6851458 |
| Uterus | ENSG00000198742.9 | SMURF1 | -1.1102463 | 3.34077037 | -12.042773 | 4.5999E-23 | 5.4491E-22 | 41.5209329 |
| Uterus | ENSG00000123124.13 | WWP1 | -0.9501909 | 3.69570444 | -7.4783525 | 8.995E-12 | 4.5706E-11 | 15.6751327 |
| Uterus | ENSG00000108854.15 | SMURF2 | -0.61437 | 3.16000444 | -6.4189722 | 2.2145E-09 | 9.4541E-09 | 10.2471028 |
| Uterus | ENSG00000078747.12 | ITCH | -0.5977872 | 3.58430222 | -6.343576 | 3.229E-09 | 1.3627E-08 | 9.87652173 |
| Uterus | ENSG00000002746.14 | HECW1 | 2.59483387 | -3.465483 | 6.0474255 | 1.3899E-08 | 5.5856E-08 | 8.44414103 |
| Uterus | ENSG00000117133.10 | RPF1 | 0.23024831 | 4.97528519 | 4.2097066 | 4.6695E-05 | 0.00013908 | 0.56051762 |
| Uterus | ENSG00000138411.10 | HECW2 | -0.102275 | -0.3588148 | -0.551536 | 0.582189 | 0.81231269 | -7.6542385 |

Supplementary Table 2. The relationship between methylation of NEDD4 family members and tumor prognosis.

| **Cancer** | **Gene** | **HR** | **low95** | **up95** | **p.val** |
| --- | --- | --- | --- | --- | --- |
| ACC | HECW1 | 0.288127914 | 0.136637402 | 0.607576651 | 0.000895629 |
| ACC | HECW2 | 0.279339089 | 0.081287969 | 0.959924666 | 0.000946731 |
| ACC | ITCH | 1.755487985 | 0.829061563 | 3.717140202 | 0.124340606 |
| ACC | NEDD4L | 0.22729598 | 0.08333991 | 0.619912624 | 1.05499E-05 |
| ACC | RPF1 | 0.462515298 | 0.155760008 | 1.373397463 | 0.06768429 |
| ACC | SMURF1 | 0.598711114 | 0.28841436 | 1.242847261 | 0.164047136 |
| ACC | SMURF2 | 3.335957082 | 1.480417138 | 7.517212123 | 0.034904022 |
| ACC | WWP1 | 2.212247499 | 0.604299421 | 8.09869881 | 0.094852811 |
| ACC | WWP2 | 0.237416469 | 0.08407998 | 0.670392398 | 2.89159E-05 |
| BLCA | HECW1 | 0.665085493 | 0.450888528 | 0.981037852 | 0.018328149 |
| BLCA | HECW2 | 0.505014768 | 0.309385299 | 0.824344001 | 0.000303261 |
| BLCA | ITCH | 0.511290155 | 0.333131182 | 0.784728771 | 0.01700321 |
| BLCA | NEDD4L | 0.651703749 | 0.487708661 | 0.870843212 | 0.006425969 |
| BLCA | RPF1 | 0.419453525 | 0.249086397 | 0.70634632 | 2.34345E-06 |
| BLCA | SMURF1 | 1.195334078 | 0.891243298 | 1.603180144 | 0.222099944 |
| BLCA | SMURF2 | 0.552529288 | 0.415932753 | 0.733985509 | 0.000113782 |
| BLCA | WWP1 | 0.564214811 | 0.417316262 | 0.762822785 | 0.000954103 |
| BLCA | WWP2 | 3.000924825 | 2.029049286 | 4.438310036 | 0.000189508 |
| BRCA | HECW1 | 0.599575402 | 0.422851681 | 0.850157819 | 0.002177788 |
| BRCA | HECW2 | 0.631533735 | 0.37228499 | 1.071315978 | 0.041124835 |
| BRCA | ITCH | 1.76225953 | 1.155572142 | 2.687464103 | 0.001883622 |
| BRCA | NEDD4L | 0.753475154 | 0.524652571 | 1.082096686 | 0.147841375 |
| BRCA | RPF1 | 1.453222867 | 0.995076888 | 2.122305045 | 0.077271652 |
| BRCA | SMURF1 | 1.870717368 | 1.31930794 | 2.652590321 | 0.000146547 |
| BRCA | SMURF2 | 0.767752164 | 0.511195308 | 1.153068849 | 0.166453309 |
| BRCA | WWP1 | 0.765366629 | 0.472562777 | 1.2395942 | 0.228827929 |
| BRCA | WWP2 | 1.974049221 | 1.323081414 | 2.945298971 | 7.84546E-05 |
| CESC | HECW1 | 1.744143733 | 1.030731662 | 2.951337844 | 0.073224455 |
| CESC | HECW2 | 2.741023077 | 1.40693809 | 5.34011238 | 0.040607197 |
| CESC | ITCH | 2.48534984 | 1.371556453 | 4.503616174 | 0.026016504 |
| CESC | NEDD4L | 2.144115917 | 1.309716888 | 3.510096805 | 0.010254496 |
| CESC | RPF1 | 0.445297843 | 0.223690894 | 0.886447211 | 0.002170469 |
| CESC | SMURF1 | 2.435023568 | 1.412474443 | 4.197838629 | 0.013581665 |
| CESC | SMURF2 | 1.968574536 | 1.176109279 | 3.295004786 | 0.003425965 |
| CESC | WWP1 | 0.528353164 | 0.294051909 | 0.94934621 | 0.081614858 |
| CESC | WWP2 | 2.334548287 | 1.426180701 | 3.821476268 | 0.000168289 |
| CHOL | HECW1 | 2.221439002 | 0.519339237 | 9.5020574 | 0.126174293 |
| CHOL | HECW2 | 1.784229848 | 0.706681673 | 4.504823424 | 0.150755925 |
| CHOL | ITCH | 5.280545225 | 0.436196958 | 63.9256129 | 0.001983708 |
| CHOL | NEDD4L | 2.050084616 | 0.700765469 | 5.997508609 | 0.097343529 |
| CHOL | RPF1 | 2.186725346 | 0.600550981 | 7.962301105 | 0.106086311 |
| CHOL | SMURF1 | 2.8775905 | 0.776381931 | 10.66553297 | 0.015639978 |
| CHOL | SMURF2 | 0.495947211 | 0.181571738 | 1.354636134 | 0.087305391 |
| CHOL | WWP1 | 6.773334132 | 0.828746668 | 55.35835864 | 4.53755E-06 |
| CHOL | WWP2 | 2.604402921 | 0.812676338 | 8.346391131 | 0.024427542 |
| COAD | HECW1 | 0.663449139 | 0.394958692 | 1.114457712 | 0.084963526 |
| COAD | HECW2 | 0.599676144 | 0.378446294 | 0.950231206 | 0.047974042 |
| COAD | ITCH | 0.592850678 | 0.315061143 | 1.115567356 | 0.176505927 |
| COAD | NEDD4L | 0.549571292 | 0.335346101 | 0.900647433 | 0.007318706 |
| COAD | RPF1 | 0.719887374 | 0.46546196 | 1.113383854 | 0.144062297 |
| COAD | SMURF1 | 0.59193699 | 0.313513848 | 1.117620172 | 0.050706451 |
| COAD | SMURF2 | 0.732647165 | 0.473615849 | 1.13334862 | 0.167460444 |
| COAD | WWP1 | 0.568442246 | 0.344153557 | 0.938902358 | 0.05649674 |
| COAD | WWP2 | 1.668924612 | 0.93792707 | 2.969643857 | 0.138584853 |
| DLBC | HECW1 | 3.606027463 | 0.809211061 | 16.06927375 | 0.035900386 |
| DLBC | HECW2 | 0.318590247 | 0.074564797 | 1.361228749 | 0.158999739 |
| DLBC | ITCH | 7.051803428 | 1.904158779 | 26.1154333 | 0.014645106 |
| DLBC | NEDD4L | 3.347275023 | 0.295553171 | 37.90942261 | 0.101670905 |
| DLBC | RPF1 | 4.017747551 | 1.003798829 | 16.08120564 | 0.021385918 |
| DLBC | SMURF1 | 3.123292681 | 0.729842682 | 13.36583542 | 0.160633603 |
| DLBC | SMURF2 | 3.175231567 | 0.472248205 | 21.34914521 | 0.071614505 |
| DLBC | WWP1 | #NUM! | #NUM! | #NUM! | 0.180949562 |
| DLBC | WWP2 | 3.803287975 | 0.836712389 | 17.28789918 | 0.026473198 |
| ESCA | HECW1 | 0.794138711 | 0.498769158 | 1.264425201 | 0.302979938 |
| ESCA | HECW2 | 1.847238203 | 1.059545074 | 3.220522716 | 0.066386022 |
| ESCA | ITCH | 1.37422659 | 0.865702079 | 2.181464924 | 0.203938989 |
| ESCA | NEDD4L | 0.710809536 | 0.386573913 | 1.306995063 | 0.321221209 |
| ESCA | RPF1 | 1.62739857 | 0.779907806 | 3.39581946 | 0.28424995 |
| ESCA | SMURF1 | 1.502661856 | 0.932559347 | 2.421285746 | 0.068836979 |
| ESCA | SMURF2 | 1.560512372 | 0.983372383 | 2.47637508 | 0.042063543 |
| ESCA | WWP1 | 1.50415151 | 0.863106373 | 2.621312778 | 0.099922221 |
| ESCA | WWP2 | 0.512000256 | 0.27122204 | 0.966530087 | 0.104047306 |
| GBM | HECW1 | 1.945260167 | 1.09325941 | 3.461243585 | 0.003324113 |
| GBM | HECW2 | 1.722409096 | 1.032962561 | 2.872023832 | 0.080208624 |
| GBM | ITCH | 1.458188301 | 0.988231447 | 2.151634749 | 0.065085818 |
| GBM | NEDD4L | 1.933450078 | 1.312854719 | 2.847405085 | 0.001843363 |
| GBM | RPF1 | 2.014405953 | 1.344708345 | 3.017629332 | 0.002690277 |
| GBM | SMURF1 | 1.950720602 | 1.149976149 | 3.309034601 | 0.047761364 |
| GBM | SMURF2 | 2.6530811 | 1.612953816 | 4.363943501 | 0.003539171 |
| GBM | WWP1 | 1.406998493 | 0.919035812 | 2.154045285 | 0.088178341 |
| GBM | WWP2 | 0.588123891 | 0.27866674 | 1.241230692 | 0.076721132 |
| HNSC | HECW1 | 0.752570975 | 0.588926664 | 0.961686925 | 0.022143043 |
| HNSC | HECW2 | 0.763877568 | 0.56376982 | 1.035012726 | 0.108037112 |
| HNSC | ITCH | 1.551911529 | 1.057245947 | 2.278021872 | 0.007615211 |
| HNSC | NEDD4L | 1.187407143 | 0.89284801 | 1.579144163 | 0.215302892 |
| HNSC | RPF1 | 1.262032821 | 0.933027921 | 1.707051637 | 0.154837568 |
| HNSC | SMURF1 | 1.753767965 | 1.246058274 | 2.46834529 | 9.7433E-05 |
| HNSC | SMURF2 | 1.496522931 | 1.063470213 | 2.105917829 | 0.007726371 |
| HNSC | WWP1 | 0.745402947 | 0.556433632 | 0.998547753 | 0.069383453 |
| HNSC | WWP2 | 1.320402215 | 1.033399944 | 1.687112546 | 0.026477762 |
| KICH | HECW1 | 7.949090816 | 0.823986896 | 76.68573993 | 0.00023342 |
| KICH | HECW2 | 5.885070315 | 0.517160095 | 66.9696927 | 0.004341836 |
| KICH | ITCH | 0.331922064 | 0.089531042 | 1.230548137 | 0.147979861 |
| KICH | NEDD4L | 1.921911715 | 0.458034057 | 8.064344962 | 0.321425152 |
| KICH | RPF1 | 0.375947737 | 0.079827475 | 1.770527022 | 0.129077354 |
| KICH | SMURF1 | 6.222650948 | 1.080808477 | 35.82631488 | 0.001756022 |
| KICH | SMURF2 | 3.521794113 | 0.294980183 | 42.04700678 | 0.093166907 |
| KICH | WWP1 | 3.068751789 | 0.806200303 | 11.68101465 | 0.094414884 |
| KICH | WWP2 | 2.720157162 | 0.57217342 | 12.93183975 | 0.119381002 |
| KIRC | HECW1 | 0.545635514 | 0.407975131 | 0.729745741 | 0.000116758 |
| KIRC | HECW2 | 0.73838349 | 0.491442674 | 1.10940748 | 0.104122703 |
| KIRC | ITCH | 0.713333268 | 0.441473284 | 1.152605083 | 0.113616507 |
| KIRC | NEDD4L | 0.641487168 | 0.458622186 | 0.897265329 | 0.003990688 |
| KIRC | RPF1 | 1.534122338 | 1.005241276 | 2.341260158 | 0.088372055 |
| KIRC | SMURF1 | 0.491827036 | 0.299644972 | 0.807268122 | 0.000183044 |
| KIRC | SMURF2 | 1.538401264 | 1.117118382 | 2.118556534 | 0.004095538 |
| KIRC | WWP1 | 0.65340493 | 0.404528963 | 1.055395389 | 0.0390001 |
| KIRC | WWP2 | 1.354978762 | 1.008671267 | 1.820184142 | 0.038206037 |
| KIRP | HECW1 | 2.71039659 | 1.094248483 | 6.71351141 | 0.001584292 |
| KIRP | HECW2 | 1.946808945 | 1.094490979 | 3.462856379 | 0.045511531 |
| KIRP | ITCH | 0.576320027 | 0.317387857 | 1.0464949 | 0.047808456 |
| KIRP | NEDD4L | 0.241148191 | 0.092999825 | 0.625296342 | 4.56059E-07 |
| KIRP | RPF1 | 0.51347369 | 0.281566386 | 0.936387452 | 0.015378979 |
| KIRP | SMURF1 | 0.545377707 | 0.241414583 | 1.232058307 | 0.069869563 |
| KIRP | SMURF2 | 0.364420546 | 0.184251446 | 0.720766853 | 0.000206547 |
| KIRP | WWP1 | 0.573309128 | 0.330709552 | 0.993873184 | 0.0569939 |
| KIRP | WWP2 | 0.377193635 | 0.213102838 | 0.667635588 | 0.005659013 |
| LAML | HECW1 | 1.576222092 | 1.050705077 | 2.364579878 | 0.014924491 |
| LAML | HECW2 | 1.773448005 | 1.107591573 | 2.839600718 | 0.045487175 |
| LAML | ITCH | 1.540997792 | 0.943221694 | 2.517620419 | 0.044577488 |
| LAML | NEDD4L | 1.996597316 | 1.102546949 | 3.615629107 | 0.002514396 |
| LAML | RPF1 | 1.379758234 | 0.945228537 | 2.014044974 | 0.107270432 |
| LAML | SMURF1 | 1.451912272 | 1.002584725 | 2.102614566 | 0.039923252 |
| LAML | SMURF2 | 0.677824791 | 0.388856734 | 1.181531416 | 0.231571731 |
| LAML | WWP1 | 1.464710759 | 0.961913597 | 2.230322571 | 0.049110666 |
| LAML | WWP2 | 0.514473005 | 0.351121803 | 0.75381953 | 0.001987288 |
| LGG | HECW1 | 4.052929238 | 2.619233155 | 6.271391065 | 0 |
| LGG | HECW2 | 2.056133662 | 1.31871513 | 3.205912739 | 7.39003E-05 |
| LGG | ITCH | 3.172138822 | 1.951790764 | 5.155503804 | 1.86083E-11 |
| LGG | NEDD4L | 5.042149347 | 2.845292514 | 8.935204345 | 0 |
| LGG | RPF1 | 2.163063865 | 1.369733308 | 3.415880491 | 2.6443E-05 |
| LGG | SMURF1 | 2.512057554 | 1.693463649 | 3.72634698 | 2.77068E-08 |
| LGG | SMURF2 | 4.011840378 | 2.606798223 | 6.17418835 | 0 |
| LGG | WWP1 | 2.84345429 | 1.98611701 | 4.070874102 | 3.33581E-10 |
| LGG | WWP2 | 3.834002831 | 2.584589484 | 5.687393607 | 0 |
| LIHC | HECW1 | 0.812830585 | 0.591458456 | 1.117058272 | 0.187309948 |
| LIHC | HECW2 | 1.471365996 | 0.953688829 | 2.270046404 | 0.125548804 |
| LIHC | ITCH | 0.715705418 | 0.526305001 | 0.973265016 | 0.037901497 |
| LIHC | NEDD4L | 1.347359563 | 0.963710713 | 1.883737275 | 0.063586658 |
| LIHC | RPF1 | 1.773476634 | 1.293645459 | 2.431283895 | 0.001193459 |
| LIHC | SMURF1 | 0.564829004 | 0.362026826 | 0.881238019 | 0.001991664 |
| LIHC | SMURF2 | 0.278410358 | 0.188495245 | 0.411216355 | 2.33069E-05 |
| LIHC | WWP1 | 0.567833507 | 0.414624566 | 0.777655059 | 0.001245402 |
| LIHC | WWP2 | 1.278467187 | 0.832142377 | 1.96418112 | 0.220329349 |
| LUAD | HECW1 | 1.502762986 | 1.045261512 | 2.160508702 | 0.013532318 |
| LUAD | HECW2 | 1.351830411 | 0.98827779 | 1.849121249 | 0.074091068 |
| LUAD | ITCH | 1.469925183 | 0.930564314 | 2.321902969 | 0.054711502 |
| LUAD | NEDD4L | 0.749681751 | 0.558769815 | 1.005821562 | 0.056559265 |
| LUAD | RPF1 | 1.482737264 | 0.991191473 | 2.218047526 | 0.093838429 |
| LUAD | SMURF1 | 1.349922527 | 0.893457949 | 2.03959328 | 0.113487502 |
| LUAD | SMURF2 | 0.844498927 | 0.62947746 | 1.132968983 | 0.259341292 |
| LUAD | WWP1 | 0.717100115 | 0.532063849 | 0.96648659 | 0.024441255 |
| LUAD | WWP2 | 0.704188968 | 0.501383798 | 0.989026977 | 0.027257676 |
| MESO | HECW1 | 2.73084164 | 1.609515332 | 4.633379947 | 0.002753459 |
| MESO | HECW2 | 1.535333271 | 0.851923289 | 2.76697243 | 0.097078982 |
| MESO | ITCH | 1.365929962 | 0.82315135 | 2.266611917 | 0.18954976 |
| MESO | NEDD4L | 1.638047335 | 1.00645552 | 2.665988728 | 0.058276472 |
| MESO | RPF1 | 2.133995994 | 0.935014404 | 4.870447859 | 0.012865285 |
| MESO | SMURF1 | 1.814978525 | 1.06358968 | 3.097197266 | 0.04747754 |
| MESO | SMURF2 | 0.701501765 | 0.408694805 | 1.204088528 | 0.240153021 |
| MESO | WWP1 | 0.375939587 | 0.162855539 | 0.867827854 | 0.000496556 |
| MESO | WWP2 | 0.596117136 | 0.369026419 | 0.962954469 | 0.050250514 |
| OV | HECW1 | #NUM! | #NUM! | #NUM! | 0.192948092 |
| OV | HECW2 | #NUM! | #NUM! | #NUM! | 0.192948092 |
| OV | ITCH | 6.859133127 | 0.698603939 | 67.345322 | 0.010755986 |
| OV | NEDD4L | 6.859133127 | 0.698603939 | 67.345322 | 0.010755986 |
| OV | RPF1 | 0.277471807 | 0.040511537 | 1.900461185 | 0.108377832 |
| OV | SMURF1 | 8.743362832 | 1.32250813 | 57.80410108 | 0.011222304 |
| OV | SMURF2 | 4.035714286 | 0.699157607 | 23.2951621 | 0.169370846 |
| OV | WWP1 | 6.976645435 | 0.312265802 | 155.8722767 | 0.009951709 |
| OV | WWP2 | #NUM! | #NUM! | #NUM! | 0.124980849 |
| PAAD | HECW1 | 1.515148871 | 1.018266389 | 2.254494625 | 0.054762171 |
| PAAD | HECW2 | 0.782556229 | 0.47605216 | 1.286401581 | 0.294157743 |
| PAAD | ITCH | 1.899948003 | 1.252415371 | 2.882272525 | 0.000713377 |
| PAAD | NEDD4L | 1.487801765 | 1.004181438 | 2.204336794 | 0.061361986 |
| PAAD | RPF1 | 2.023427992 | 1.138857139 | 3.595060959 | 0.064632261 |
| PAAD | SMURF1 | 1.569318997 | 1.064830302 | 2.312821218 | 0.017819265 |
| PAAD | SMURF2 | 1.969215373 | 1.007191747 | 3.850120094 | 0.008537148 |
| PAAD | WWP1 | 0.610343234 | 0.369194594 | 1.009004112 | 0.101413578 |
| PAAD | WWP2 | 0.550386649 | 0.353644354 | 0.856582214 | 0.02248479 |
| PCPG | HECW1 | 4.351739898 | 0.29376298 | 64.46571359 | 0.047161265 |
| PCPG | HECW2 | 0.125138144 | 0.013810174 | 1.133914371 | 0.000416015 |
| PCPG | ITCH | 0.156244854 | 0.028215864 | 0.865203131 | 0.001636673 |
| PCPG | NEDD4L | 7.09373762 | 0.535264351 | 94.0117034 | 0.001572149 |
| PCPG | RPF1 | 0.117024006 | 0.029066229 | 0.471152206 | 0.015375038 |
| PCPG | SMURF1 | 0.15698598 | 0.028417013 | 0.867248 | 0.001701596 |
| PCPG | SMURF2 | 0.209814668 | 0.033649613 | 1.30825263 | 0.006891463 |
| PCPG | WWP1 | 0.232286477 | 0.056792718 | 0.950069117 | 0.048088624 |
| PCPG | WWP2 | 0.157870084 | 0.020955809 | 1.189310501 | 0.001693711 |
| PRAD | HECW1 | 0.418692836 | 0.074547818 | 2.351560334 | 0.188912778 |
| PRAD | HECW2 | 0.291492168 | 0.040543616 | 2.095710542 | 0.055466314 |
| PRAD | ITCH | #NUM! | #NUM! | #NUM! | 0.073051124 |
| PRAD | NEDD4L | 3.768828177 | 0.954385715 | 14.88294052 | 0.019502719 |
| PRAD | RPF1 | 0.24310305 | 0.040625791 | 1.454718563 | 0.016276248 |
| PRAD | SMURF1 | 4.68566105 | 0.500416036 | 43.87433237 | 0.012903139 |
| PRAD | SMURF2 | 0.417365102 | 0.074169703 | 2.348581999 | 0.188303879 |
| PRAD | WWP1 | 5.121738775 | 1.411052022 | 18.59053222 | 0.075119581 |
| PRAD | WWP2 | 0.248614683 | 0.042147297 | 1.466505937 | 0.017645246 |
| READ | HECW1 | 0.349919718 | 0.13496499 | 0.907226451 | 0.052679905 |
| READ | HECW2 | 0.430617366 | 0.161791104 | 1.146115648 | 0.075527568 |
| READ | ITCH | 0.427199933 | 0.160356791 | 1.138085777 | 0.073461945 |
| READ | NEDD4L | 2.787592017 | 0.850743929 | 9.133969683 | 0.028666894 |
| READ | RPF1 | 3.68228642 | 1.088280876 | 12.45931411 | 0.136349911 |
| READ | SMURF1 | 0.455650041 | 0.085789795 | 2.420065937 | 0.201627559 |
| READ | SMURF2 | 0.362607003 | 0.094724789 | 1.38806156 | 0.302065948 |
| READ | WWP1 | 0.414999451 | 0.149570659 | 1.151459417 | 0.053716749 |
| READ | WWP2 | 0.30498988 | 0.108455806 | 0.857665723 | 0.010156126 |
| SARC | HECW1 | 1.545464357 | 1.041851111 | 2.292515746 | 0.026302019 |
| SARC | HECW2 | 1.409653313 | 0.921616256 | 2.156127836 | 0.091284446 |
| SARC | ITCH | 1.281266081 | 0.860757781 | 1.907206426 | 0.236191639 |
| SARC | NEDD4L | 0.755854221 | 0.456285278 | 1.252101769 | 0.235319882 |
| SARC | RPF1 | 1.623058597 | 1.09378653 | 2.408439981 | 0.013551181 |
| SARC | SMURF1 | 1.671142908 | 0.945883519 | 2.952497387 | 0.033407806 |
| SARC | SMURF2 | 1.506491322 | 1.021848544 | 2.220990691 | 0.041600102 |
| SARC | WWP1 | 1.816377818 | 1.05736658 | 3.120231375 | 0.008404184 |
| SARC | WWP2 | 2.05225609 | 1.037385018 | 4.059972898 | 0.005612244 |
| SKCM | HECW1 | 1.457649661 | 1.08407584 | 1.959957464 | 0.020349744 |
| SKCM | HECW2 | 1.707858635 | 1.077642599 | 2.706631236 | 0.004503474 |
| SKCM | ITCH | 1.558681043 | 1.188747389 | 2.043736639 | 0.002115925 |
| SKCM | NEDD4L | 1.254474049 | 0.91921284 | 1.712013879 | 0.124700532 |
| SKCM | RPF1 | 1.183365699 | 0.90396463 | 1.549125188 | 0.213231594 |
| SKCM | SMURF1 | 1.961383537 | 1.46125216 | 2.632690979 | 0.000110762 |
| SKCM | SMURF2 | 1.515536953 | 1.14393431 | 2.007853279 | 0.007041758 |
| SKCM | WWP1 | 1.323327423 | 1.01341283 | 1.728017858 | 0.039696356 |
| SKCM | WWP2 | 1.450082429 | 0.929860273 | 2.261349486 | 0.056366608 |
| STAD | HECW1 | 1.389162385 | 0.92416832 | 2.088117598 | 0.080823714 |
| STAD | HECW2 | 1.641329645 | 1.015563645 | 2.65267767 | 0.015345283 |
| STAD | ITCH | 1.441955502 | 0.924306269 | 2.249509433 | 0.067353774 |
| STAD | NEDD4L | 0.770180476 | 0.509544933 | 1.164132793 | 0.178578129 |
| STAD | RPF1 | 0.637868157 | 0.411031414 | 0.989889756 | 0.084143872 |
| STAD | SMURF1 | 0.677408315 | 0.396669319 | 1.156837708 | 0.095355915 |
| STAD | SMURF2 | 1.468628529 | 0.91244393 | 2.363838132 | 0.06839452 |
| STAD | WWP1 | 0.746851363 | 0.496890606 | 1.122554847 | 0.125064158 |
| STAD | WWP2 | 1.681103092 | 0.943613123 | 2.994985487 | 0.028287344 |
| TGCT | HECW1 | 0.238098225 | 0.032619788 | 1.737925607 | 0.174219152 |
| TGCT | HECW2 | #NUM! | #NUM! | #NUM! | 0.276504076 |
| TGCT | ITCH | 0 | 0 | 0 | 0.307091058 |
| TGCT | NEDD4L | 0.306670413 | 0.043125158 | 2.180786041 | 0.253589965 |
| TGCT | RPF1 | 0.290378073 | 0.027785314 | 3.03467596 | 0.165832634 |
| TGCT | SMURF1 | #NUM! | #NUM! | #NUM! | 0.320454546 |
| TGCT | SMURF2 | 0.082706171 | 0.002066508 | 3.310082038 | 0.001218415 |
| TGCT | WWP1 | 0.248157357 | 0.021298869 | 2.891330732 | 0.130168662 |
| TGCT | WWP2 | 0.312138538 | 0.043922453 | 2.218238275 | 0.262370541 |
| THCA | HECW1 | 0.356250917 | 0.071974051 | 1.763339893 | 0.053016331 |
| THCA | HECW2 | 0.290534294 | 0.086098571 | 0.980389973 | 0.198710948 |
| THCA | ITCH | 0.253350996 | 0.097783641 | 0.656415805 | 0.046144329 |
| THCA | NEDD4L | 0.174532034 | 0.072644116 | 0.419324133 | 0.001540484 |
| THCA | RPF1 | 1.984547 | 0.756866549 | 5.203594745 | 0.118126039 |
| THCA | SMURF1 | 0.366265427 | 0.089652498 | 1.496337146 | 0.042094538 |
| THCA | SMURF2 | 3.708153852 | 0.880205688 | 15.62180883 | 0.003893472 |
| THCA | WWP1 | 2.244340615 | 0.740076018 | 6.80614515 | 0.075695541 |
| THCA | WWP2 | 1.901691608 | 0.73133788 | 4.944952351 | 0.145187156 |
| THYM | HECW1 | 5.597113995 | 1.461468424 | 21.4357591 | 0.015128008 |
| THYM | HECW2 | 6.556700028 | 1.400378702 | 30.69906391 | 0.002065338 |
| THYM | ITCH | 11.21157294 | 2.979199965 | 42.19232322 | 0.003600848 |
| THYM | NEDD4L | 13.78506009 | 3.554743107 | 53.45755684 | 0.001143255 |
| THYM | RPF1 | 0 | 0 | 0 | 0.09340903 |
| THYM | SMURF1 | #NUM! | #NUM! | #NUM! | 0.000468001 |
| THYM | SMURF2 | 11.48936542 | 3.04444795 | 43.35942668 | 0.003434046 |
| THYM | WWP1 | 3.770517229 | 0.955378786 | 14.88079952 | 0.042571956 |
| THYM | WWP2 | #NUM! | #NUM! | #NUM! | 0.000712131 |
| UCEC | HECW1 | 0.618438159 | 0.394507901 | 0.969475531 | 0.051210688 |
| UCEC | HECW2 | 0.438203192 | 0.281023822 | 0.68329452 | 0.000181022 |
| UCEC | ITCH | 1.935081235 | 1.128659406 | 3.317687662 | 0.004184144 |
| UCEC | NEDD4L | 0.566156912 | 0.337503235 | 0.949720227 | 0.0132442 |
| UCEC | RPF1 | 0.651869036 | 0.333421255 | 1.274463564 | 0.140881401 |
| UCEC | SMURF1 | 1.347400082 | 0.661080406 | 2.746242308 | 0.354668154 |
| UCEC | SMURF2 | 0.435706164 | 0.281293133 | 0.674882673 | 0.000231291 |
| UCEC | WWP1 | 0.56539035 | 0.336982505 | 0.948613782 | 0.013103961 |
| UCEC | WWP2 | 2.031324831 | 1.127027223 | 3.66120754 | 0.003245676 |
| UCS | HECW1 | 1.776392457 | 0.913589032 | 3.454036825 | 0.089097642 |
| UCS | HECW2 | 2.870433298 | 0.670157575 | 12.29470146 | 0.020933191 |
| UCS | ITCH | 0.461324268 | 0.224419408 | 0.948314064 | 0.015470006 |
| UCS | NEDD4L | 6.02895275 | 2.387684363 | 15.22323127 | 0.039297403 |
| UCS | RPF1 | 1.530787836 | 0.788486657 | 2.971910024 | 0.214859675 |
| UCS | SMURF1 | 0.546471214 | 0.235264881 | 1.269338569 | 0.240194042 |
| UCS | SMURF2 | 0.671516354 | 0.315367105 | 1.429870796 | 0.250899665 |
| UCS | WWP1 | 0.398763822 | 0.149817656 | 1.061374142 | 0.185872523 |
| UCS | WWP2 | 0.452641354 | 0.215715002 | 0.94979113 | 0.013516622 |
| UVM | HECW1 | 4.223513716 | 1.263266724 | 14.12058734 | 0.000230144 |
| UVM | HECW2 | 6.034970997 | 2.592925903 | 14.04624594 | 3.28561E-05 |
| UVM | ITCH | 3.101162453 | 1.113671411 | 8.635588975 | 0.004028392 |
| UVM | NEDD4L | 0.278486224 | 0.1090198 | 0.711380657 | 0.063984864 |
| UVM | RPF1 | 2.166331794 | 0.956360652 | 4.907137736 | 0.075956219 |
| UVM | SMURF1 | 0.305245997 | 0.101241204 | 0.920328037 | 0.002942764 |
| UVM | SMURF2 | 0.06864083 | 0.029980395 | 0.157154819 | 6.92652E-07 |
| UVM | WWP1 | 0.079875717 | 0.035161328 | 0.181453048 | 7.86981E-06 |
| UVM | WWP2 | 0.036870224 | 0.016208935 | 0.083868152 | 6.70647E-07 |

Supplementary Table 3. Mutation analysis of NEDD4 family genes in different cancers.

| Cancer | HECW1 | HECW2 | ITCH | NEDD4L | RPF1 | SMURF1 | SMURF2 | WWP1 | WWP2 |
| --- | --- | --- | --- | --- | --- | --- | --- | --- | --- |
| ACC | 0.043478 | 0.021739 | 0.01087 | 0.032609 | 0 | 0 | 0 | 0.021739 | 0.01087 |
| BLCA | 0.058252 | 0.046117 | 0.029126 | 0.014563 | 0.012136 | 0.026699 | 0.021845 | 0.012136 | 0.019417 |
| BRCA | 0.028398 | 0.024341 | 0.007099 | 0.017241 | 0.001014 | 0.009128 | 0.005071 | 0.013185 | 0.020284 |
| CESC | 0.069204 | 0.048443 | 0.044983 | 0.034602 | 0.013841 | 0.031142 | 0.013841 | 0.024221 | 0.038062 |
| CHOL | 0 | 0.039216 | 0.019608 | 0.039216 | 0 | 0.019608 | 0.019608 | 0.019608 | 0.039216 |
| COAD | 0.182957 | 0.090226 | 0.032581 | 0.072682 | 0.017544 | 0.042607 | 0.012531 | 0.067669 | 0.037594 |
| DLBC | 0.027027 | 0.027027 | 0.027027 | 0.027027 | 0 | 0.027027 | 0 | 0 | 0 |
| ESCA | 0.038043 | 0.038043 | 0.038043 | 0.01087 | 0.01087 | 0.016304 | 0.005435 | 0.016304 | 0.021739 |
| GBM | 0.050891 | 0.035623 | 0.012723 | 0.022901 | 0.005089 | 0.040712 | 0.007634 | 0.005089 | 0.010178 |
| HNSC | 0.025591 | 0.035433 | 0.015748 | 0.011811 | 0.009843 | 0.009843 | 0.01378 | 0.019685 | 0.019685 |
| KICH | 0 | 0.015152 | 0 | 0.015152 | 0 | 0 | 0 | 0.015152 | 0 |
| KIRC | 0.017857 | 0.014881 | 0.005952 | 0.002976 | 0.005952 | 0.005952 | 0.008929 | 0.002976 | 0.005952 |
| KIRP | 0.017794 | 0.007117 | 0.003559 | 0.007117 | 0 | 0 | 0.003559 | 0.007117 | 0.007117 |
| LAML | 0.013986 | 0.006993 | 0.006993 | 0 | 0 | 0 | 0 | 0 | 0.006993 |
| LGG | 0.017717 | 0.005906 | 0.001969 | 0.001969 | 0.001969 | 0.003937 | 0.003937 | 0.009843 | 0.001969 |
| LIHC | 0.03022 | 0.027473 | 0.008242 | 0.021978 | 0.002747 | 0.005495 | 0.002747 | 0.002747 | 0.008242 |
| LUAD | 0.15873 | 0.058201 | 0.021164 | 0.012346 | 0.008818 | 0.012346 | 0.012346 | 0.028219 | 0.014109 |
| LUSC | 0.111789 | 0.077236 | 0.028455 | 0.026423 | 0.002033 | 0.012195 | 0.00813 | 0.02439 | 0.020325 |
| MESO | 0 | 0 | 0.012195 | 0.012195 | 0 | 0.036585 | 0 | 0.012195 | 0.04878 |
| OV | 0.048165 | 0.041284 | 0.009174 | 0.004587 | 0.004587 | 0.022936 | 0.002294 | 0.004587 | 0.016055 |
| PAAD | 0.044944 | 0.067416 | 0.016854 | 0.005618 | 0 | 0 | 0 | 0.011236 | 0.02809 |
| PCPG | 0 | 0.005587 | 0 | 0 | 0 | 0 | 0 | 0.005587 | 0 |
| PRAD | 0.016162 | 0.00202 | 0.006061 | 0 | 0.00202 | 0.00404 | 0.00404 | 0.00404 | 0.00202 |
| READ | 0.138686 | 0.10219 | 0.021898 | 0.036496 | 0.014599 | 0.036496 | 0.014599 | 0.072993 | 0 |
| SARC | 0.012658 | 0.012658 | 0.004219 | 0.008439 | 0 | 0.004219 | 0.012658 | 0.008439 | 0.004219 |
| SKCM | 0.24197 | 0.192719 | 0.023555 | 0.042827 | 0.008565 | 0.012848 | 0.036403 | 0.027837 | 0.047109 |
| STAD | 0.121281 | 0.05492 | 0.036613 | 0.034325 | 0.006865 | 0.034325 | 0.02746 | 0.05492 | 0.05492 |
| TGCT | 0 | 0.006944 | 0 | 0 | 0.006944 | 0 | 0 | 0 | 0 |
| THCA | 0.002033 | 0.004065 | 0.002033 | 0 | 0 | 0.004065 | 0.002033 | 0 | 0 |
| THYM | 0.00813 | 0.01626 | 0 | 0 | 0 | 0 | 0.00813 | 0 | 0 |
| UCEC | 0.320755 | 0.3 | 0.135849 | 0.173585 | 0.045283 | 0.183019 | 0.122642 | 0.149057 | 0.133962 |
| UCS | 0.017544 | 0.052632 | 0.035088 | 0 | 0.017544 | 0 | 0 | 0.035088 | 0 |
| UVM | 0 | 0 | 0 | 0 | 0 | 0 | 0 | 0 | 0 |

Supplementary Table 4. Copy number variation analysis of NEDD4 family genes in different cancers.

| Cancer | gene | GainNumber | LostNumber |
| --- | --- | --- | --- |
| ACC | HECW1 | 0.00197772 | 0.000201808 |
| ACC | HECW2 | 0.000565063 | 0.000605425 |
| ACC | ITCH | 0.002018082 | 0.000161447 |
| ACC | NEDD4L | 0.000161447 | 0.001574104 |
| ACC | RPF1 | 0.000201808 | 0.001291572 |
| ACC | SMURF1 | 0.002058444 | 0.000282531 |
| ACC | SMURF2 | 0.00048434 | 0.00072651 |
| ACC | WWP1 | 0.001574104 | 0.000443978 |
| ACC | WWP2 | 0.002098805 | 0.00024217 |
| BLCA | HECW1 | 0.007265095 | 0.000928318 |
| BLCA | HECW2 | 0.001614466 | 0.005206652 |
| BLCA | ITCH | 0.010292218 | 0.000121085 |
| BLCA | NEDD4L | 0.002179529 | 0.007224734 |
| BLCA | RPF1 | 0.003309655 | 0.00246206 |
| BLCA | SMURF1 | 0.006134969 | 0.001291572 |
| BLCA | SMURF2 | 0.007789797 | 0.000766871 |
| BLCA | WWP1 | 0.009444624 | 0.000847594 |
| BLCA | WWP2 | 0.004237972 | 0.004359057 |
| BRCA | HECW1 | 0.013722958 | 0.004237972 |
| BRCA | HECW2 | 0.004520504 | 0.008960284 |
| BRCA | ITCH | 0.018324185 | 0.002623507 |
| BRCA | NEDD4L | 0.006296416 | 0.014207297 |
| BRCA | RPF1 | 0.005731353 | 0.014126574 |
| BRCA | SMURF1 | 0.012229577 | 0.005610268 |
| BRCA | SMURF2 | 0.017113335 | 0.006013884 |
| BRCA | WWP1 | 0.02486277 | 0.001735551 |
| BRCA | WWP2 | 0.004399419 | 0.026840491 |
| CESC | HECW1 | 0.001654827 | 0.000968679 |
| CESC | HECW2 | 0.001896997 | 0.001856635 |
| CESC | ITCH | 0.005247013 | 0.000282531 |
| CESC | NEDD4L | 0.001210849 | 0.003350016 |
| CESC | RPF1 | 0.003430739 | 0.000928318 |
| CESC | SMURF1 | 0.001816274 | 0.001493381 |
| CESC | SMURF2 | 0.002825315 | 0.001089764 |
| CESC | WWP1 | 0.004036164 | 0.000807233 |
| CESC | WWP2 | 0.001654827 | 0.001896997 |
| CHOL | HECW1 | 0.000322893 | 0.000121085 |
| CHOL | HECW2 | 0.00024217 | 8.07233E-05 |
| CHOL | ITCH | 0.000524701 | 4.03616E-05 |
| CHOL | NEDD4L | 0.000201808 | 0.000524701 |
| CHOL | RPF1 | 0.000363255 | 0.000161447 |
| CHOL | SMURF1 | 0.00024217 | 0.000161447 |
| CHOL | SMURF2 | 0.000363255 | 4.03616E-05 |
| CHOL | WWP1 | 0.000524701 | 8.07233E-05 |
| CHOL | WWP2 | 0.000282531 | 0.000282531 |
| COAD | HECW1 | 0.01009041 | 4.03616E-05 |
| COAD | HECW2 | 0.003592186 | 0.000645786 |
| COAD | ITCH | 0.013117533 | 0 |
| COAD | NEDD4L | 0.000322893 | 0.011503067 |
| COAD | RPF1 | 0.000605425 | 0.005287375 |
| COAD | SMURF1 | 0.009041007 | 0.000201808 |
| COAD | SMURF2 | 0.004157249 | 0.002260252 |
| COAD | WWP1 | 0.010050048 | 0.000443978 |
| COAD | WWP2 | 0.004076526 | 0.001251211 |
| DLBC | HECW1 | 0.000565063 | 0.000121085 |
| DLBC | HECW2 | 0.000201808 | 0.000121085 |
| DLBC | ITCH | 0.00024217 | 0 |
| DLBC | NEDD4L | 0.000645786 | 8.07233E-05 |
| DLBC | RPF1 | 8.07233E-05 | 0.000161447 |
| DLBC | SMURF1 | 0.000565063 | 8.07233E-05 |
| DLBC | SMURF2 | 0.000161447 | 0.000201808 |
| DLBC | WWP1 | 0.000322893 | 0.000161447 |
| DLBC | WWP2 | 0.000282531 | 0.000161447 |
| ESCA | HECW1 | 0.004803035 | 0.000403616 |
| ESCA | HECW2 | 0.002139167 | 0.000928318 |
| ESCA | ITCH | 0.005004843 | 8.07233E-05 |
| ESCA | NEDD4L | 0.000968679 | 0.00468195 |
| ESCA | RPF1 | 0.000887956 | 0.002179529 |
| ESCA | SMURF1 | 0.004278334 | 0.000807233 |
| ESCA | SMURF2 | 0.00270423 | 0.000887956 |
| ESCA | WWP1 | 0.004560865 | 0.000645786 |
| ESCA | WWP2 | 0.00197772 | 0.002139167 |
| GBM | HECW1 | 0.01844527 | 0.000403616 |
| GBM | HECW2 | 0.001372296 | 0.001453019 |
| GBM | ITCH | 0.009000646 | 0.00072651 |
| GBM | NEDD4L | 0.002542783 | 0.002784953 |
| GBM | RPF1 | 0.003430739 | 0.001291572 |
| GBM | SMURF1 | 0.019091056 | 0.00024217 |
| GBM | SMURF2 | 0.002986761 | 0.001493381 |
| GBM | WWP1 | 0.002381337 | 0.001735551 |
| GBM | WWP2 | 0.001533742 | 0.003713271 |
| HNSC | HECW1 | 0.007668712 | 0.001089764 |
| HNSC | HECW2 | 0.003350016 | 0.003309655 |
| HNSC | ITCH | 0.009041007 | 0.000605425 |
| HNSC | NEDD4L | 0.001251211 | 0.011139813 |
| HNSC | RPF1 | 0.002139167 | 0.003874717 |
| HNSC | SMURF1 | 0.00690184 | 0.001453019 |
| HNSC | SMURF2 | 0.004480142 | 0.001493381 |
| HNSC | WWP1 | 0.013763319 | 0.000766871 |
| HNSC | WWP2 | 0.004601227 | 0.003511463 |
| KICH | HECW1 | 0.000968679 | 4.03616E-05 |
| KICH | HECW2 | 4.03616E-05 | 0.001896997 |
| KICH | ITCH | 0.000887956 | 0.000121085 |
| KICH | NEDD4L | 0.000645786 | 0.000363255 |
| KICH | RPF1 | 4.03616E-05 | 0.002139167 |
| KICH | SMURF1 | 0.001009041 | 4.03616E-05 |
| KICH | SMURF2 | 0 | 0.002018082 |
| KICH | WWP1 | 0.00072651 | 0.000403616 |
| KICH | WWP2 | 0.000847594 | 0.000201808 |
| KIRC | HECW1 | 0.006982564 | 0.000121085 |
| KIRC | HECW2 | 0.00318857 | 0.000847594 |
| KIRC | ITCH | 0.004803035 | 0 |
| KIRC | NEDD4L | 0.001049403 | 0.003915079 |
| KIRC | RPF1 | 0.000766871 | 0.002623507 |
| KIRC | SMURF1 | 0.007063287 | 8.07233E-05 |
| KIRC | SMURF2 | 0.001614466 | 0.001049403 |
| KIRC | WWP1 | 0.00270423 | 0.002583145 |
| KIRC | WWP2 | 0.003995802 | 0.000807233 |
| KIRP | HECW1 | 0.006942202 | 4.03616E-05 |
| KIRP | HECW2 | 0.002058444 | 0.000161447 |
| KIRP | ITCH | 0.004116887 | 4.03616E-05 |
| KIRP | NEDD4L | 0.000403616 | 0.001816274 |
| KIRP | RPF1 | 0.000161447 | 0.001412657 |
| KIRP | SMURF1 | 0.007022925 | 4.03616E-05 |
| KIRP | SMURF2 | 0.008031966 | 4.03616E-05 |
| KIRP | WWP1 | 0.001009041 | 0.000443978 |
| KIRP | WWP2 | 0.006054246 | 0.000282531 |
| LAML | HECW1 | 8.07233E-05 | 0.000686148 |
| LAML | HECW2 | 4.03616E-05 | 0 |
| LAML | ITCH | 4.03616E-05 | 0.000121085 |
| LAML | NEDD4L | 0 | 0.000161447 |
| LAML | RPF1 | 0.000161447 | 8.07233E-05 |
| LAML | SMURF1 | 0 | 0.000807233 |
| LAML | SMURF2 | 0.000161447 | 0.000201808 |
| LAML | WWP1 | 0.000928318 | 0 |
| LAML | WWP2 | 4.03616E-05 | 0.000282531 |
| LGG | HECW1 | 0.004883758 | 0.000161447 |
| LGG | HECW2 | 0.000443978 | 0.001049403 |
| LGG | ITCH | 0.001775912 | 0.000121085 |
| LGG | NEDD4L | 0.000686148 | 0.002865676 |
| LGG | RPF1 | 0.000686148 | 0.007507265 |
| LGG | SMURF1 | 0.006578947 | 0.000121085 |
| LGG | SMURF2 | 0.001533742 | 0.000403616 |
| LGG | WWP1 | 0.002583145 | 0.00048434 |
| LGG | WWP2 | 0.000807233 | 0.000968679 |
| LIHC | HECW1 | 0.004641589 | 0.000686148 |
| LIHC | HECW2 | 0.002098805 | 0.001493381 |
| LIHC | ITCH | 0.00468195 | 0.000363255 |
| LIHC | NEDD4L | 0.001210849 | 0.003672909 |
| LIHC | RPF1 | 0.001775912 | 0.003713271 |
| LIHC | SMURF1 | 0.004883758 | 0.001130126 |
| LIHC | SMURF2 | 0.005327737 | 0.000968679 |
| LIHC | WWP1 | 0.008637391 | 0.000686148 |
| LIHC | WWP2 | 0.000887956 | 0.006256054 |
| LUAD | HECW1 | 0.010776558 | 0.001412657 |
| LUAD | HECW2 | 0.005610268 | 0.001533742 |
| LUAD | ITCH | 0.008879561 | 0.002300613 |
| LUAD | NEDD4L | 0.002300613 | 0.01033258 |
| LUAD | RPF1 | 0.004722312 | 0.005610268 |
| LUAD | SMURF1 | 0.008960284 | 0.002179529 |
| LUAD | SMURF2 | 0.010171133 | 0.001049403 |
| LUAD | WWP1 | 0.011059089 | 0.001816274 |
| LUAD | WWP2 | 0.004197611 | 0.006215693 |
| LUSC | HECW1 | 0.00960607 | 0.002865676 |
| LUSC | HECW2 | 0.006013884 | 0.002784953 |
| LUSC | ITCH | 0.011543429 | 0.001130126 |
| LUSC | NEDD4L | 0.003632548 | 0.008718114 |
| LUSC | RPF1 | 0.002825315 | 0.007789797 |
| LUSC | SMURF1 | 0.01033258 | 0.001614466 |
| LUSC | SMURF2 | 0.009081369 | 0.002340975 |
| LUSC | WWP1 | 0.011381983 | 0.002018082 |
| LUSC | WWP2 | 0.003955441 | 0.007184372 |
| MESO | HECW1 | 0.000968679 | 4.03616E-05 |
| MESO | HECW2 | 0.000201808 | 0.000403616 |
| MESO | ITCH | 0.000443978 | 0.000161447 |
| MESO | NEDD4L | 0.000121085 | 0.000645786 |
| MESO | RPF1 | 0.000201808 | 0.001170488 |
| MESO | SMURF1 | 0.000928318 | 8.07233E-05 |
| MESO | SMURF2 | 0.001009041 | 0.000161447 |
| MESO | WWP1 | 0.000605425 | 0.000161447 |
| MESO | WWP2 | 0.00048434 | 0.00072651 |
| OV | HECW1 | 0.007103649 | 0.00540846 |
| OV | HECW2 | 0.009202454 | 0.003269293 |
| OV | ITCH | 0.013198256 | 0.001372296 |
| OV | NEDD4L | 0.002098805 | 0.01501453 |
| OV | RPF1 | 0.005731353 | 0.006013884 |
| OV | SMURF1 | 0.010494026 | 0.002865676 |
| OV | SMURF2 | 0.006215693 | 0.010009687 |
| OV | WWP1 | 0.011785599 | 0.003713271 |
| OV | WWP2 | 0.001331934 | 0.018243461 |
| PAAD | HECW1 | 0.002179529 | 0.000201808 |
| PAAD | HECW2 | 0.000968679 | 0.00024217 |
| PAAD | ITCH | 0.001735551 | 0.000201808 |
| PAAD | NEDD4L | 0.000282531 | 0.005004843 |
| PAAD | RPF1 | 0.000443978 | 0.001775912 |
| PAAD | SMURF1 | 0.002260252 | 0.00024217 |
| PAAD | SMURF2 | 0.001089764 | 0.001654827 |
| PAAD | WWP1 | 0.002260252 | 0.00048434 |
| PAAD | WWP2 | 0.000968679 | 0.000565063 |
| PCPG | HECW1 | 0.001170488 | 0 |
| PCPG | HECW2 | 0.000161447 | 0.000524701 |
| PCPG | ITCH | 0.000403616 | 0.000121085 |
| PCPG | NEDD4L | 0.000443978 | 0.000282531 |
| PCPG | RPF1 | 4.03616E-05 | 0.004762674 |
| PCPG | SMURF1 | 0.000847594 | 0.000322893 |
| PCPG | SMURF2 | 0.000403616 | 0.00048434 |
| PCPG | WWP1 | 0.000605425 | 0.000524701 |
| PCPG | WWP2 | 0.000282531 | 0.000201808 |
| PRAD | HECW1 | 0.004076526 | 0.000322893 |
| PRAD | HECW2 | 0.000565063 | 0.001009041 |
| PRAD | ITCH | 0.001331934 | 0.000645786 |
| PRAD | NEDD4L | 0.000524701 | 0.00540846 |
| PRAD | RPF1 | 0.000121085 | 0.00221989 |
| PRAD | SMURF1 | 0.004076526 | 0.000282531 |
| PRAD | SMURF2 | 0.000847594 | 0.000887956 |
| PRAD | WWP1 | 0.006134969 | 0.000322893 |
| PRAD | WWP2 | 0.000322893 | 0.006175331 |
| READ | HECW1 | 0.004237972 | 4.03616E-05 |
| READ | HECW2 | 0.001816274 | 0.00024217 |
| READ | ITCH | 0.005892799 | 0 |
| READ | NEDD4L | 4.03616E-05 | 0.005812076 |
| READ | RPF1 | 0.000322893 | 0.002260252 |
| READ | SMURF1 | 0.003471101 | 0.000121085 |
| READ | SMURF2 | 0.001533742 | 0.000807233 |
| READ | WWP1 | 0.003915079 | 0.000282531 |
| READ | WWP2 | 0.001614466 | 0.00072651 |
| SARC | HECW1 | 0.003551824 | 0.001251211 |
| SARC | HECW2 | 0.001251211 | 0.002784953 |
| SARC | ITCH | 0.003955441 | 0.000565063 |
| SARC | NEDD4L | 0.001735551 | 0.003269293 |
| SARC | RPF1 | 0.003148208 | 0.001291572 |
| SARC | SMURF1 | 0.002906038 | 0.001654827 |
| SARC | SMURF2 | 0.002986761 | 0.001816274 |
| SARC | WWP1 | 0.003551824 | 0.001654827 |
| SARC | WWP2 | 0.000928318 | 0.005327737 |
| SKCM | HECW1 | 0.008233775 | 0.000928318 |
| SKCM | HECW2 | 0.0029464 | 0.00221989 |
| SKCM | ITCH | 0.007224734 | 0.000322893 |
| SKCM | NEDD4L | 0.002139167 | 0.004157249 |
| SKCM | RPF1 | 0.002906038 | 0.003390378 |
| SKCM | SMURF1 | 0.00811269 | 0.000968679 |
| SKCM | SMURF2 | 0.004964482 | 0.001735551 |
| SKCM | WWP1 | 0.00714401 | 0.000887956 |
| SKCM | WWP2 | 0.002058444 | 0.004399419 |
| STAD | HECW1 | 0.008718114 | 0.000403616 |
| STAD | HECW2 | 0.0029464 | 0.001856635 |
| STAD | ITCH | 0.011059089 | 0.00024217 |
| STAD | NEDD4L | 0.001654827 | 0.008233775 |
| STAD | RPF1 | 0.001533742 | 0.003793994 |
| STAD | SMURF1 | 0.008072328 | 0.001251211 |
| STAD | SMURF2 | 0.003955441 | 0.002744592 |
| STAD | WWP1 | 0.010857281 | 0.000686148 |
| STAD | WWP2 | 0.002421698 | 0.004883758 |
| TGCT | HECW1 | 0.00492412 | 0.000121085 |
| TGCT | HECW2 | 0.001816274 | 0.000201808 |
| TGCT | ITCH | 0.001735551 | 0.000565063 |
| TGCT | NEDD4L | 4.03616E-05 | 0.004964482 |
| TGCT | RPF1 | 0.001654827 | 0.001009041 |
| TGCT | SMURF1 | 0.004480142 | 0.000161447 |
| TGCT | SMURF2 | 0.002300613 | 0.000443978 |
| TGCT | WWP1 | 0.004803035 | 0.000121085 |
| TGCT | WWP2 | 0.000807233 | 0.002018082 |
| THCA | HECW1 | 0.00072651 | 0 |
| THCA | HECW2 | 4.03616E-05 | 0.000403616 |
| THCA | ITCH | 0.00048434 | 0 |
| THCA | NEDD4L | 0.00024217 | 0.000161447 |
| THCA | RPF1 | 0.000121085 | 0.000161447 |
| THCA | SMURF1 | 0.000807233 | 4.03616E-05 |
| THCA | SMURF2 | 0.000847594 | 4.03616E-05 |
| THCA | WWP1 | 0.000282531 | 0.000161447 |
| THCA | WWP2 | 0.000524701 | 0.000161447 |
| THYM | HECW1 | 0.000565063 | 0.000121085 |
| THYM | HECW2 | 0 | 8.07233E-05 |
| THYM | ITCH | 0.000403616 | 4.03616E-05 |
| THYM | NEDD4L | 0.000121085 | 0.000322893 |
| THYM | RPF1 | 4.03616E-05 | 0.000282531 |
| THYM | SMURF1 | 0.000645786 | 8.07233E-05 |
| THYM | SMURF2 | 0.000403616 | 0.000161447 |
| THYM | WWP1 | 0.000403616 | 8.07233E-05 |
| THYM | WWP2 | 8.07233E-05 | 0.000524701 |
| UCEC | HECW1 | 0.003027123 | 0.001856635 |
| UCEC | HECW2 | 0.003592186 | 0.000847594 |
| UCEC | ITCH | 0.00565063 | 0.000524701 |
| UCEC | NEDD4L | 0.001654827 | 0.003753633 |
| UCEC | RPF1 | 0.001735551 | 0.001412657 |
| UCEC | SMURF1 | 0.003592186 | 0.001493381 |
| UCEC | SMURF2 | 0.002784953 | 0.002744592 |
| UCEC | WWP1 | 0.006861479 | 0.000847594 |
| UCEC | WWP2 | 0.000968679 | 0.006175331 |
| UCS | HECW1 | 0.000968679 | 0.000403616 |
| UCS | HECW2 | 0.000928318 | 0.000161447 |
| UCS | ITCH | 0.001654827 | 0.000121085 |
| UCS | NEDD4L | 0.000605425 | 0.000928318 |
| UCS | RPF1 | 0.000645786 | 0.00048434 |
| UCS | SMURF1 | 0.000807233 | 0.000605425 |
| UCS | SMURF2 | 0.001089764 | 0.000443978 |
| UCS | WWP1 | 0.001412657 | 0.000282531 |
| UCS | WWP2 | 0.000322893 | 0.001372296 |
| UVM | HECW1 | 0.000363255 | 0 |
| UVM | HECW2 | 0.000322893 | 4.03616E-05 |
| UVM | ITCH | 0.000363255 | 0 |
| UVM | NEDD4L | 4.03616E-05 | 8.07233E-05 |
| UVM | RPF1 | 8.07233E-05 | 0.000847594 |
| UVM | SMURF1 | 0.000282531 | 0 |
| UVM | SMURF2 | 0.000524701 | 0 |
| UVM | WWP1 | 0.002300613 | 0 |
| UVM | WWP2 | 4.03616E-05 | 0.000686148 |

Supplementary Table 5. Correlation between NEDD4 family genes in cancers.

| Gene | ITCH | NEDD4L | HECW2 | SMURF2 | WWP1 | HECW1 | SMURF1 | RPF1 | WWP2 |
| --- | --- | --- | --- | --- | --- | --- | --- | --- | --- |
| ITCH | 1 | 0.113907 | 0.370625 | 0.421861 | 0.239787 | 0.259806 | 0.559377 | 0.271324 | 0.732449 |
| NEDD4L | 0.113907 | 1 | -0.01093 | -0.02886 | 0.030016 | -0.00304 | 0.116617 | 0.081237 | 0.11286 |
| HECW2 | 0.370625 | -0.01093 | 1 | 0.464392 | 0.201983 | 0.308413 | 0.226409 | 0.118462 | 0.278171 |
| SMURF2 | 0.421861 | -0.02886 | 0.464392 | 1 | 0.224758 | 0.36065 | 0.332105 | 0.155733 | 0.377655 |
| WWP1 | 0.239787 | 0.030016 | 0.201983 | 0.224758 | 1 | -0.02463 | 0.241004 | 0.063906 | 0.169004 |
| HECW1 | 0.259806 | -0.00304 | 0.308413 | 0.36065 | -0.02463 | 1 | 0.216074 | 0.126369 | 0.227798 |
| SMURF1 | 0.559377 | 0.116617 | 0.226409 | 0.332105 | 0.241004 | 0.216074 | 1 | 0.189359 | 0.563329 |
| RPF1 | 0.271324 | 0.081237 | 0.118462 | 0.155733 | 0.063906 | 0.126369 | 0.189359 | 1 | 0.259602 |
| WWP2 | 0.732449 | 0.11286 | 0.278171 | 0.377655 | 0.169004 | 0.227798 | 0.563329 | 0.259602 | 1 |

Supplementary Table 6. KEGG analysis of NEDD4 family genes in cancers.

| ID | Description | GeneRatio | BgRatio | pvalue | p.adjust | qvalue |
| --- | --- | --- | --- | --- | --- | --- |
| hsa03010 | Ribosome | 84/923 | 158/8157 | 5.41E-39 | 1.64E-36 | 9.52E-37 |
| hsa05171 | Coronavirus disease - COVID-19 | 101/923 | 232/8157 | 6.82E-37 | 1.03E-34 | 5.99E-35 |
| hsa03040 | Spliceosome | 58/923 | 147/8157 | 5.64E-19 | 5.69E-17 | 3.30E-17 |
| hsa04120 | Ubiquitin mediated proteolysis | 55/923 | 142/8157 | 1.28E-17 | 9.66E-16 | 5.60E-16 |
| hsa05160 | Hepatitis C | 47/923 | 157/8157 | 1.45E-10 | 8.77E-09 | 5.09E-09 |
| hsa04144 | Endocytosis | 63/923 | 251/8157 | 4.28E-10 | 1.91E-08 | 1.11E-08 |
| hsa05161 | Hepatitis B | 47/923 | 162/8157 | 4.69E-10 | 1.91E-08 | 1.11E-08 |
| hsa04390 | Hippo signaling pathway | 46/923 | 157/8157 | 5.04E-10 | 1.91E-08 | 1.11E-08 |
| hsa05225 | Hepatocellular carcinoma | 47/923 | 168/8157 | 1.77E-09 | 5.97E-08 | 3.47E-08 |
| hsa05132 | Salmonella infection | 61/923 | 249/8157 | 2.28E-09 | 6.92E-08 | 4.02E-08 |
| hsa04141 | Protein processing in endoplasmic reticulum | 47/923 | 171/8157 | 3.34E-09 | 9.21E-08 | 5.34E-08 |
| hsa05220 | Chronic myeloid leukemia | 28/923 | 76/8157 | 5.02E-09 | 1.27E-07 | 7.35E-08 |
| hsa05210 | Colorectal cancer | 30/923 | 86/8157 | 6.35E-09 | 1.48E-07 | 8.56E-08 |
| hsa04110 | Cell cycle | 38/923 | 126/8157 | 6.82E-09 | 1.48E-07 | 8.56E-08 |
| hsa05212 | Pancreatic cancer | 27/923 | 76/8157 | 2.33E-08 | 4.71E-07 | 2.73E-07 |
| hsa03250 | Viral life cycle - HIV-1 | 24/923 | 63/8157 | 2.95E-08 | 5.59E-07 | 3.25E-07 |
| hsa05130 | Pathogenic Escherichia coli infection | 49/923 | 197/8157 | 5.36E-08 | 9.55E-07 | 5.54E-07 |
| hsa05205 | Proteoglycans in cancer | 49/923 | 205/8157 | 2.02E-07 | 3.41E-06 | 1.98E-06 |
| hsa04068 | FoxO signaling pathway | 36/923 | 131/8157 | 2.35E-07 | 3.71E-06 | 2.16E-06 |
| hsa04350 | TGF-beta signaling pathway | 29/923 | 94/8157 | 2.45E-07 | 3.71E-06 | 2.16E-06 |
| hsa04910 | Insulin signaling pathway | 37/923 | 137/8157 | 2.59E-07 | 3.73E-06 | 2.17E-06 |
| hsa04919 | Thyroid hormone signaling pathway | 34/923 | 121/8157 | 2.85E-07 | 3.93E-06 | 2.28E-06 |
| hsa04012 | ErbB signaling pathway | 27/923 | 85/8157 | 3.29E-07 | 4.34E-06 | 2.52E-06 |
| hsa05162 | Measles | 37/923 | 139/8157 | 3.85E-07 | 4.86E-06 | 2.82E-06 |
| hsa04520 | Adherens junction | 24/923 | 71/8157 | 4.09E-07 | 4.96E-06 | 2.88E-06 |
| hsa05166 | Human T-cell leukemia virus 1 infection | 51/923 | 222/8157 | 4.29E-07 | 5.00E-06 | 2.90E-06 |
| hsa05131 | Shigellosis | 55/923 | 247/8157 | 4.46E-07 | 5.01E-06 | 2.91E-06 |
| hsa04137 | Mitophagy - animal | 24/923 | 72/8157 | 5.49E-07 | 5.94E-06 | 3.45E-06 |
| hsa01521 | EGFR tyrosine kinase inhibitor resistance | 25/923 | 79/8157 | 9.71E-07 | 1.01E-05 | 5.88E-06 |
| hsa05170 | Human immunodeficiency virus 1 infection | 48/923 | 212/8157 | 1.47E-06 | 1.48E-05 | 8.61E-06 |
| hsa04722 | Neurotrophin signaling pathway | 32/923 | 119/8157 | 1.84E-06 | 1.80E-05 | 1.05E-05 |
| hsa05135 | Yersinia infection | 35/923 | 137/8157 | 2.24E-06 | 2.12E-05 | 1.23E-05 |
| hsa05214 | Glioma | 23/923 | 75/8157 | 4.79E-06 | 4.40E-05 | 2.55E-05 |
| hsa05169 | Epstein-Barr virus infection | 45/923 | 202/8157 | 4.95E-06 | 4.41E-05 | 2.56E-05 |
| hsa04071 | Sphingolipid signaling pathway | 31/923 | 119/8157 | 5.48E-06 | 4.75E-05 | 2.75E-05 |
| hsa05203 | Viral carcinogenesis | 45/923 | 204/8157 | 6.52E-06 | 5.48E-05 | 3.18E-05 |
| hsa04530 | Tight junction | 39/923 | 169/8157 | 8.78E-06 | 7.19E-05 | 4.17E-05 |
| hsa04210 | Apoptosis | 33/923 | 136/8157 | 1.42E-05 | 0.0001132 | 6.57E-05 |
| hsa05163 | Human cytomegalovirus infection | 47/923 | 225/8157 | 1.89E-05 | 0.0001465 | 8.50E-05 |
| hsa03008 | Ribosome biogenesis in eukaryotes | 28/923 | 109/8157 | 2.05E-05 | 0.0001554 | 9.01E-05 |
| hsa01522 | Endocrine resistance | 26/923 | 98/8157 | 2.19E-05 | 0.0001577 | 9.15E-05 |
| hsa05231 | Choline metabolism in cancer | 26/923 | 98/8157 | 2.19E-05 | 0.0001577 | 9.15E-05 |
| hsa05418 | Fluid shear stress and atherosclerosis | 33/923 | 139/8157 | 2.30E-05 | 0.0001623 | 9.42E-05 |
| hsa04550 | Signaling pathways regulating pluripotency of stem cells | 33/923 | 143/8157 | 4.26E-05 | 0.0002884 | 0.0001673 |
| hsa05213 | Endometrial cancer | 18/923 | 58/8157 | 4.28E-05 | 0.0002884 | 0.0001673 |
| hsa04218 | Cellular senescence | 35/923 | 156/8157 | 4.72E-05 | 0.0003109 | 0.0001804 |
| hsa05022 | Pathways of neurodegeneration - multiple diseases | 82/923 | 476/8157 | 4.82E-05 | 0.0003109 | 0.0001804 |
| hsa04010 | MAPK signaling pathway | 56/923 | 294/8157 | 5.15E-05 | 0.0003191 | 0.0001851 |
| hsa04510 | Focal adhesion | 42/923 | 201/8157 | 5.16E-05 | 0.0003191 | 0.0001851 |
| hsa03015 | mRNA surveillance pathway | 25/923 | 97/8157 | 5.30E-05 | 0.0003212 | 0.0001864 |
| hsa04064 | NF-kappa B signaling pathway | 26/923 | 104/8157 | 6.62E-05 | 0.0003934 | 0.0002282 |
| hsa05235 | PD-L1 expression and PD-1 checkpoint pathway in cancer | 23/923 | 89/8157 | 9.99E-05 | 0.0005823 | 0.0003378 |
| hsa05017 | Spinocerebellar ataxia | 32/923 | 143/8157 | 0.0001039 | 0.0005939 | 0.0003446 |
| hsa04150 | mTOR signaling pathway | 34/923 | 156/8157 | 0.0001112 | 0.0006238 | 0.0003619 |
| hsa04213 | Longevity regulating pathway - multiple species | 18/923 | 62/8157 | 0.0001134 | 0.000625 | 0.0003626 |
| hsa05417 | Lipid and atherosclerosis | 43/923 | 215/8157 | 0.0001224 | 0.0006624 | 0.0003843 |
| hsa05215 | Prostate cancer | 24/923 | 97/8157 | 0.0001487 | 0.0007903 | 0.0004585 |
| hsa03020 | RNA polymerase | 12/923 | 34/8157 | 0.0002098 | 0.0010958 | 0.0006358 |
| hsa05167 | Kaposi sarcoma-associated herpesvirus infection | 39/923 | 194/8157 | 0.0002236 | 0.0011483 | 0.0006662 |
| hsa04540 | Gap junction | 22/923 | 88/8157 | 0.0002363 | 0.0011935 | 0.0006924 |
| hsa03018 | RNA degradation | 20/923 | 79/8157 | 0.0003748 | 0.001842 | 0.0010687 |
| hsa03050 | Proteasome | 14/923 | 46/8157 | 0.0003769 | 0.001842 | 0.0010687 |
| hsa05219 | Bladder cancer | 13/923 | 41/8157 | 0.0003887 | 0.0018693 | 0.0010845 |
| hsa05224 | Breast cancer | 31/923 | 147/8157 | 0.0004069 | 0.0019267 | 0.0011178 |
| hsa05211 | Renal cell carcinoma | 18/923 | 69/8157 | 0.000491 | 0.0022888 | 0.0013279 |
| hsa04960 | Aldosterone-regulated sodium reabsorption | 12/923 | 37/8157 | 0.0005139 | 0.0023591 | 0.0013687 |
| hsa05230 | Central carbon metabolism in cancer | 18/923 | 70/8157 | 0.0005925 | 0.0026683 | 0.0015481 |
| hsa04668 | TNF signaling pathway | 25/923 | 112/8157 | 0.0005988 | 0.0026683 | 0.0015481 |
| hsa04370 | VEGF signaling pathway | 16/923 | 59/8157 | 0.0006266 | 0.0027516 | 0.0015964 |
| hsa04730 | Long-term depression | 16/923 | 60/8157 | 0.0007665 | 0.0033178 | 0.0019248 |
| hsa05223 | Non-small cell lung cancer | 18/923 | 72/8157 | 0.0008506 | 0.0036299 | 0.0021059 |
| hsa04140 | Autophagy - animal | 29/923 | 141/8157 | 0.0009435 | 0.0039704 | 0.0023035 |
| hsa04720 | Long-term potentiation | 17/923 | 67/8157 | 0.0009816 | 0.0040193 | 0.0023318 |
| hsa05221 | Acute myeloid leukemia | 17/923 | 67/8157 | 0.0009816 | 0.0040193 | 0.0023318 |
| hsa04340 | Hedgehog signaling pathway | 15/923 | 56/8157 | 0.0010557 | 0.0042651 | 0.0024745 |
| hsa04810 | Regulation of actin cytoskeleton | 40/923 | 218/8157 | 0.0012664 | 0.0050491 | 0.0029293 |
| hsa04114 | Oocyte meiosis | 27/923 | 131/8157 | 0.0013488 | 0.0053076 | 0.0030793 |
| hsa04931 | Insulin resistance | 23/923 | 108/8157 | 0.0019018 | 0.0073879 | 0.0042862 |
| hsa05226 | Gastric cancer | 29/923 | 149/8157 | 0.0023006 | 0.0088239 | 0.0051193 |
| hsa05016 | Huntington disease | 51/923 | 306/8157 | 0.0026904 | 0.010134 | 0.0058794 |
| hsa05164 | Influenza A | 32/923 | 171/8157 | 0.0027091 | 0.010134 | 0.0058794 |
| hsa04915 | Estrogen signaling pathway | 27/923 | 138/8157 | 0.0029666 | 0.0109618 | 0.0063597 |
| hsa04664 | Fc epsilon RI signaling pathway | 16/923 | 68/8157 | 0.0031649 | 0.0115536 | 0.006703 |
| hsa04912 | GnRH signaling pathway | 20/923 | 93/8157 | 0.0032429 | 0.0116151 | 0.0067387 |
| hsa05165 | Human papillomavirus infection | 54/923 | 331/8157 | 0.0032828 | 0.0116151 | 0.0067387 |
| hsa04371 | Apelin signaling pathway | 27/923 | 139/8157 | 0.0032967 | 0.0116151 | 0.0067387 |
| hsa05012 | Parkinson disease | 45/923 | 266/8157 | 0.0034992 | 0.0121868 | 0.0070703 |
| hsa04152 | AMPK signaling pathway | 24/923 | 120/8157 | 0.0036772 | 0.0126613 | 0.0073456 |
| hsa04670 | Leukocyte transendothelial migration | 23/923 | 114/8157 | 0.0039357 | 0.0132674 | 0.0076972 |
| hsa04360 | Axon guidance | 33/923 | 182/8157 | 0.0039408 | 0.0132674 | 0.0076972 |
| hsa04659 | Th17 cell differentiation | 22/923 | 108/8157 | 0.0042026 | 0.0139934 | 0.0081184 |
| hsa04914 | Progesterone-mediated oocyte maturation | 21/923 | 102/8157 | 0.0044756 | 0.0147403 | 0.0085518 |
| hsa04310 | Wnt signaling pathway | 31/923 | 170/8157 | 0.0046836 | 0.0152594 | 0.008853 |
| hsa04151 | PI3K-Akt signaling pathway | 56/923 | 354/8157 | 0.0054473 | 0.017559 | 0.0101871 |
| hsa04660 | T cell receptor signaling pathway | 21/923 | 104/8157 | 0.005662 | 0.0180587 | 0.010477 |
| hsa05416 | Viral myocarditis | 14/923 | 60/8157 | 0.006052 | 0.0191018 | 0.0110821 |
| hsa05014 | Amyotrophic lateral sclerosis | 57/923 | 364/8157 | 0.0062966 | 0.0196687 | 0.0114111 |
| hsa05216 | Thyroid cancer | 10/923 | 37/8157 | 0.0065581 | 0.0202666 | 0.011758 |
| hsa04115 | p53 signaling pathway | 16/923 | 73/8157 | 0.0066218 | 0.0202666 | 0.011758 |
| hsa04015 | Rap1 signaling pathway | 36/923 | 210/8157 | 0.0068524 | 0.0207627 | 0.0120457 |
| hsa04921 | Oxytocin signaling pathway | 28/923 | 154/8157 | 0.0072659 | 0.0217976 | 0.0126462 |
| hsa04933 | AGE-RAGE signaling pathway in diabetic complications | 20/923 | 100/8157 | 0.0076204 | 0.022637 | 0.0131332 |
| hsa04657 | IL-17 signaling pathway | 19/923 | 94/8157 | 0.008165 | 0.0240192 | 0.0139351 |
| hsa04916 | Melanogenesis | 20/923 | 101/8157 | 0.008523 | 0.0248315 | 0.0144063 |
| hsa04936 | Alcoholic liver disease | 26/923 | 142/8157 | 0.0086424 | 0.0249395 | 0.014469 |
| hsa04662 | B cell receptor signaling pathway | 17/923 | 82/8157 | 0.009291 | 0.0264504 | 0.0153456 |
| hsa04926 | Relaxin signaling pathway | 24/923 | 129/8157 | 0.0093406 | 0.0264504 | 0.0153456 |
| hsa04917 | Prolactin signaling pathway | 15/923 | 70/8157 | 0.0103971 | 0.0291695 | 0.0169231 |
| hsa05100 | Bacterial invasion of epithelial cells | 16/923 | 77/8157 | 0.0111726 | 0.0308135 | 0.0178768 |
| hsa04217 | Necroptosis | 28/923 | 159/8157 | 0.0111864 | 0.0308135 | 0.0178768 |
| hsa04666 | Fc gamma R-mediated phagocytosis | 19/923 | 97/8157 | 0.0114329 | 0.0312088 | 0.0181062 |
| hsa04330 | Notch signaling pathway | 13/923 | 59/8157 | 0.0130718 | 0.0353638 | 0.0205168 |
| hsa05145 | Toxoplasmosis | 21/923 | 112/8157 | 0.0132542 | 0.0355402 | 0.0206191 |
| hsa05218 | Melanoma | 15/923 | 72/8157 | 0.0134479 | 0.0357432 | 0.0207369 |
| hsa05222 | Small cell lung cancer | 18/923 | 92/8157 | 0.013756 | 0.0362441 | 0.0210275 |
| hsa04621 | NOD-like receptor signaling pathway | 31/923 | 184/8157 | 0.0146628 | 0.0383002 | 0.0222204 |
| hsa04380 | Osteoclast differentiation | 23/923 | 128/8157 | 0.016135 | 0.0417854 | 0.0242424 |
| hsa03013 | Nucleocytoplasmic transport | 20/923 | 108/8157 | 0.0175215 | 0.0449917 | 0.0261026 |
| hsa05010 | Alzheimer disease | 57/923 | 384/8157 | 0.0182074 | 0.0463601 | 0.0268964 |
| hsa05142 | Chagas disease | 19/923 | 102/8157 | 0.0191081 | 0.048248 | 0.0279917 |

Supplementary Table 7. Relationship between NEDD4 family genes and prognosis in cancers.

| Gene | HR | Low.High | Upper | Lower | P | CancerType |
| --- | --- | --- | --- | --- | --- | --- |
| HECW2 | 6.15049836074412 | 12:67 | 15.9487070965468 | 2.37189321093662 | 0.0399201187018086 | ACC |
| ITCH | 3.46755388300722 | 62:17 | 10.1460219670953 | 1.18508810354969 | 0.00059430416531725 | ACC |
| NEDD4L | 4.80908822389903 | 59:20 | 12.2818289891305 | 1.88305256209903 | 3.59327128707232e-06 | ACC |
| RPF1 | 5.00228988426142 | 42:37 | 10.7513485363364 | 2.32742004424972 | 3.60566244129723e-05 | ACC |
| SMURF1 | 4.99906217276341 | 40:39 | 10.5799621954561 | 2.3620710684474 | 9.3597576103388e-05 | ACC |
| SMURF2 | 4.18243360621404 | 45:34 | 9.16503559953819 | 1.90863970798652 | 9.57707227836613e-05 | ACC |
| WWP1 | 5.87091076582997 | 69:10 | 30.34823792428 | 1.13573622647668 | 2.6996579500782e-06 | ACC |
| WWP2 | 6.55752679038409 | 13:66 | 16.6888870156886 | 2.57663423367784 | 0.0314822387891242 | ACC |
| HECW1 | 1.63517783228497 | 342:66 | 2.48973650185641 | 1.07393153500482 | 0.00633995572929125 | BLCA |
| HECW2 | 1.44341043286891 | 307:101 | 2.04449972132869 | 1.01904326813056 | 0.0230793732421015 | BLCA |
| ITCH | 1.82541679269438 | 63:345 | 2.74606172747543 | 1.21342737263011 | 0.019538604603854 | BLCA |
| NEDD4L | 0.742640928534787 | 244:164 | 1.00155515560981 | 0.550659188009681 | 0.0592705447788727 | BLCA |
| RPF1 | 1.61637115021579 | 356:52 | 2.57808056419995 | 1.01341119107373 | 0.0152055326118392 | BLCA |
| SMURF1 | 1.32458419209833 | 198:210 | 1.77697615674829 | 0.98736456046063 | 0.0616414434611366 | BLCA |
| SMURF2 | 1.62215079592034 | 57:351 | 2.51114043989805 | 1.0478797453526 | 0.0694757905454597 | BLCA |
| WWP1 | 0.810081459904799 | 109:299 | 1.13966624042095 | 0.575810661408294 | 0.202189079195176 | BLCA |
| WWP2 | 0.653279003846896 | 343:65 | 0.973019956039354 | 0.438607095587597 | 0.0703028104817357 | BLCA |
| HECW1 | 1.42281932851906 | 942:155 | 2.19965345665422 | 0.920333535031762 | 0.0723485713320263 | BRCA |
| HECW2 | 1.43475267895403 | 389:708 | 1.98441294221065 | 1.0373421811453 | 0.0360016789087583 | BRCA |
| ITCH | 1.33189334163591 | 910:187 | 2.00755009983195 | 0.883634173634104 | 0.133174605521567 | BRCA |
| NEDD4L | 0.652373350226773 | 128:969 | 1.07595136293604 | 0.395548537551695 | 0.0482610594344284 | BRCA |
| RPF1 | 0.624114287243849 | 378:719 | 0.873153125492392 | 0.446105765609255 | 0.00332715251600457 | BRCA |
| SMURF1 | 1.66288097314788 | 177:920 | 2.50754721733387 | 1.10274020434889 | 0.0386666592503164 | BRCA |
| SMURF2 | 0.678623225564382 | 969:128 | 1.07396211397431 | 0.428813527295827 | 0.149948095431668 | BRCA |
| WWP1 | 1.3587227410158 | 931:166 | 2.14031371743097 | 0.862549948597906 | 0.140199651352576 | BRCA |
| WWP2 | 1.69373366049251 | 165:932 | 2.49432949062341 | 1.15010215108686 | 0.0182871681880087 | BRCA |
| HECW1 | 1.98694304135706 | 195:111 | 3.25678041332853 | 1.21222254759335 | 0.00287370526845809 | CESC |
| HECW2 | 0.416825952518001 | 269:37 | 0.840764305339199 | 0.206649917924909 | 0.0786672479884718 | CESC |
| ITCH | 2.07726654847394 | 51:255 | 3.77600395192446 | 1.14275206497325 | 0.0595986215642833 | CESC |
| NEDD4L | 1.73646541995521 | 262:44 | 3.51663399401139 | 0.857442702264471 | 0.0602789720247596 | CESC |
| RPF1 | 0.702915376856644 | 35:271 | 1.50871609588761 | 0.327490392903136 | 0.297692970419414 | CESC |
| SMURF1 | 4.9414037601613 | 37:269 | 9.96599712058068 | 2.45007808305623 | 0.0130888301196951 | CESC |
| SMURF2 | 1.72509450386058 | 253:53 | 3.18125790038195 | 0.935463624905316 | 0.0384997266078123 | CESC |
| WWP1 | 1.84849272489453 | 109:197 | 3.00438558906725 | 1.13731252287388 | 0.0274594138460531 | CESC |
| WWP2 | 2.78695608301892 | 31:275 | 5.86449128795654 | 1.32443273035922 | 0.0687621140509932 | CESC |
| HECW1 | 2.46478477741661 | 8:28 | 6.5825894404832 | 0.922914007308755 | 0.113464139568815 | CHOL |
| HECW2 | 0.471492408667297 | 28:8 | 1.46700412332677 | 0.151536787045125 | 0.300210006153944 | CHOL |
| ITCH | 0.342539549723818 | 15:21 | 0.877339084995928 | 0.133737736220359 | 0.0243702356940783 | CHOL |
| NEDD4L | 2.65871118481645 | 32:4 | 12.6608003921024 | 0.55831740058689 | 0.0704037140363589 | CHOL |
| RPF1 | 0.0946181085585152 | 3:33 | 2.89755635063408 | 0.00308970228145239 | 1.68585062487736e-06 | CHOL |
| SMURF1 | 0.530616970170007 | 18:18 | 1.34183469949693 | 0.209827908860872 | 0.181833015844458 | CHOL |
| SMURF2 | 0.52195206201382 | 12:24 | 1.38127939523894 | 0.197233055078876 | 0.158432843817242 | CHOL |
| WWP1 | 0.318343967907246 | 13:23 | 0.848127745929922 | 0.119490115008339 | 0.0118892824723026 | CHOL |
| WWP2 | 0.356785123169803 | 6:30 | 1.29467710006973 | 0.0983222952722616 | 0.0263638990382258 | CHOL |
| HECW1 | 1.83624206360309 | 83:373 | 2.9533677999384 | 1.14167457104925 | 0.0363134532834879 | COAD |
| HECW2 | 1.41623732973621 | 200:256 | 2.08960371556223 | 0.959860551166126 | 0.0846386511996999 | COAD |
| ITCH | 0.52295620981987 | 336:120 | 0.819677016822257 | 0.333647512101059 | 0.0177296321840463 | COAD |
| NEDD4L | 0.673727762463754 | 292:164 | 1.01249164107164 | 0.448308983009469 | 0.0750334934116705 | COAD |
| RPF1 | 0.608712549703943 | 155:301 | 0.918754572902828 | 0.403297005637069 | 0.0116247830287696 | COAD |
| SMURF1 | 1.54871923917879 | 291:165 | 2.34089589538828 | 1.0246210805563 | 0.0262397910865837 | COAD |
| SMURF2 | 0.6686772514715 | 216:240 | 0.985780291747523 | 0.45357902808428 | 0.043066607778084 | COAD |
| WWP1 | 0.734889677045028 | 154:302 | 1.1101375127465 | 0.486482828682386 | 0.124911015688668 | COAD |
| WWP2 | 1.41889788529948 | 103:353 | 2.21207600982352 | 0.910127500125073 | 0.155756916786359 | COAD |
| HECW1 | 2.50127486605978 | 28:20 | 9.31495935546423 | 0.671648229137179 | 0.138574661155937 | DLBC |
| HECW2 | 3.05147172671032 | 26:22 | 11.3060687231653 | 0.823582442925895 | 0.116976922850784 | DLBC |
| ITCH | 0 | 41:7 | 0 | 0 | 0.17455582619236 | DLBC |
| NEDD4L | 0.244368474365087 | 27:21 | 0.906247814775128 | 0.065893622351341 | 0.0424599913520959 | DLBC |
| RPF1 | 0.260167622924937 | 8:40 | 2.01497875661352 | 0.0335920127178764 | 0.0362787566494325 | DLBC |
| SMURF1 | 1.9926224041443 | 26:22 | 7.62757873203821 | 0.520551040505197 | 0.266283065423164 | DLBC |
| SMURF2 | 0.369591984995685 | 5:43 | 2.23730709331882 | 0.0610547545220631 | 0.12614829725122 | DLBC |
| WWP1 | 1.88325298655187 | 25:23 | 7.15045569835583 | 0.496002207547703 | 0.315413482312695 | DLBC |
| WWP2 | 3.39688115400722 | 32:16 | 14.8227711509514 | 0.778451037052462 | 0.0456334313750439 | DLBC |
| HECW1 | 0.584794478899296 | 17:145 | 1.33417885082118 | 0.256325890895819 | 0.11259428829479 | ESCA |
| HECW2 | 1.38836700621252 | 83:79 | 2.25770032986655 | 0.853772716618005 | 0.183972162153765 | ESCA |
| ITCH | 0.536501890091048 | 130:32 | 0.93841306395552 | 0.306724500251533 | 0.0630409824077514 | ESCA |
| NEDD4L | 0.399987180730103 | 127:35 | 0.675809139285104 | 0.236738060271956 | 0.00479671855781771 | ESCA |
| RPF1 | 0.464581227205234 | 16:146 | 1.37383131177159 | 0.15710496246675 | 0.0468269682900952 | ESCA |
| SMURF1 | 0.6918582596536 | 35:127 | 1.21455680997742 | 0.394109067207657 | 0.151088024386277 | ESCA |
| SMURF2 | 0.602221863159263 | 118:44 | 1.01722196702985 | 0.356531007215626 | 0.0859412721492254 | ESCA |
| WWP1 | 0.652765883593524 | 78:84 | 1.06629143391577 | 0.399612418547566 | 0.0787298623081877 | ESCA |
| WWP2 | 1.5401025923524 | 26:136 | 3.00576473466724 | 0.789122304754554 | 0.274514342514918 | ESCA |
| HECW1 | 1.43011910918196 | 50:116 | 2.03817279464907 | 1.00346774906273 | 0.0542510770753668 | GBM |
| HECW2 | 1.36576182218238 | 18:148 | 2.20347386916471 | 0.846529373928105 | 0.216446372029398 | GBM |
| ITCH | 0.701274537951141 | 143:23 | 1.09876192752916 | 0.4475819240338 | 0.164976979106057 | GBM |
| NEDD4L | 1.34228875158374 | 69:97 | 1.88404024533456 | 0.956316669503151 | 0.0835988489448094 | GBM |
| RPF1 | 0.633257474577382 | 62:104 | 0.91246431885983 | 0.439485710092436 | 0.00766106025674507 | GBM |
| SMURF1 | 1.42261983645099 | 82:84 | 2.0005657677199 | 1.01163742363266 | 0.0356442875174086 | GBM |
| SMURF2 | 1.55711505143716 | 16:150 | 2.52450570229933 | 0.960428523177355 | 0.0984900760057691 | GBM |
| WWP1 | 1.42534832005744 | 146:20 | 2.55685699839526 | 0.794576245275217 | 0.165951593217242 | GBM |
| WWP2 | 0.714210170568811 | 16:150 | 1.32117913562875 | 0.386091601046345 | 0.213947348838643 | GBM |
| HECW1 | 1.44468427373689 | 155:347 | 1.92165206772671 | 1.08610329925735 | 0.0188119789704239 | HNSC |
| HECW2 | 0.722755578806821 | 400:102 | 0.992703498125033 | 0.526215156572954 | 0.0667336378422873 | HNSC |
| ITCH | 1.32460002183764 | 118:384 | 1.80312976422164 | 0.97306652725 | 0.0969197831951596 | HNSC |
| NEDD4L | 1.49306508217016 | 71:431 | 2.12918508541052 | 1.04699368545782 | 0.0498541494812159 | HNSC |
| RPF1 | 1.8112126449478 | 63:439 | 2.59719132081463 | 1.26309187117946 | 0.00829055961940206 | HNSC |
| SMURF1 | 1.36036653348598 | 126:376 | 1.84020056238338 | 1.00564967931094 | 0.0617935308576624 | HNSC |
| SMURF2 | 2.41350368634014 | 51:451 | 3.72845897929443 | 1.56230766553311 | 0.00485132368968422 | HNSC |
| WWP1 | 0.800435555508798 | 370:132 | 1.0771350611788 | 0.594815916419533 | 0.16194315963367 | HNSC |
| WWP2 | 1.24526822113855 | 211:291 | 1.62966424163 | 0.951541368439519 | 0.115786829760061 | HNSC |
| HECW2 | Inf | 14:51 | Inf | Inf | 0.100998045649375 | KICH |
| ITCH | 10.7716063446893 | 58:7 | 141.494052464633 | 0.820016822077748 | 7.8986858893515e-06 | KICH |
| NEDD4L | 2.62881562094856 | 58:7 | 23.6563319697947 | 0.292127772715017 | 0.209938172251247 | KICH |
| RPF1 | 15.5135685367731 | 58:7 | 319.59686348609 | 0.753044964584364 | 3.09102823337071e-08 | KICH |
| SMURF1 | 8.59129411195835 | 56:9 | 61.1133387749666 | 1.20776144779059 | 0.000107557364212751 | KICH |
| SMURF2 | 5.82277540101606 | 45:20 | 26.0343776478533 | 1.30230550656061 | 0.00451813922053812 | KICH |
| WWP1 | 5.89869974556762 | 46:19 | 26.5000024654325 | 1.31300586608423 | 0.0042427618767733 | KICH |
| WWP2 | 0.283262384360998 | 14:51 | 1.52507512790707 | 0.0526122136054972 | 0.0444891934007045 | KICH |
| HECW1 | 0.81489641473556 | 390:141 | 1.1321675918288 | 0.586535219292235 | 0.242811315873792 | KIRC |
| HECW2 | 0.444800685009757 | 175:356 | 0.61656289613477 | 0.320888023955796 | 4.21635885095029e-08 | KIRC |
| ITCH | 0.461277920460405 | 110:421 | 0.677180633044067 | 0.314210580636067 | 9.00958057603596e-07 | KIRC |
| NEDD4L | 0.399327061954132 | 236:295 | 0.539910139268474 | 0.295349338363927 | 2.242181107448e-09 | KIRC |
| RPF1 | 0.52064120333633 | 198:333 | 0.710058859468409 | 0.381753229323043 | 1.21709404529158e-05 | KIRC |
| SMURF1 | 0.639367587916929 | 133:398 | 0.90256749809071 | 0.452920045695716 | 0.00485026897632901 | KIRC |
| SMURF2 | 0.598856494816741 | 241:290 | 0.807692014608852 | 0.444017143784527 | 0.000770423263807607 | KIRC |
| WWP1 | 0.454228756180529 | 200:331 | 0.620036584787635 | 0.332760627361975 | 1.12388596984481e-07 | KIRC |
| WWP2 | 0.476745553209981 | 122:409 | 0.68541226914641 | 0.331605272821489 | 1.53964746152369e-06 | KIRC |
| HECW1 | 2.59473368356991 | 241:48 | 5.90336345249668 | 1.14047575468268 | 0.00214534920826981 | KIRP |
| HECW2 | 3.53604643208724 | 226:63 | 7.67015819296368 | 1.63016512245435 | 8.82523851319927e-06 | KIRP |
| ITCH | 1.87877936551223 | 196:93 | 3.65735472384288 | 0.965127030545634 | 0.0351004331503011 | KIRP |
| NEDD4L | 0.338710910950889 | 68:221 | 0.691100295501006 | 0.166003519234516 | 0.000172322861378138 | KIRP |
| RPF1 | 2.41472121028762 | 257:32 | 6.57623956788546 | 0.886658471489928 | 0.0146521417980602 | KIRP |
| SMURF1 | 1.75595390437814 | 103:186 | 3.21203908269043 | 0.959942900731507 | 0.091466073196157 | KIRP |
| SMURF2 | 2.03263494249383 | 100:189 | 3.74320367777853 | 1.10376168787558 | 0.0438653026133443 | KIRP |
| WWP1 | 1.83403846060285 | 139:150 | 3.3168048749273 | 1.01413776263947 | 0.0454086388834013 | KIRP |
| WWP2 | 0.33624840442006 | 148:141 | 0.607168994916997 | 0.186213377859475 | 0.000987569872688221 | KIRP |
| HECW1 | 1.65080289371755 | 24:127 | 2.81189560083425 | 0.969150559180692 | 0.111751364433822 | LAML |
| HECW2 | 0.652848613873489 | 88:63 | 0.99388528178935 | 0.428833508701531 | 0.0460915306748648 | LAML |
| ITCH | 1.44972290975455 | 75:76 | 2.21010922907144 | 0.950946897746861 | 0.0785056306924541 | LAML |
| NEDD4L | 1.629616043152 | 116:35 | 2.71459042407622 | 0.978286972703115 | 0.0311876480009671 | LAML |
| RPF1 | 2.40816626535293 | 37:114 | 3.75980582997509 | 1.54243730230672 | 0.00114070679119582 | LAML |
| SMURF1 | 0.677374742123421 | 33:118 | 1.15031704554913 | 0.398878329276372 | 0.101686413934643 | LAML |
| SMURF2 | 0.352410100613958 | 19:132 | 0.768577137774797 | 0.161588047459109 | 3.73476239443615e-05 | LAML |
| WWP1 | 0.629397574234976 | 26:125 | 1.13451313504781 | 0.34917295729342 | 0.0672478371938743 | LAML |
| WWP2 | 1.80701350030527 | 77:74 | 2.76746512297485 | 1.17988760298287 | 0.00444945652982753 | LAML |
| HECW1 | 0.269353988565252 | 63:461 | 0.538815474427045 | 0.134650125320098 | 4.05898648025982e-11 | LGG |
| HECW2 | 0.371334643307426 | 53:471 | 0.782908811308809 | 0.176124492825339 | 4.00685300423476e-05 | LGG |
| ITCH | 3.12216057228291 | 464:60 | 5.80079275916285 | 1.68044042320256 | 7.99593158351541e-09 | LGG |
| NEDD4L | 1.62036494607613 | 150:374 | 2.35460349919483 | 1.11508479426372 | 0.0233561023314586 | LGG |
| RPF1 | 3.40805399793818 | 279:245 | 4.80727121718141 | 2.41609668523599 | 7.00228763861332e-12 | LGG |
| SMURF1 | 2.51548619173993 | 256:268 | 3.53470717888254 | 1.79015416570792 | 3.74361529775769e-07 | LGG |
| SMURF2 | 1.38538521197067 | 317:207 | 1.9690010554161 | 0.974754269566113 | 0.0592583879866826 | LGG |
| WWP1 | 2.01305155483837 | 414:110 | 3.12932943948798 | 1.29496642676912 | 0.000132987016193464 | LGG |
| WWP2 | 0.566321460224166 | 470:54 | 0.994105827438891 | 0.322621583596085 | 0.110193601005089 | LGG |
| HECW1 | 1.87395093332641 | 275:98 | 2.84868087417602 | 1.23274324349535 | 0.000563699483511204 | LIHC |
| HECW2 | 0.654401467881434 | 113:260 | 0.966595823667566 | 0.44304069051374 | 0.019088354686196 | LIHC |
| ITCH | 1.60222599197604 | 304:69 | 2.50387021254999 | 1.02526405581909 | 0.0169591558533134 | LIHC |
| NEDD4L | 2.03595958209234 | 258:115 | 3.00584653739227 | 1.37902297018455 | 4.01971165657766e-05 | LIHC |
| RPF1 | 2.2263346601215 | 262:111 | 3.33707626212467 | 1.48530199178084 | 3.69082358486761e-06 | LIHC |
| SMURF1 | 1.87545534300671 | 329:44 | 3.38813287024381 | 1.03813305980509 | 0.00711854786277899 | LIHC |
| SMURF2 | 1.94039871607851 | 324:49 | 3.40364278833594 | 1.10621102492366 | 0.00271685919380527 | LIHC |
| WWP1 | 1.35251907404232 | 133:240 | 1.92328628186456 | 0.951136532765593 | 0.101719784714367 | LIHC |
| WWP2 | 2.26900792417269 | 314:59 | 3.87521354515953 | 1.3285453562654 | 4.78619494518329e-05 | LIHC |
| HECW1 | 1.32553416225222 | 52:463 | 2.09595281297982 | 0.838301704321147 | 0.274640583763892 | LUAD |
| HECW2 | 1.45299925139767 | 458:57 | 2.41049099115644 | 0.875840993518637 | 0.0910135842678707 | LUAD |
| ITCH | 1.38225693462149 | 240:275 | 1.84690711135666 | 1.03450477913084 | 0.0287472459872946 | LUAD |
| NEDD4L | 0.641478313080274 | 269:246 | 0.857191402225211 | 0.480049642453368 | 0.00275303594212406 | LUAD |
| RPF1 | 1.43880387607484 | 296:219 | 1.93112695818935 | 1.07199404214676 | 0.0132019220096907 | LUAD |
| SMURF1 | 1.51530332303879 | 144:371 | 2.0685180886415 | 1.11004306581646 | 0.0160129233534758 | LUAD |
| SMURF2 | 0.633602393420231 | 84:431 | 0.941869644797542 | 0.426228826000798 | 0.00875807856554967 | LUAD |
| WWP1 | 1.71838049255636 | 441:74 | 2.71324383947497 | 1.0883030394237 | 0.00425190445277157 | LUAD |
| WWP2 | 0.575170704887456 | 370:145 | 0.789378285279755 | 0.419091006086505 | 0.00251987232080786 | LUAD |
| HECW1 | 1.32707448767758 | 274:227 | 1.7424123988984 | 1.01074045212152 | 0.0381065916491988 | LUSC |
| HECW2 | 1.37057868608597 | 108:393 | 1.87697442421308 | 1.00080529096218 | 0.069897324013283 | LUSC |
| ITCH | 1.39398466692522 | 243:258 | 1.82478680246452 | 1.06488782634671 | 0.0157898545608096 | LUSC |
| NEDD4L | 1.19189988091514 | 180:321 | 1.58050237287865 | 0.898844158986014 | 0.233195432293303 | LUSC |
| RPF1 | 0.726424757008109 | 279:222 | 0.951321808039775 | 0.554694450536791 | 0.0219289664988013 | LUSC |
| SMURF1 | 0.76922489422709 | 117:384 | 1.0716311115567 | 0.552155430649206 | 0.0945036638836146 | LUSC |
| SMURF2 | 1.31769815486412 | 360:141 | 1.78300715226955 | 0.973820225635205 | 0.0570996363149104 | LUSC |
| WWP1 | 1.39959670357642 | 50:451 | 2.18131099311837 | 0.898024600271058 | 0.192967525572364 | LUSC |
| WWP2 | 1.55444027697846 | 157:344 | 2.06248724466837 | 1.17153916027315 | 0.00445175784546425 | LUSC |
| HECW1 | 4.23947188858205 | 8:78 | 7.27917601969038 | 2.46911488957811 | 0.000157949035496929 | MESO |
| HECW2 | 5.80046686142318 | 8:78 | 10.3180005121649 | 3.26084649548144 | 0.000324764519903042 | MESO |
| ITCH | 1.65379617819473 | 57:29 | 2.81008963931157 | 0.973293435465473 | 0.033307580026697 | MESO |
| NEDD4L | 1.95168629828253 | 50:36 | 3.24578583363998 | 1.17354613093252 | 0.00285570753808773 | MESO |
| RPF1 | 1.56940750847429 | 66:20 | 2.92615436333811 | 0.841732739227637 | 0.0969216341740016 | MESO |
| SMURF1 | 2.31773549055901 | 63:23 | 4.41920664653292 | 1.21557968066765 | 0.000623278256794979 | MESO |
| SMURF2 | 2.69571835183654 | 33:53 | 4.30074439654248 | 1.68968363669099 | 1.30891945469003e-05 | MESO |
| WWP1 | 0.521839013625544 | 17:69 | 1.06025838991895 | 0.256839237237725 | 0.0211009292614214 | MESO |
| WWP2 | 0.507193287537498 | 35:51 | 0.83949578197202 | 0.306428020780299 | 0.0024047179589104 | MESO |
| HECW1 | 1.33992229841992 | 286:93 | 1.83051025060987 | 0.980814920432567 | 0.0456637248279963 | OV |
| HECW2 | 1.28528909700596 | 191:188 | 1.66577060117801 | 0.991714022155368 | 0.0547564292853008 | OV |
| ITCH | 1.3652283299943 | 218:161 | 1.78523047682867 | 1.04403796440335 | 0.0178143587542351 | OV |
| NEDD4L | 0.632432306214004 | 324:55 | 0.881544326114471 | 0.453715837190048 | 0.0193917217359184 | OV |
| RPF1 | 0.769628093684066 | 231:148 | 1.00451508969041 | 0.589665012170525 | 0.0630854367293087 | OV |
| SMURF1 | 1.51737873267795 | 286:93 | 2.11598714240074 | 1.08811541064993 | 0.00474634640256166 | OV |
| SMURF2 | 0.701553295025215 | 269:110 | 0.918816088908171 | 0.535664352967078 | 0.014347632214697 | OV |
| WWP1 | 1.49751608451588 | 149:230 | 1.93837432444884 | 1.15692536529105 | 0.00233503519824629 | OV |
| WWP2 | 1.30254068564296 | 83:296 | 1.74182196654987 | 0.974044575356808 | 0.0907334415851601 | OV |
| HECW1 | 1.62557029794604 | 84:94 | 2.44645116420637 | 1.08012734209702 | 0.0212008031734461 | PAAD |
| HECW2 | 0.614967115790878 | 141:37 | 0.967765450119968 | 0.390781209907081 | 0.0574535956782303 | PAAD |
| ITCH | 1.89017190618119 | 93:85 | 2.85625927989275 | 1.25084926990618 | 0.00190772130217198 | PAAD |
| NEDD4L | 1.99030158120604 | 35:143 | 3.33249838942634 | 1.18868786155158 | 0.0352722535384093 | PAAD |
| RPF1 | 2.18348063895778 | 29:149 | 3.60512432298615 | 1.32244751458514 | 0.0158981581752041 | PAAD |
| SMURF1 | 3.94130728795599 | 26:152 | 6.67328301588757 | 2.32777526460549 | 0.00110565524132322 | PAAD |
| SMURF2 | 1.80094274926354 | 76:102 | 2.71273949954164 | 1.19561601350698 | 0.00691207906974078 | PAAD |
| WWP1 | 2.79036631098837 | 27:151 | 4.71333924483197 | 1.65193798813359 | 0.00603480585697047 | PAAD |
| WWP2 | 2.12316937000779 | 24:154 | 3.6664192837156 | 1.2294960900301 | 0.0348805387036704 | PAAD |
| HECW2 | 0.313099220643861 | 67:116 | 1.34797321721552 | 0.0727248291848808 | 0.0897595518024986 | PCPG |
| ITCH | 2.12588512076742 | 164:19 | 16.0958764006192 | 0.28077921538503 | 0.341697445654217 | PCPG |
| NEDD4L | 0.103865355922284 | 23:160 | 1.11495122492144 | 0.00967577049087765 | 6.78174558523814e-05 | PCPG |
| RPF1 | 0.286818205697596 | 32:151 | 1.51604300449347 | 0.0542627635731705 | 0.0443983128072647 | PCPG |
| SMURF1 | Inf | 20:163 | Inf | Inf | 0.270125966364876 | PCPG |
| SMURF2 | 4.26548563341793 | 164:19 | 35.0952213367299 | 0.518428634893746 | 0.0293421276609855 | PCPG |
| WWP1 | 0 | 157:26 | 0 | 0 | 0.210304665903164 | PCPG |
| WWP2 | 0.305182499245323 | 83:100 | 1.22190044615027 | 0.0762225418110436 | 0.120846234250268 | PCPG |
| HECW1 | 0.278997060529761 | 49:447 | 1.53398802774933 | 0.0507431338290516 | 0.0276900223843073 | PRAD |
| HECW2 | 2.22442677103872 | 207:289 | 7.68632937724392 | 0.643749989996914 | 0.181546929728873 | PRAD |
| ITCH | 4.40737133363923 | 133:363 | 16.4889608960582 | 1.17805616709471 | 0.116282590151337 | PRAD |
| NEDD4L | 4.41014836711936 | 151:345 | 16.4970030949875 | 1.17896617391768 | 0.121989064749101 | PRAD |
| RPF1 | 3.42446109664284 | 446:50 | 18.3408762524752 | 0.639387870077236 | 0.0352993361535785 | PRAD |
| SMURF1 | 3.68464759200369 | 95:401 | 14.431443707488 | 0.940767129917438 | 0.158934161484776 | PRAD |
| SMURF2 | 3.84487437958824 | 105:391 | 14.8813395393191 | 0.993395719233114 | 0.145557192491842 | PRAD |
| WWP1 | 0.239340702454864 | 332:164 | 0.90672801457066 | 0.0631765765820218 | 0.135429154867554 | PRAD |
| WWP2 | 0.247153371307243 | 375:121 | 0.943845890954668 | 0.0647190283222515 | 0.141826036122603 | PRAD |
| HECW1 | 0.22736718939802 | 23:143 | 0.786683207072969 | 0.0657136676491433 | 7.513474178511e-05 | READ |
| HECW2 | 0.370289032149232 | 23:143 | 1.33363687396181 | 0.102812069767307 | 0.0252231526751814 | READ |
| ITCH | 0.246899081850784 | 137:29 | 0.598194507124553 | 0.101905243014991 | 0.0340472987825696 | READ |
| NEDD4L | 0.323289642734191 | 84:82 | 0.698511290180446 | 0.149627063396787 | 0.00672175850813361 | READ |
| RPF1 | 0.381272948326329 | 31:135 | 1.01087081609875 | 0.14380577499158 | 0.012342824943407 | READ |
| SMURF1 | 0.387842175439494 | 21:145 | 1.36455170041656 | 0.110235143896504 | 0.0328673579172615 | READ |
| SMURF2 | 0.402151116018199 | 89:77 | 0.873811299391746 | 0.185080600613953 | 0.0197204885277811 | READ |
| WWP1 | 0.404496284972152 | 105:61 | 0.873267923743972 | 0.187362022705236 | 0.0300321564414583 | READ |
| WWP2 | 0.42495610928891 | 26:140 | 1.21621543627123 | 0.148483310963087 | 0.0368521950202201 | READ |
| HECW1 | 1.63960297374042 | 107:156 | 2.43411617101241 | 1.10442465462949 | 0.0174509631938897 | SARC |
| HECW2 | 1.62557171379335 | 194:69 | 2.57244495542294 | 1.02722641007904 | 0.0202450577695179 | SARC |
| ITCH | 1.77842256977815 | 129:134 | 2.63759831796787 | 1.19911618655151 | 0.00488218741806834 | SARC |
| NEDD4L | 2.01307592256282 | 110:153 | 2.98532160359524 | 1.35746670145077 | 0.000820920107040268 | SARC |
| RPF1 | 1.98830435396636 | 49:214 | 3.14001490896716 | 1.25902402332922 | 0.0142772614434459 | SARC |
| SMURF1 | 1.69051549248449 | 212:51 | 2.84227654557038 | 1.00547662569427 | 0.0195985854562277 | SARC |
| SMURF2 | 1.60418292553074 | 131:132 | 2.3820469635648 | 1.08033254504487 | 0.0186694279462567 | SARC |
| WWP1 | 1.36572997671179 | 161:102 | 2.05407864786663 | 0.908055965250596 | 0.121205758005528 | SARC |
| WWP2 | 1.71043205154287 | 42:221 | 2.82349800731541 | 1.03615366306804 | 0.0756502808311839 | SARC |
| HECW1 | 1.41608975027727 | 94:377 | 1.90825316606483 | 1.05086170771338 | 0.0330245576712445 | SKCM |
| HECW2 | 0.831167621306033 | 370:101 | 1.1203546027285 | 0.616625854907963 | 0.241518652776353 | SKCM |
| ITCH | 0.547511752993964 | 63:408 | 0.924378373926465 | 0.324292657770864 | 0.00313566194376591 | SKCM |
| NEDD4L | 1.25189223889323 | 240:231 | 1.63811199869419 | 0.956732005534669 | 0.0952835872860339 | SKCM |
| RPF1 | 0.585645782155714 | 117:354 | 0.828521603231591 | 0.413967458203872 | 0.000346215690057217 | SKCM |
| SMURF1 | 1.44338257355392 | 74:397 | 2.05738125382788 | 1.01262381474651 | 0.0719984388939272 | SKCM |
| SMURF2 | 0.476871945384388 | 82:389 | 0.743087851498427 | 0.306029565462721 | 9.60009777417614e-06 | SKCM |
| WWP1 | 0.575700879052787 | 80:391 | 0.887341951434129 | 0.373510461898582 | 0.00182549854315095 | SKCM |
| WWP2 | 0.782007529007375 | 393:78 | 1.10083527890417 | 0.555519783153184 | 0.191072772710379 | SKCM |
| HECW1 | 1.51193364467672 | 148:227 | 2.10815060629809 | 1.08433588144803 | 0.0198568088300035 | STAD |
| HECW2 | 1.90567916715282 | 80:295 | 2.80104746708969 | 1.29651965230476 | 0.00634974354567519 | STAD |
| ITCH | 1.44410555168153 | 116:259 | 2.04668535257544 | 1.01893573517453 | 0.0537916553895288 | STAD |
| NEDD4L | 1.47241047461235 | 37:338 | 2.48913840993335 | 0.870981138331406 | 0.213505247089566 | STAD |
| RPF1 | 0.508991560804631 | 335:40 | 0.89756527856941 | 0.288639071893755 | 0.0752401388372295 | STAD |
| SMURF1 | 1.36798980572558 | 157:218 | 1.90595068417691 | 0.981870162803957 | 0.0733056266368709 | STAD |
| SMURF2 | 1.62344066426159 | 176:199 | 2.25130590901612 | 1.17068035037936 | 0.00463151151865926 | STAD |
| WWP1 | 1.49811100019994 | 322:53 | 2.44178461722283 | 0.91913781137366 | 0.0600662781850855 | STAD |
| WWP2 | 0.647922736972274 | 315:60 | 0.975179365721831 | 0.430488880140427 | 0.0687218138693784 | STAD |
| HECW1 | 0.0682476344720357 | 13:126 | 3.75330060222615 | 0.00124097164193723 | 0.000308543392066762 | TGCT |
| HECW2 | 0 | 74:65 | 0 | 0 | 0.0965181392666661 | TGCT |
| ITCH | 4.05649936570437 | 124:15 | 164.003559632748 | 0.100334328967054 | 0.182514599982818 | TGCT |
| NEDD4L | Inf | 58:81 | Inf | Inf | 0.168346615620771 | TGCT |
| RPF1 | 0.115139222773744 | 36:103 | 1.08520324037338 | 0.0122161823036766 | 0.023915470341255 | TGCT |
| SMURF1 | 7.48399589646055 | 100:39 | 65.4350288310473 | 0.855966530141771 | 0.0398738309430868 | TGCT |
| SMURF2 | 0.219919497349303 | 13:126 | 10.7008557970032 | 0.00451969321256667 | 0.144688690448633 | TGCT |
| WWP1 | 0.226211222445452 | 23:116 | 2.82993757707945 | 0.0180822070333705 | 0.102107163873133 | TGCT |
| WWP2 | Inf | 28:111 | Inf | Inf | 0.196898864339548 | TGCT |
| HECW1 | 3.5359186487749 | 270:240 | 9.45237664895885 | 1.32270656947756 | 0.0194306129343953 | THCA |
| HECW2 | 8.26605388771003 | 144:366 | 23.0107039049054 | 2.96938534157401 | 0.0136465873830023 | THCA |
| ITCH | Inf | 59:451 | Inf | Inf | 0.124459986115289 | THCA |
| NEDD4L | 0.227992473727641 | 286:224 | 0.607475935596712 | 0.0855681106534525 | 0.010928006968059 | THCA |
| RPF1 | 0.261550947604819 | 218:292 | 0.701923466358158 | 0.0974591981486382 | 0.012151769592496 | THCA |
| SMURF1 | 2.59041251763924 | 450:60 | 12.1039441801692 | 0.55438433221924 | 0.0866738792263968 | THCA |
| SMURF2 | 0.316580631377659 | 182:328 | 0.93281767933283 | 0.107441462982518 | 0.0153202554587274 | THCA |
| WWP1 | 3.15036034932606 | 260:250 | 8.39630578975126 | 1.18204012325519 | 0.035609801944794 | THCA |
| WWP2 | 0.482204106511149 | 54:456 | 2.46683558347858 | 0.0942587345072786 | 0.244120429344071 | THCA |
| HECW1 | 1.98899808974659 | 99:20 | 10.1577576012209 | 0.389467198994794 | 0.316483348173996 | THYM |
| HECW2 | 0 | 103:16 | 0 | 0 | 0.127594232156385 | THYM |
| ITCH | 0.223254532451095 | 54:65 | 0.832894167937057 | 0.0598426404922593 | 0.0395648298576763 | THYM |
| NEDD4L | 0.206150223595089 | 17:102 | 1.3430204547261 | 0.0316435349430121 | 0.00831722857107386 | THYM |
| RPF1 | 0 | 71:48 | 0 | 0 | 0.00430611558899052 | THYM |
| SMURF1 | 2.75153132138429 | 78:41 | 11.2661883278535 | 0.672004088005622 | 0.113626137319063 | THYM |
| SMURF2 | 0.274384486753823 | 46:73 | 1.07538037935562 | 0.0700095036290986 | 0.0489878624071693 | THYM |
| WWP1 | Inf | 24:95 | Inf | Inf | 0.135869210678891 | THYM |
| WWP2 | 0.18502669248797 | 12:107 | 1.93365389192074 | 0.0177047594070888 | 0.00716592401167826 | THYM |
| HECW1 | 1.83274840842823 | 489:55 | 3.7536438824578 | 0.894854928645189 | 0.0334270058361572 | UCEC |
| HECW2 | 2.66780193061349 | 56:488 | 4.87014985634873 | 1.4613856556605 | 0.0256701701770202 | UCEC |
| ITCH | 2.02055635734653 | 113:431 | 3.27736125287801 | 1.24571192438072 | 0.0200086206166258 | UCEC |
| NEDD4L | 0.691267087248627 | 70:474 | 1.2559341177308 | 0.380473927069178 | 0.165104840758027 | UCEC |
| RPF1 | 2.60363849431832 | 80:464 | 4.44962725478101 | 1.52348343376682 | 0.0111603826735885 | UCEC |
| SMURF1 | 2.06717908850384 | 116:428 | 3.30593155334894 | 1.29259463330956 | 0.0127294511040339 | UCEC |
| SMURF2 | 2.10663178171762 | 489:55 | 4.50320825168875 | 0.985496831526386 | 0.0083855878702882 | UCEC |
| WWP1 | 1.71031899050361 | 94:450 | 2.83945205523685 | 1.03019561252401 | 0.078716867484102 | UCEC |
| WWP2 | 0.617982871851823 | 204:340 | 0.943211834976141 | 0.404896138640889 | 0.0196424315696841 | UCEC |
| HECW1 | 3.01837207447055 | 44:12 | 8.45109682478982 | 1.07803403141938 | 0.00180016224874158 | UCS |
| HECW2 | 0.402011774929495 | 48:8 | 0.874558182863288 | 0.184794414309684 | 0.053111802693509 | UCS |
| ITCH | 0.520371768698666 | 34:22 | 1.01984312841413 | 0.265518068528495 | 0.062748786791158 | UCS |
| NEDD4L | 0.611516806805842 | 41:15 | 1.22651641024122 | 0.304890176669113 | 0.194341804779986 | UCS |
| RPF1 | 0.492809218417327 | 50:6 | 1.22120966457561 | 0.198869148191268 | 0.218065291017497 | UCS |
| SMURF1 | 2.23860871737159 | 47:9 | 6.73930490962594 | 0.743603243464201 | 0.0470518754119352 | UCS |
| SMURF2 | 0.587750224890665 | 36:20 | 1.15174437157637 | 0.299936631238942 | 0.127555305704258 | UCS |
| WWP1 | 1.67040729603713 | 12:44 | 3.58226116922258 | 0.778910415194435 | 0.241473819929667 | UCS |
| WWP2 | 0.493097210140753 | 7:49 | 1.54693210059113 | 0.157178753065943 | 0.106018245206583 | UCS |
| HECW2 | 4.49495528797037 | 52:28 | 11.4510689576046 | 1.76443117368837 | 9.30960355689514e-05 | UVM |
| ITCH | 4.26238751273855 | 72:8 | 19.8392532260259 | 0.915757619592057 | 0.000794215568270751 | UVM |
| NEDD4L | 2.71959005536507 | 67:13 | 8.87584116420439 | 0.833292296742395 | 0.0200816523228536 | UVM |
| RPF1 | 2.16783978254124 | 68:12 | 6.46916947857868 | 0.726450178547642 | 0.0778648627145395 | UVM |
| SMURF1 | 0.568927105238056 | 29:51 | 1.38282298396733 | 0.234070488289051 | 0.171124628072382 | UVM |
| SMURF2 | 3.17976680498852 | 8:72 | 10.8824656688057 | 0.929101661500257 | 0.23110840828887 | UVM |
| WWP1 | 4.54811750068967 | 66:14 | 14.5344752385568 | 1.42319364549232 | 6.43390459996729e-05 | UVM |
| WWP2 | 0.236950719652272 | 52:28 | 0.537346174784175 | 0.104486913982929 | 0.00401572720044963 | UVM |

Supplementary Table 8. CMAP analysis results of the most important small molecule chemical in relation to NEDD4 members.

| rank | cmap name | mean | n | enrichment | p | specificity | percent non-null |
| --- | --- | --- | --- | --- | --- | --- | --- |
| 1 | withaferin A | -0.842 | 4 | -0.984 | 0 | 0.0082 | 100 |
| 2 | ellipticine | -0.807 | 4 | -0.974 | 0 | 0 | 100 |
| 4 | flupentixol | -0.656 | 4 | -0.92 | 0.00008 | 0 | 100 |
| 5 | perphenazine | -0.642 | 5 | -0.873 | 0.00008 | 0 | 100 |
| 6 | cephaeline | -0.529 | 5 | -0.831 | 0.00032 | 0.0723 | 100 |
| 7 | felbinac | 0.489 | 4 | 0.857 | 0.00056 | 0.0175 | 100 |
| 8 | iloprost | 0.711 | 3 | 0.928 | 0.00066 | 0.0066 | 100 |
| 9 | gossypol | -0.272 | 6 | -0.74 | 0.00066 | 0.0152 | 50 |
| 10 | semustine | -0.661 | 4 | -0.856 | 0.00074 | 0.0074 | 100 |
| 11 | parbendazole | 0.367 | 4 | 0.833 | 0.00115 | 0.0076 | 75 |
| 12 | parthenolide | -0.729 | 4 | -0.837 | 0.00123 | 0.0276 | 100 |
| 13 | F0447-0125 | -0.529 | 4 | -0.836 | 0.00125 | 0.0086 | 100 |
| 14 | paclitaxel | 0.453 | 6 | 0.693 | 0.00215 | 0 | 83 |
| 15 | securinine | 0.284 | 4 | 0.815 | 0.00223 | 0.026 | 50 |
| 16 | skimmianine | -0.495 | 4 | -0.813 | 0.00229 | 0.0053 | 100 |
| 18 | pyrimethamine | -0.467 | 5 | -0.737 | 0.00262 | 0.0067 | 80 |
| 19 | cloperastine | -0.345 | 6 | -0.682 | 0.00268 | 0.0252 | 66 |
| 21 | podophyllotoxin | 0.329 | 4 | 0.796 | 0.00338 | 0.0429 | 50 |
| 22 | chenodeoxycholic acid | 0.306 | 4 | 0.794 | 0.00348 | 0.011 | 50 |
| 23 | isocarboxazid | 0.398 | 5 | 0.726 | 0.00352 | 0 | 80 |
| 24 | indometacin | -0.345 | 8 | -0.591 | 0.00357 | 0 | 75 |
| 25 | benzydamine | -0.467 | 4 | -0.792 | 0.0038 | 0.0062 | 100 |
| 26 | STOCK1N-35215 | -0.69 | 3 | -0.876 | 0.00389 | 0 | 100 |
| 27 | pergolide | 0.221 | 4 | 0.784 | 0.0041 | 0.017 | 50 |
| 28 | oxantel | 0.43 | 4 | 0.784 | 0.00412 | 0 | 75 |
| 29 | Prestwick-857 | 0.399 | 4 | 0.782 | 0.00418 | 0.0258 | 75 |
| 30 | carbenoxolone | 0.339 | 4 | 0.776 | 0.00477 | 0.0065 | 75 |
| 31 | trapidil | 0.488 | 3 | 0.863 | 0.00481 | 0 | 100 |
| 32 | albendazole | 0.561 | 3 | 0.855 | 0.00583 | 0 | 100 |
| 33 | moxisylyte | -0.277 | 5 | -0.692 | 0.00619 | 0.0191 | 60 |
| 34 | eucatropine | -0.362 | 6 | -0.64 | 0.00622 | 0.0469 | 83 |
| 35 | harmalol | 0.447 | 3 | 0.847 | 0.00693 | 0 | 100 |
| 37 | staurosporine | -0.49 | 4 | -0.757 | 0.00716 | 0 | 100 |
| 38 | brinzolamide | 0.427 | 4 | 0.748 | 0.0077 | 0.0394 | 75 |
| 39 | thiamphenicol | 0.419 | 5 | 0.684 | 0.00809 | 0.142 | 60 |
| 40 | isoxicam | 0.324 | 5 | 0.683 | 0.00813 | 0.0915 | 60 |
| 41 | diethylstilbestrol | 0.255 | 6 | 0.621 | 0.009 | 0.1105 | 50 |
| 42 | antimycin A | -0.369 | 5 | -0.669 | 0.00927 | 0.0522 | 60 |
| 43 | ciclopirox | -0.38 | 4 | -0.738 | 0.00937 | 0.0571 | 50 |
| 44 | viomycin | 0.289 | 4 | 0.737 | 0.00937 | 0.1041 | 50 |
| 45 | niclosamide | -0.527 | 5 | -0.665 | 0.00989 | 0.0826 | 80 |
| 46 | phenazopyridine | -0.395 | 4 | -0.735 | 0.00993 | 0.0317 | 75 |
| 47 | metronidazole | 0.363 | 5 | 0.665 | 0.01117 | 0.0719 | 60 |
| 48 | fluspirilene | -0.447 | 4 | -0.725 | 0.01166 | 0.062 | 75 |
| 49 | menadione | -0.583 | 2 | -0.924 | 0.01189 | 0.0345 | 100 |
| 50 | melatonin | 0.344 | 4 | 0.713 | 0.01363 | 0.0256 | 50 |
