# Supplementary material for: Expression and regulatory network of E3 ubiquitin ligase NEDD4 family in cancers
Source: BMC Cancer. 2023 Jun 8;23:526. doi: 10.1186/s12885-023-11007-w (PMC10251597; doi:10.1186/s12885-023-11007-w)

Figure 5A

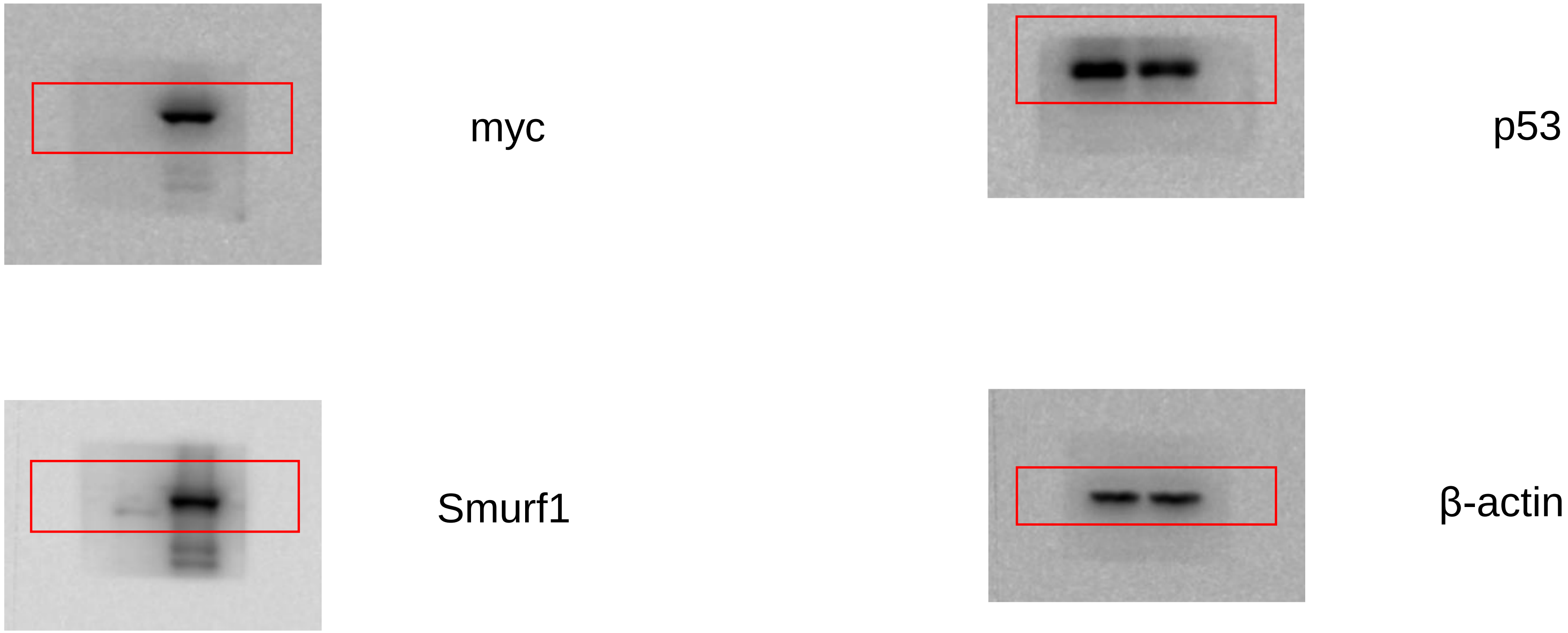

Figure 5B

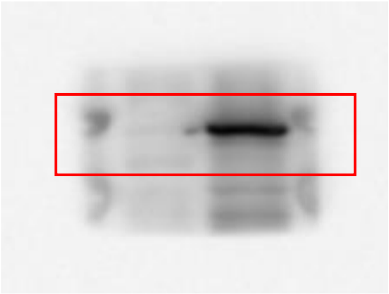

Flag

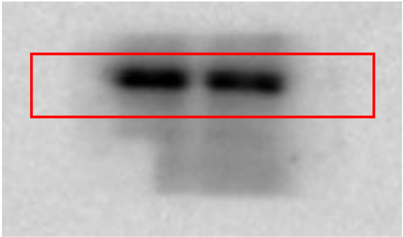

p53

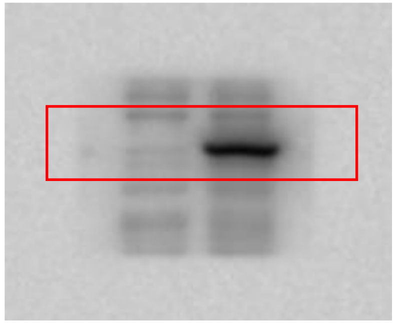

Smurf2

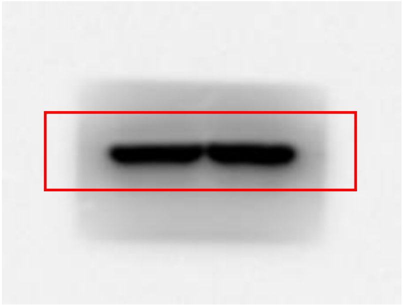

β-actin

Figure 5C

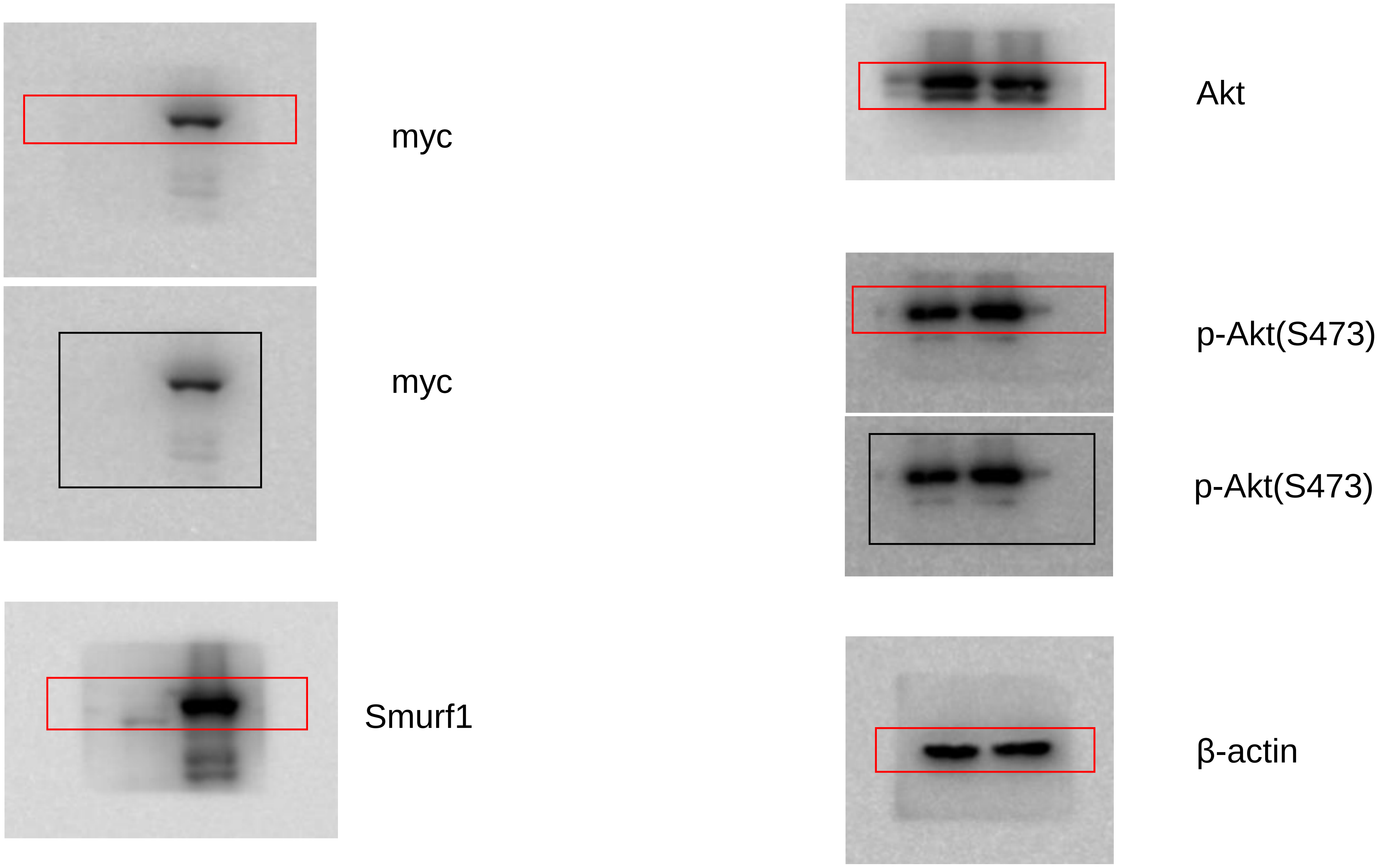

Figure 5D

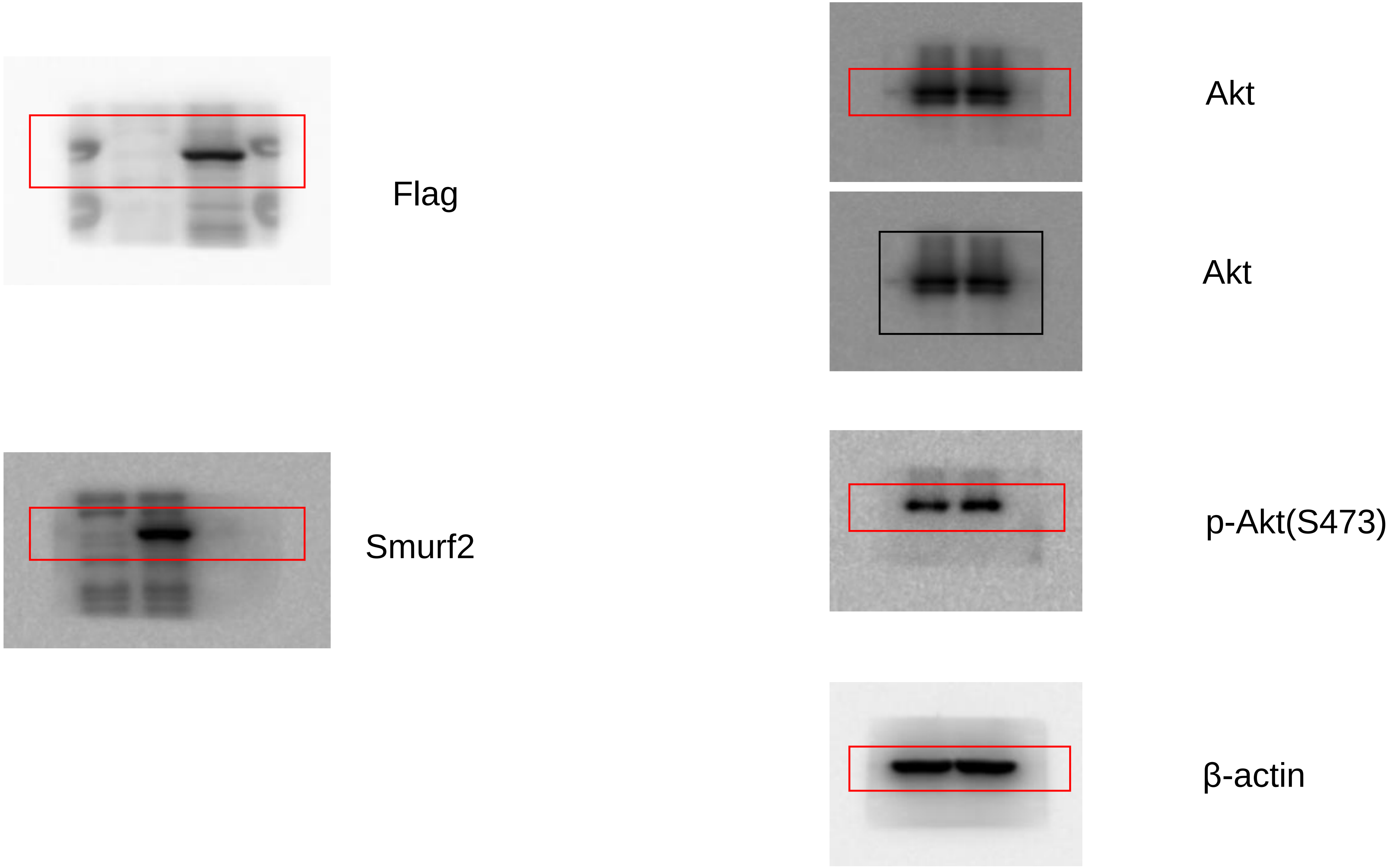

Figure 5E

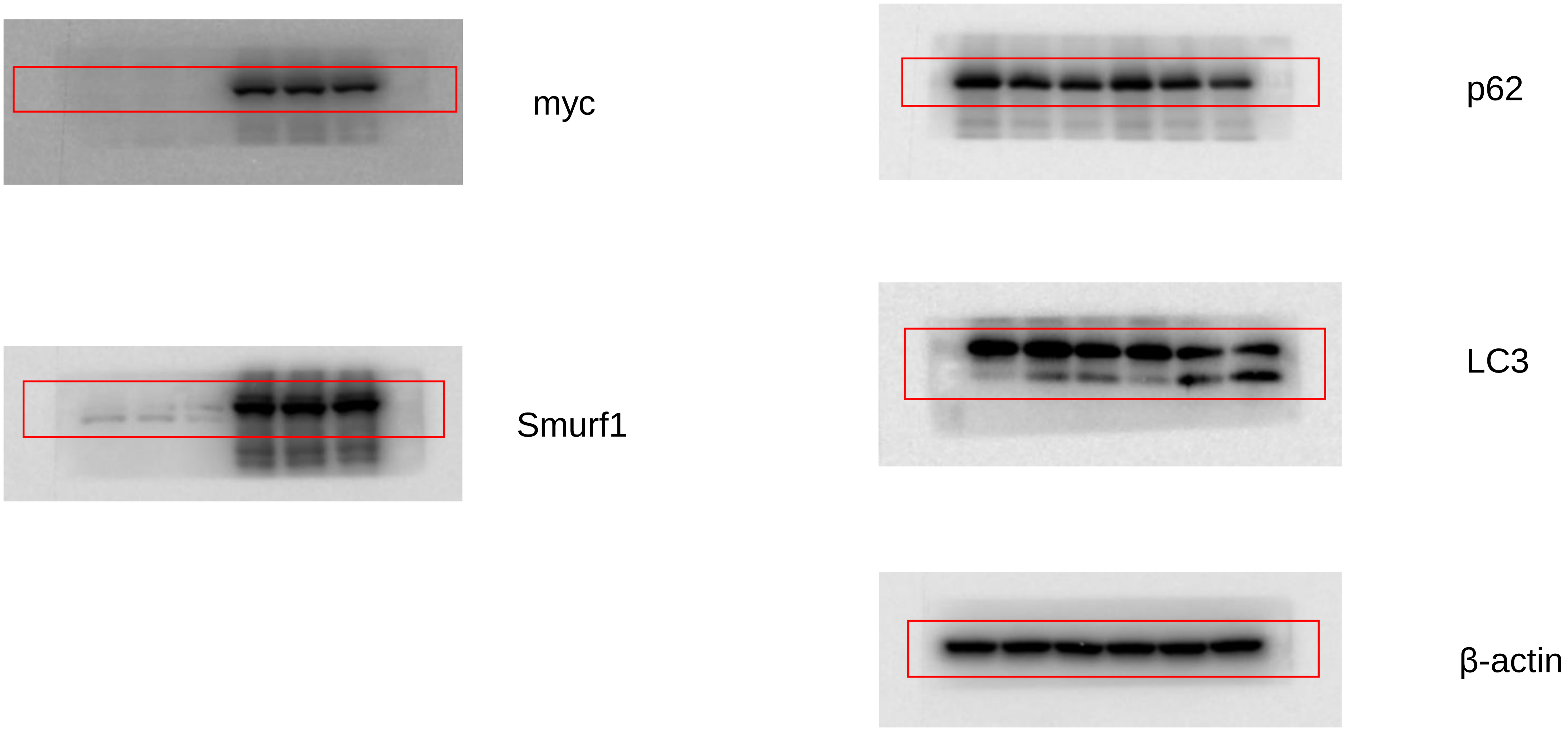

Figure 5F

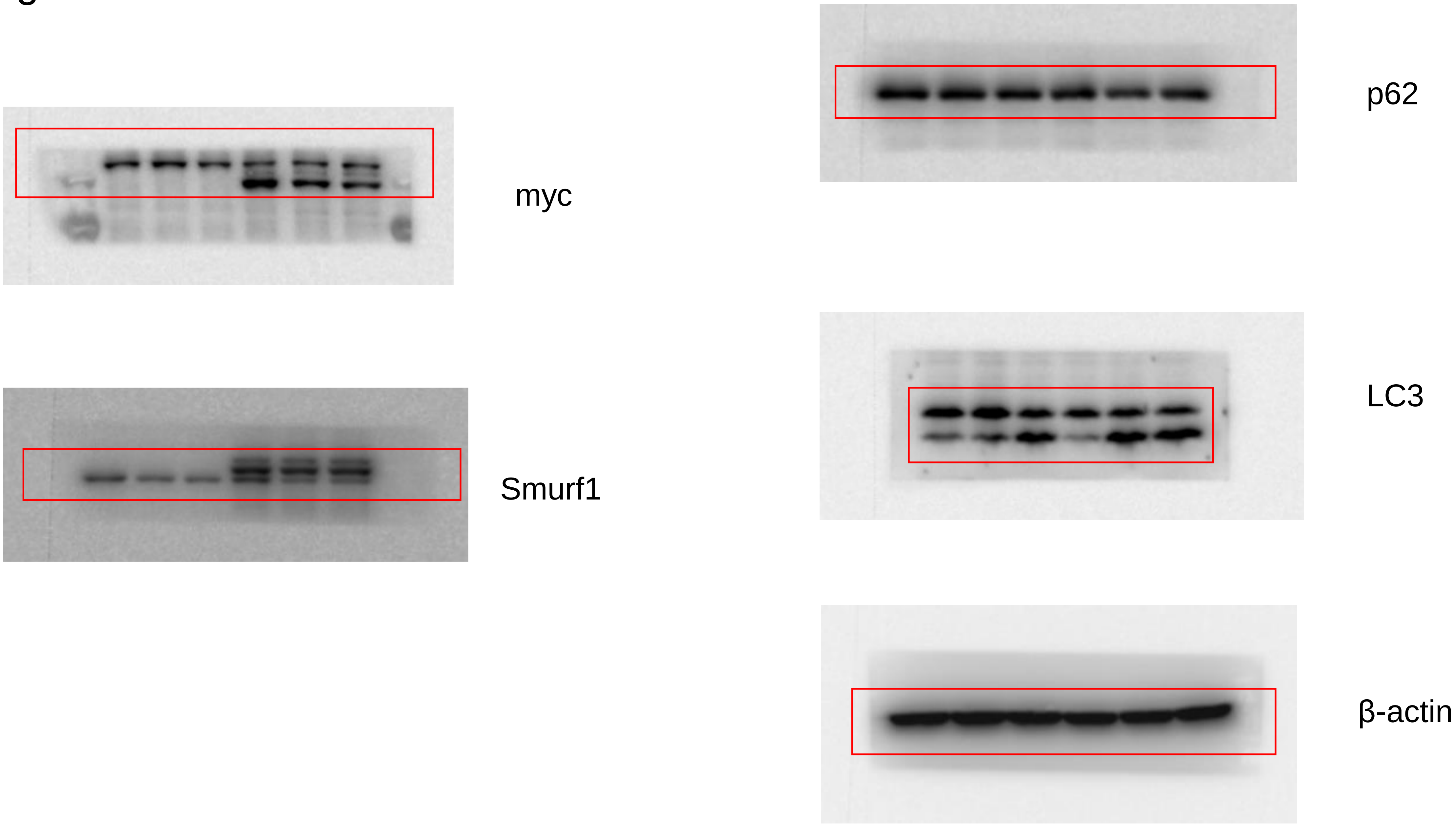

Figure 5G

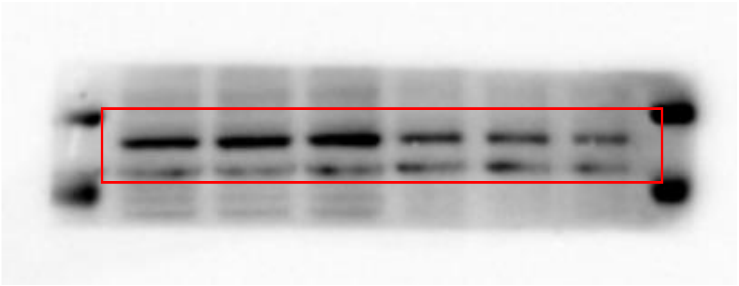

Smurf1

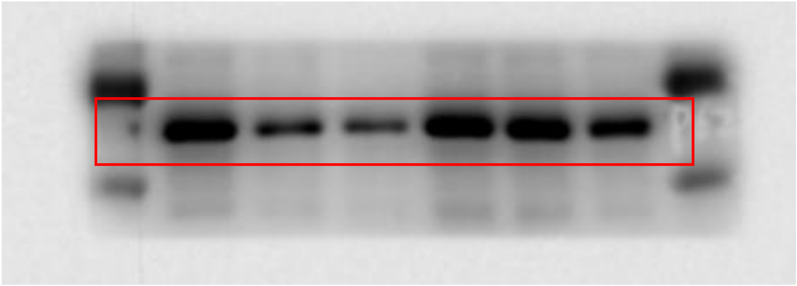

p62

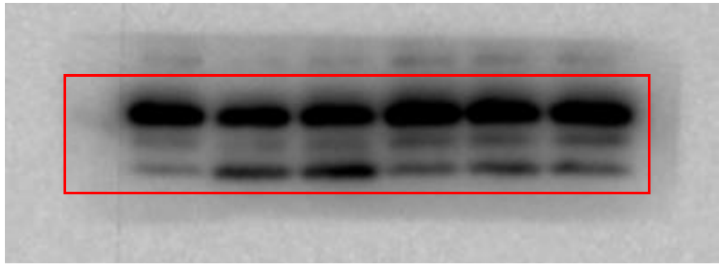

LC3

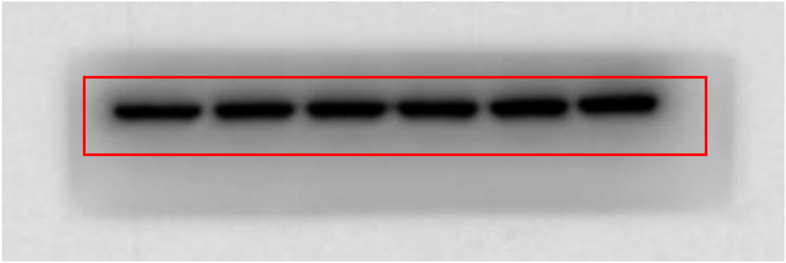

β-actin

Figure 5H

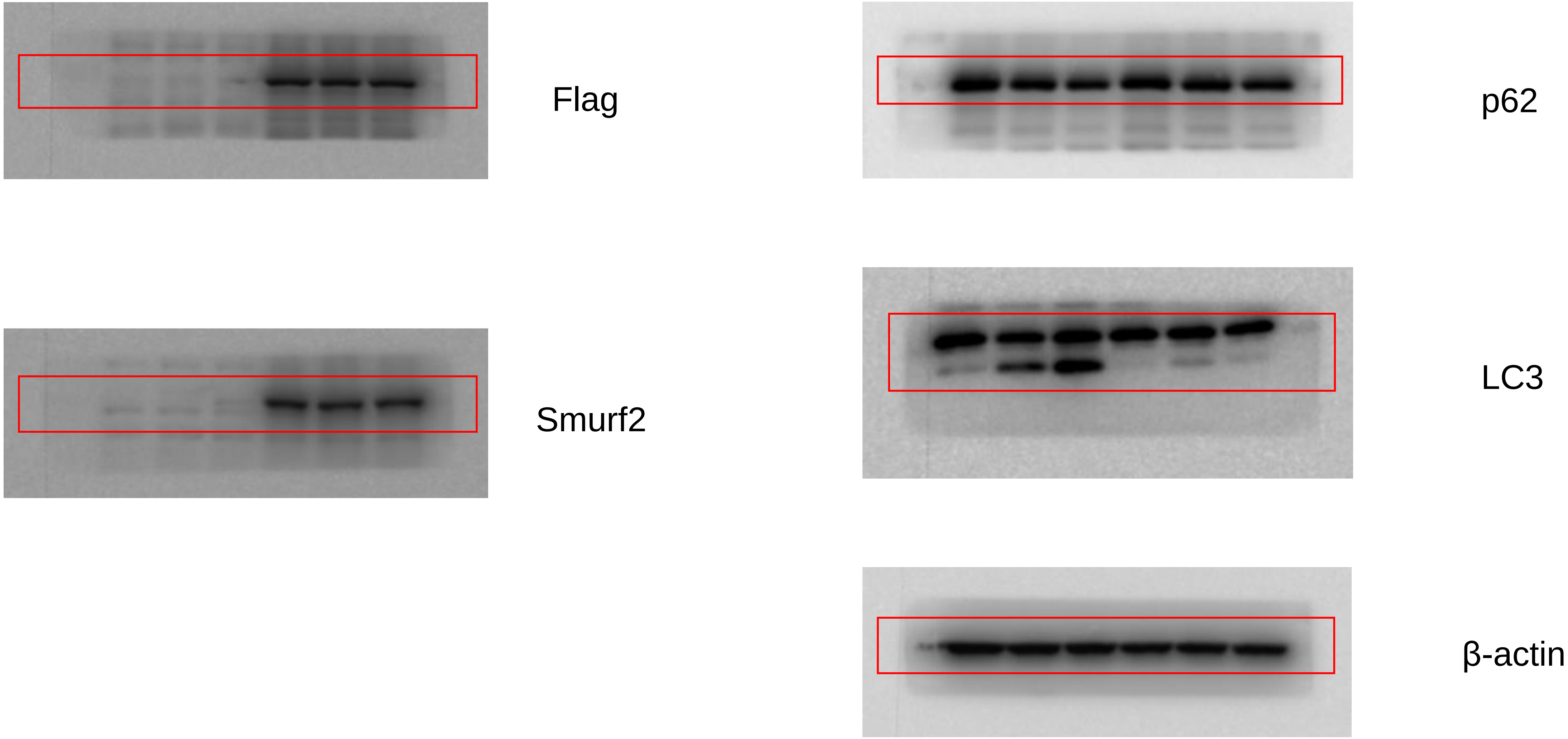

Figure 5I

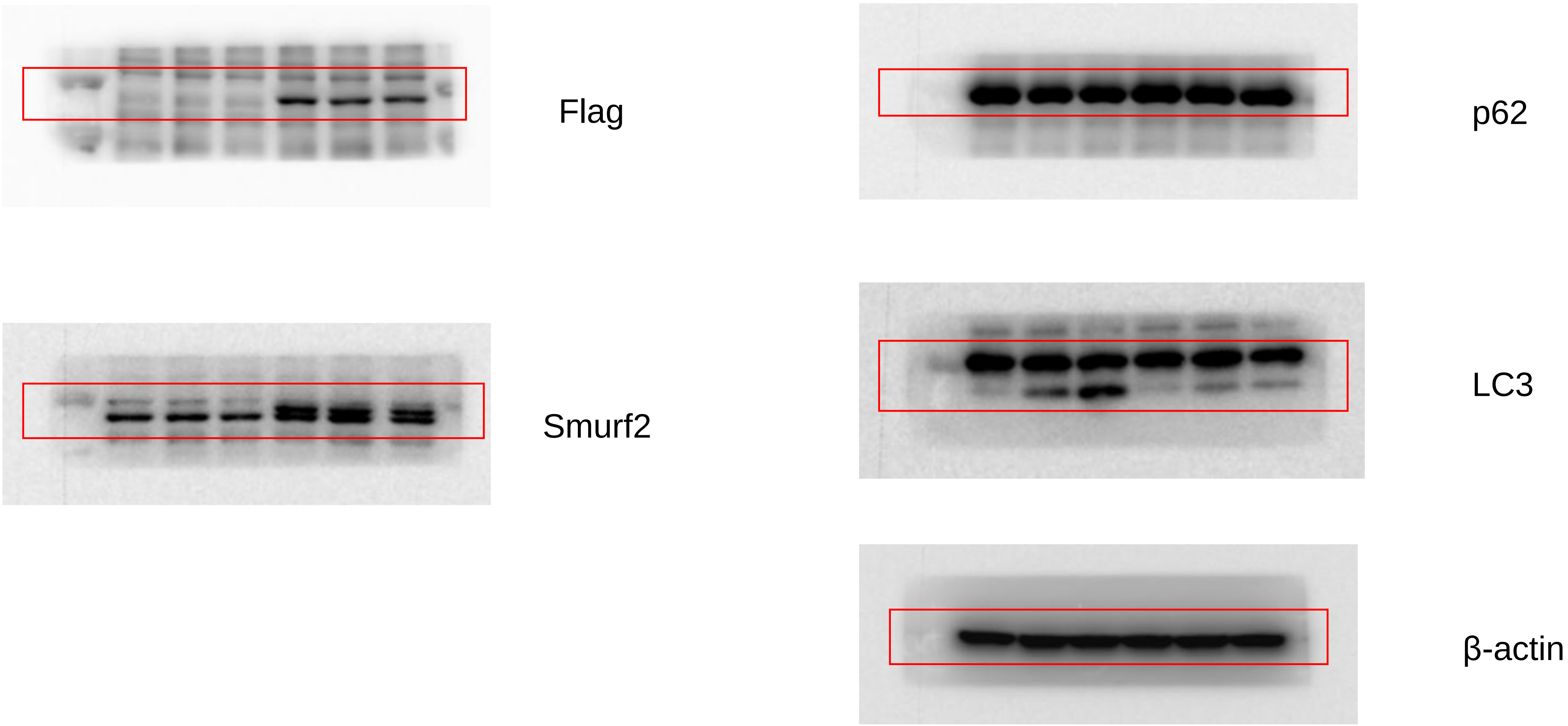

Figure 5J

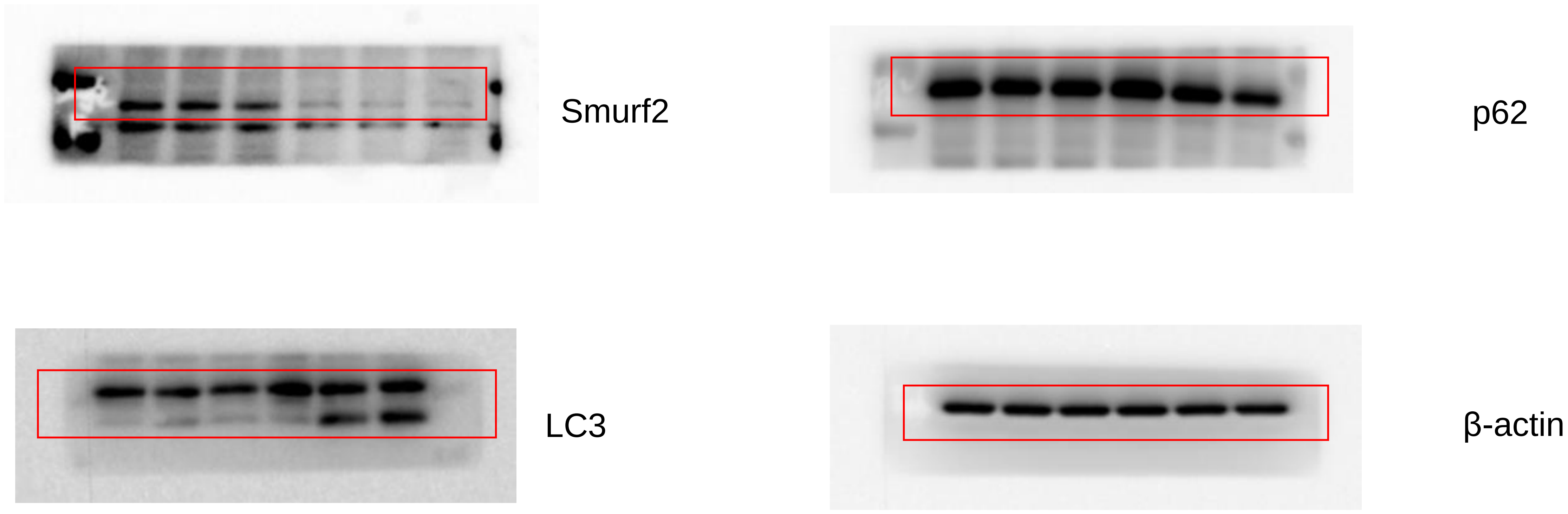

Supplement: Supplementary file 2 — Supplementary Material 2 [file 12885_2023_11007_MOESM2_ESM.pdf]
